# Comparative Genomics of NAC Transcriptional Factors in Angiosperms: Implications for the Adaptation and Diversification of Flowering Plants

**DOI:** 10.1371/journal.pone.0141866

**Published:** 2015-11-16

**Authors:** Alejandro Pereira-Santana, Luis David Alcaraz, Enrique Castaño, Lenin Sanchez-Calderon, Felipe Sanchez-Teyer, Luis Rodriguez-Zapata

**Affiliations:** 1 Unidad de Biotecnología, Centro de Investigación Científica de Yucatán, Mérida, Yucatán, México; 2 Unidad de Bioquímica y Biología Molecular de Plantas, Centro de Investigación Científica de Yucatán, Mérida, Yucatán, México; 3 Departamento de Ecología de la Biodiversidad, Instituto de Ecología, Universidad Nacional Autónoma de México, México City, D.F., México; 4 Laboratorio de Biología Molecular de Plantas, Unidad Académica de Ciencias Biológicas, Universidad Autónoma de Zacatecas, Zacatecas, Zacatecas, México; Institute of Genetics and Developmental Biology, Chinese Academy of Sciences, CHINA

## Abstract

NAC proteins constitute one of the largest groups of plant-specific transcription factors and are known to play essential roles in various developmental processes. They are also important in plant responses to stresses such as drought, soil salinity, cold, and heat, which adversely affect growth. The current knowledge regarding the distribution of NAC proteins in plant lineages comes from relatively small samplings from the available data. In the present study, we broadened the number of plant species containing the NAC family origin and evolution to shed new light on the evolutionary history of this family in angiosperms. A comparative genome analysis was performed on 24 land plant species, and NAC ortholog groups were identified by means of bidirectional BLAST hits. Large NAC gene families are found in those species that have experienced more whole-genome duplication events, pointing to an expansion of the NAC family with divergent functions in flowering plants. A total of 3,187 NAC transcription factors that clustered into six major groups were used in the phylogenetic analysis. Many orthologous groups were found in the monocot and eudicot lineages, but only five orthologous groups were found between *P*. *patens* and each representative taxa of flowering plants. These groups were called basal orthologous groups and likely expanded into more recent taxa to cope with their environmental needs. This analysis on the angiosperm NAC family represents an effort to grasp the evolutionary and functional diversity within this gene family while providing a basis for further functional research on vascular plant gene families.

## Introduction

Environmental abiotic stresses, such as water deficit, soil salinity, and extreme temperatures, have adverse effects on the growth, development, and grain yields of crops worldwide [1, 2]. To cope with these stresses, land plants have developed molecular mechanisms to protect cellular activities and maintain plant integrity [3]. Transcriptional control under environmental stresses plays a major role in plant adaptation. During abiotic stress, many transcription factors (TF) are involved in the induction of stress-responsive genes that play important roles under stress conditions such as drought, cold, heat, soil salinity, and flooding. Among the proteins involved in abiotic stress responses are the AP2/ERF [4, 5, 6], ABF [7, 8], HSF [9], bZIP [7, 10], MYB [11], NAC [12, 13, 14], and the WRKY families [10,15].

NAC proteins constitute one of the largest groups of plant TFs. They are ubiquitously expressed across plant organisms and are known to participate in various developmental processes and stress responses. The NAC acronym is derived from three genes that were initially discovered to contain a particular domain (the NAC domain): NAM (no apical meristem), ATAF1/2 (Arabidopsis transcription activator factor 1/2) and CUC2 (cup-shaped cotyledon, [16, 17]). Typically, the NAC family contains a highly conserved NAC DNA-binding domain located in the N-terminal region and consists of approximately 150–160 amino acids divided into five sub-domains (A-E) encoding a twisted β-sheet surrounded by a few helical elements [18]. The C-terminal domain contains the transcriptional activation region (TAR), which is non-conserved among plants and can act as either a transcriptional activator or repressor [19, 20]. In many cases, NAC TFs are inactively stored in the cytoplasm and translocated into the nucleus after stimulation. The NAC's TAR contains transmembrane motifs (TMM) that help anchor the protein to the plasma membrane and provide an efficient method for gene regulation, which is an adaptive strategy that allows for prompt responses to environmental changes [21, 22, 23]. NAC TFs play several essential roles in plant development [24], senescence [25], auxin signaling [26], floral time control [27], floral morphogenesis [28, 29], lateral root development [26], nutrient mobilization [30], leaf margin development [31], fruit ripening [32], longevity control [33], embryo development [34], postembryonic shoot meristem formation [35], organ boundary formation [35], leaf movement [36], vascular element formation [37], and tolerance to multiple biotic [38, 39] and abiotic stresses [25, 26, 27, 40, 41], and so on. Although NAC TFs have been characterized and described in several plants species, such as Arabidopsis [19], populus [42], citrus [43], rice [19], and barley [44] (ranging from 16–289 NAC sequences [45]), a systematic classification is still lacking, and genome-wide analysis of this gene family keeps being applied within the plant lineage.

Polyploidy or whole-genome duplication (WGD) is now recognized as a major evolutionary force in angiosperm genome development [46]. In fact, researchers have long recognized that polyploidy is an inseparable part of angiosperm biology. Over time, polyploids may become diploidized, such that they behave like diploids at the cytogenetic and genetic levels [47]. Given this genome duplication trend, the genome conservation in terms of gene number and chromosomal organization is astonishing. After the duplication of all genes by the WDG process, redundant copies get lost (fractionation), retained, or fixed with modified functional properties in contrast to the original genes. Therefore, the gene family size can vary greatly between species due to the number of WGD events that their genomes have experienced through evolution [48, 49]. Despite the relatively recent origin of angiosperms during the Early Cretaceous period ~130 to >385 million years (MYr) ago, [50, 51, 52] land plant evolution has become extremely diverse both in morphological and ecological terms. Over the past 130 and 150 MYr, angiosperms have diversified to occupy all habitable terrestrial and many aquatic environments [53].

The current knowledge regarding the NAC distribution in the plant lineage comes from a relatively small sampling [1, 49, 54, 55]. To shed new light into the evolutionary history of the NAC family in angiosperms, we broadened the number of plant species to assess the origin and evolution of this family in the present study. We conducted a comprehensive phylogenetic analysis through this lineage, and defined the orthologous groups (OGs) between 24 green plant genomes in order to examine the groups that were identified from the early-diverging plant *Physcomitrella patens* as an out-group genome to inspect the species-specific expansion along the angiosperms lineage. Our results revealed that an increased number of predicted NAC family members with probable divergent functions are present in those species with more WGD events, suggesting that a significant expansion of the NAC family occurred shortly before the rapid radiation of flowering plants.

The lack of knowledge regarding the origin of the NAC family has impeded our understanding of the evolution of this family across the history of plant development. Therefore, given our conception of the evolutionary history of the NAC family as part of a much more diverse set of evolutionarily plants, we can update our knowledge of this family and determine the expansion of this lineage over time. The main focus of this work was to conduct a comparative genomics analysis of the NAC family across the angiosperms.

## Materials and Methods

### NAC gene family searches and retrieval

A Hidden Markov Model (HMM) profile was constructed for the identification of new members of the NAC TF family in flowering plants. The HMM profile was built using the Plant Transcription Factor Database v3.0 [45], in which 436 NAC proteins were predicted from five plants (*O*. *sativa*, *V*. *vinifera*, *A*. *thaliana*, *S*. *moellendorffii*, and *P*. *patens)*. Sequences were retrieved, and a multiple alignment was completed with Clustal Omega [56]. Subsequently, the alignment was manually curated, and the positions that contained gaps or missing data were not considered to build and calibrate the HMM model by HMMER package (version 3.1; [57]). The model was calibrated with the required cut-off values and was used to detect NAC sequences in 24 plant species. Gene models of the plant species analyzed in this study are shown in S1 Table. The EMBOSS 3.0 suite [58] was used to manipulate the sequences. The genome size and the ploidy level of each plant were obtained from published genome data and the Plant DNA C-values database [59].

### Sequence analysis, multiple sequence alignments and evolutionary model testing

Amino acid sequences were subjected to a motif scan in the Pfam database v27.0 [60] to confirm the presence of the NAC domain. All retrieved sequences were clustered using the CD-HIT clustering program [61] with an identity cut-off of 0.7 in order to exclude isoforms and reduce sequence redundancy. Multiple sequence alignments were completed using MUSCLE [62] and were manually curated. They were then tested with the ProtTest 2.4 statistical package [63] to find the best evolutionary model for the maximum likelihood (ML) analysis. The MEME software [64] was used to identify conserved motifs in the amino acid sequences of interest, with an occurrence parameter for a single motif of “one per sequence” and the maximum number of motifs. The TMHMM Server v. 2.0 (http://www.cbs.dtu.dk/services/TMHMM/) was used to predict the TMM for all retrieved NAC sequences. HMM logos of the NAC domain were plotted with the Skylign tool [65] for each of the 24 plant species as well as for the global set of all the NAC sequences. The amino acid alignments of the selected sequences were visualized using BOXSHADE v3.31C (http://boxshade.sourceforge.net/).

### Defining orthologous groups (OGs)

We used a reciprocal best BLAST hits (BBH) approach [66, 67] to identify possible orthologous groups among the 24 plant genomes. Significant hits were filtered according to the following criteria: we retained all potential OGs where the BLASTP bit-score of the compared proteins was more than 200 and had sequence coverage of at least 50% of their lengths [66]. Protein pairs were labeled as orthologs when both sequences were each other's bi-directional best hit. Moss *P*. *patens*, a basal lineage of land plants, was used as a reference outgroup to perform all the comparison analysis and to establish the BBH approach. Additionally, *O*. *sativa* and *V*. *vinifera* NAC proteins were used as reference sequences for analysis into monocot and eudicot species, respectively [48]. Each NAC protein of *P*. *patens* was compared with the NAC protein set of the basal Magnoliopsida (*Vitis vinifera*) and Liliopsida (*Oryza sativa*) species. On the other hand, a BLASTP search was performed against *Coccomyxa subellipsoidea*, *Chlamydomonas reinhardtii*, *Ostreococcus lucimarinus*, *Cyanidioschyzon merolae* (http://www.phytozome.net, [68]*)*, Cyanobacteria (NCBI taxid:1117), and Alphaproteobacteria (NCBI taxid:28211) to determine the origin of the NAC proteins. A Venn diagram was created on R (v3.0.1; [69]) using the Vennerable package (https://r-forge.r-project.org/projects/vennerable/).

### Evolutionary analysis and Angiosperm tree of life

The phylogenetic reconstructions were RAxML v.8.0.26 suite [70] with a JTT substitution model and 100 parametric bootstrap (BS) replicates. The gamma-distributed rates were estimated from the dataset. The topology of the best-scoring tree was visualized in FigTree v1.4 (http://www.molecularevolution.org) and PhyloWidget (http://www.phylowidget.org, [71]). An evolutionary tree of the 24 plants used in the analysis of the NAC proteins was constructed with the PhyloT tree generator (http://phylot.biobyte.de/index.html) and manually corrected according to the APG III [72] tree topology.

## Results and Discussion

### Genome-wide identification of NAC genes

**Fig 1:**
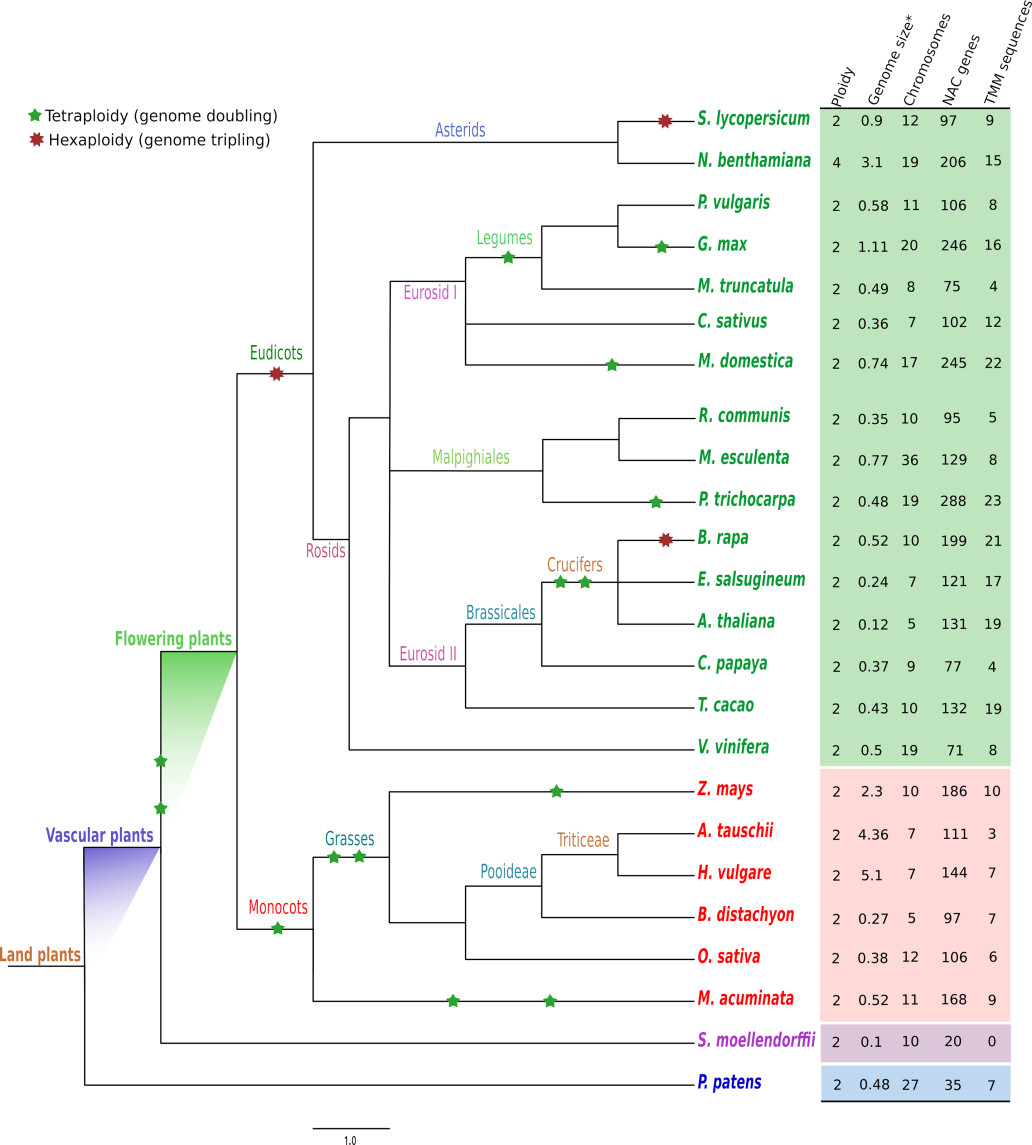
Phylogenetic relationships among 24 land plant species and the distribution of NAC proteins identified in this study, based on the HMM-generated profile. The total number of NAC proteins identified in each genome; the plant genome size, ploidy and chromosome number of each species; and the number of NAC sequences with Transmembrane motifs (TMM) for each genome are indicated on the right in colored squares. Ancient WGDs are represented by colored stars (details were taken from CoGepedia, https://genomevolution.org). Species names are color-coded as follows: blue–Moss, purple–pseudofern, red—monocots, and green—eudicots. * Genome size in Gb.

We analyzed whole-genome sequences of algae and bacteria, and no sequences containing the NAC domain were found. In early diverging plants, we identified 35 NAC proteins in the moss *P*. *patens* used as an out-group, and seven of those sequences contained a TMM, providing evidence of this basal mechanism in green plants. However, no TMMs were found in the 20 predicted NACs of *S*. *moellendorffii*, the earliest evolutionary branch of vascular plants for which genome information is available. In contrast, studied flowering plants possess an average of 140 NAC genes, with a minimum of 71 and a maximum of 288 genes, suggesting a lineage-specific expansion into the NAC family shortly before the rapid radiation in Angiosperms history [53, 73], likely due to the numerous WGDs that have occurred through the angiosperm lineage [46, 74]. According to our results, the extent and nature of this expansion varies between the different plant lineages. A total of 3,187 sequences were identified and retrieved from 24 plant species (Fig 1). These species included; 16 eudicots plants (Class Magnoliopsida), 6 monocots (Class Liliopsida), one fern (Lycopodiopsida), and a moss (Bryopsida). All identified NAC homologs host the expected NAM domain (Pfam PF02365), while some contained additional domains. The complete list of NAC proteins analyzed in this study, the additional annotation of each sequence, and the sequences used in the phylogenetic analysis are available in S2 Table. A HMM LOGO of the NAC domain was completed for each species to analyze the prevalence of specific residues per lineage (S1 Fig).

The monocot lineage revealed a similar number of NAC sequences in the six analyzed species, regardless of their genome size. Maize was found to contain the greatest number of NAC sequences from the analyzed monocot species, with 186 NAC genes. However, it also was found to contain an additional WGD event compared to the other grass species. Regarding the TMM sequences, no correlation was found between the number of these sequences and the genome size, as seen in the case of *A*. *tauschii*, which only possesses three TMM sequences in a 4.3 Gb genome, and *B*. *distachtion*, which has seven TMM sequences in a 0.27 Gb genome. The regulated activation of preexisting dormant TFs by TMM is a versatile system for accurate stress signal perception and transduction. It provides an efficient method for gene regulation and is considered to be an adaptive mechanism in response to environmental changes [23, 75]. On the other hand, 43 of the 3,187 NAC sequences contained additional domains, mainly in the C-terminal region (see S2 Table). The Ataus42 NAC sequence of *A*. *tauschii* contained a zinc-binding motif in its reverse transcriptase domain (zf-RVT, Pfam PF13966) and a Reverse transcriptase-like domain (RVT-3, Pfam PF13456) on its C-terminus. The Zmays146 NAC sequence has four TMM helixes and an additional motif called MatE, which is annotated as a Multi antimicrobial extrusion protein (Pfam PF01554) and is involved in the detoxification of secondary metabolites including alkaloids. This last mechanism is poorly studied in plant systems and can help us understand how plants have evolved to cope with toxins in their environment [76, 77].

It is well known that eudicot plants have a smaller genome size than monocot plants; nevertheless, we found that the number of NAC sequences was greater in the eudicot lineage. In the case of the TMM sequences, we found a 1:2 ratio in the monocot plants compared to the eudicot plants. In some cases, the TMM sequences further increased for those species that experienced more WGD events, such as some rosid species (e.g., apple tree, populus, and *B*. *rapa*).

A detailed study of genome size reveals a dynamic pattern of genome evolution. An increase in the genome size occurred over the course of vascular plant development, with an independent genome reduction in *Selaginella* and angiosperms and subsequent increases within some groups of monocots [78]. Hence, there appears to be a greater relative expansion of the NAC gene family (140:35:20) in the spermatophyta (seed plants) compared to moss and fern species [79], demonstrating that duplicated genes contributed to the expansion of large families in grapevine species. These duplications were produced by WGD and tandem, proximal, retrotransposed, and DNA-based transposed duplications. In Arabidopsis, it has been shown that several gene families have expanded by tandem duplication [80], retaining many of their genes after WGD and transposition [81]. Each of these gene family expansion modes creates paralogs that potentially duplicates the function of the original gene. If retained, this functional duplication sets the stage for biased gene expansion and subsequent subfunctionalization [82]. Some gene families in eudicot plants are much larger, suggesting differential expansion (e.g., the NAC family in Poplar is represented by 288 members, soybean by 246 sequences, and apple tree by 245 NAC sequences). On the other hand, some rosid species, such as grapevine, papaya tree, and soybean, contain the lowest NAC sequence numbers of their families, with 71, 77, and 75 sequences, respectively. The Eurosid group contains many economically important flowering plants, such as legumes and brassicales; all of these species share a palaeo-hexaploid common ancestor that emerged after divergence from the monocots and before the radiation of the eurosids [83]. Grapevine and papaya tree serve as evolutionary important model plants for fruit tree genomics [83, 84]. With only 24,746 predicted genes for the papaya tree genome (the lowest predicted gene number for the eudicot plants [85]) and 26,346 predicted genes for the grapevine [83], these two plants appear to have experienced only one WGD shared by all monocot and eudicot lineages. This observation allows us to better understand the ancestral traits of this lineage. However, the grapevine genome is also the closest to the palaeo-hexaploid ancestral arrangement by far [83], suggesting that the duplication patterns of ancestral genes and the addition of new activities in their functions were common occurrences in the evolution of this genome [79]. For these reasons, the papaya tree was proposed as an excellent outgroup for Arabidopsis, while grapevine was proposed as a useful outgroup for Brassicales [86]. A resent report [85] demonstrated a reduction in most gene families and biosynthetic pathways in the papaya plant and highlighted the value of this plant as model to study complex pathways and networks. Papaya contains no recent WGDs, explaining its lower opportunities for subfunctionalization and implying that papaya genes may be more representative of basal angiosperms than Arabidopsis genes and those of many other sequenced plants. Here, we found that the papaya tree's genome contains 77 NAC proteins. This study is the first to report and characterize the NAC family in the papaya tree genome. All the identified NAC genes were named using the prefix CpNAC, and the complete set of the papaya's NAC proteins is available in S3 Table.

The major difference between the NAC protein family numbers within the angiosperms and within the outgroup *P*. *patens* could be due to common selective pressures such as environmental stresses, which may have guided the regulation of plant growth and development [87]. Another explanation for the NAC gene family differences could be the release of selective pressures due to gene redundancy. The ease of sub-functionality among the duplicated TFs could also be a contributing factor. After duplication, TFs are capable of acquiring different functions from their ancestors and could be naturally selected for their new functions, thus resulting in the indispensability of both copies [82]. Gene family number differences suggest the genes in question may be involved in plant-specific regulatory functions. The differences also highlight the importance of TF duplication, due its contribution to the regulatory novelties that could be involved in development, and responses to external stimuli [88, 89]. Other authors [79] have found that large gene families in grapevine and others plant species are essential and are involved in the basic processes of plant growth. These authors have also suggested that these families have undergone large expansions during their evolutionary history, as supported by our report for the NAC family.

### Orthologous groups and duplications in the NAC family

Orthologous Groups (OGs) were calculated by means of a BBH using all 24 analyzed proteomes. Then, a matrix containing presence information from all predicted OGs was built and used to define the shared core of OGs for the NAC proteins. According to previous work [90], evolutionary genomics compares a set of genomes with an outgroup genome. To test the minimum content of orthologous proteins in monocots, we used rice (*O*. *sativa)* as a reference because its phylogenetic position is closest to the tested plants in terms of the hypothetical basal monocots genome. *O*. *sativa* is thought to have an original monocot chromosome number (12) and has not evolved through independent nested chromosome fusion (NCF) events [48]. On the other hand, grapevine (*V*. *vinifera*) was used as a pivotal genome for eudicots because the grapevine genome contains the ancient genome-wide duplications shared by all eudicots and has the lowest number of NCF events [48, 83, 91, 92]. *P*. *patens* was used as an outgroup for comparing these two pivotal genomes for monocots and eudicots and to identify OGs in the *S*. *moellendorffii* plant. The comparison across multiple outgroups is a useful tool that permits us to unambiguously discrimination between gene additions and losses in any group [86]. We ruled out the 39 homologous genes that did not have significant hits according to the criteria previously established (see Materials and Methods).

**Fig 2:**
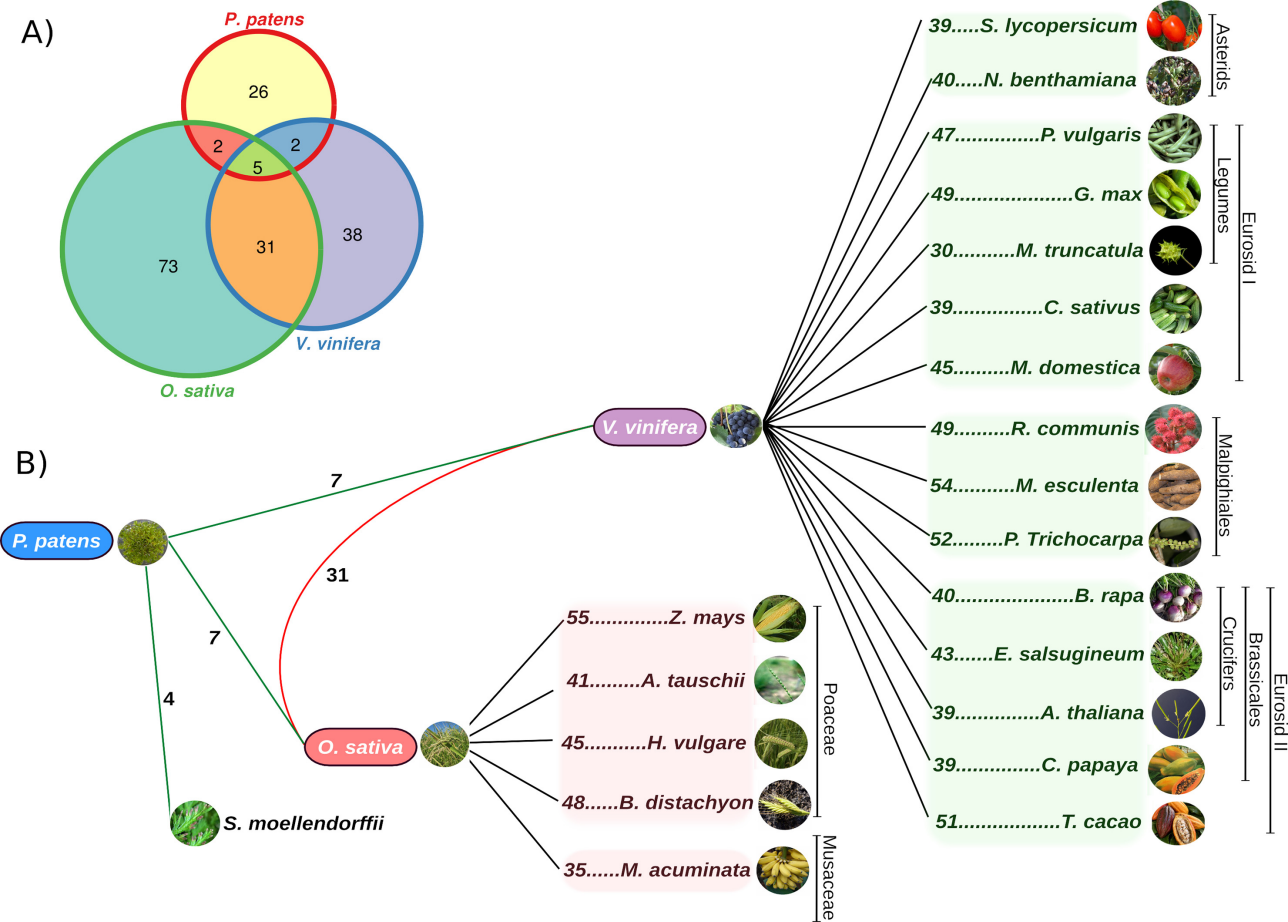
Schematic representation of NAC OGs shared among Angiosperms. A) Venn diagram showing the orthologous gene number shared between the *P*. *patens* outgroup and rice and grapevine genomes as the selected representatives for monocot and eudicot plants, respectively. The total overlap consisted of five proteins that define the basal core of the NAC genes. B) The number of orthologous proteins detected by means of BBH in 24 green plants. Green lines indicate the OGs shared between the moss *P*. *paten*, *S*. *moellendorffii*, and the basal taxa of the monocot and eudicot lineages. The number below the red line indicates the number of OGs shared between the rice and grapevine species. Black lines indicate the number of OGs shared among the monocots and eudicots with their respective selected pivotal species.

Four OGs were identified between *P*. *patens* and *S*. *moellendorffii*, which branched off earlier in plant evolution. Two of the sequences in *S*. *moellendorffii* were orthologous to the PpVNS1 and PpVNS5 proteins of *P*. *patens*. These genes were found to be involved in hydroid cell differentiation by inducing cell death, water conducting and tissue support in vascular plants [54]. *PpVNS1* gene expression has been found in the rhizoid tissues of *P*. *patens*, although this gene is not shared with vascular plants. In flowering plants, we identified seven OGs shared between *P*. *patens* and each representative taxa of monocots (rice) and eudicots (grapevines) (Fig 2). Among the seven OGs shared by *P*. *patens* and rice, two of them belonging to the PpVNS related proteins (PpVNS6 and PpVNS8) were found to act as cell-specialized regulators for water-conduction and plant support. All six analyzed monocots share 24 OGs, among which are the rice characterized sequences OsNAC7 (SND-related protein), OsNAC4, OsNAC6, and OsNAC10 (stress-related proteins). However, only six OGs were shared pairwise (Osat-other species). A total of 43 NAC sequences were rice specific (not shared with other species). The complete list of OGs and their paralogous genes in monocots is available in S4 Table.

In the case of eudicot plants, we found seven OGs among the *P*. *patens* and grapevine sequences, two of which belonged to the PpVNS related proteins (PpVNS6 and PpVNS7, [54]), which play a key role in water conducting. These proteins are preferentially expressed in the midrib of the central region of newly emerged leaves and in the developing leaf midrip of *P*. *patens*. We found that the PpVNS6 protein was shared for both monocot and eudicot plants, unlike PpVNS7 and PpVNS8 proteins that were specific for eudicots and monocots, respectively. We identified 13 OGs among the 16 analyzed eudicot species (S5 Table), including the Arabidopsis-characterized sequences ANAC029 (cell death related protein), ANAC033 (SOMBRERO), ANAC037 (VND1), and ANAC040 (NTL8). Only two OGs were shared pairwise (Vvin-other species), and 10 sequences were specific to grapevine. On the other hand, we found 31 OGs shared between rice and grapevine, the two selected representative taxa for monocots and eudicots, respectively (S6 Table). All studied taxa in flowering plants host paralogous genes, showing evidence of large expansions in this family. Finally, only five OGs were shared among the *P*. *patens*-rice-grapevine (see Fig 2). We decided to call these the Basal Orthologous Groups (BOGs).

The BOGs were taken as the core NAC sequences, from which evolution progressed through different events of genome-duplication. Functional divergence events among the duplicated genes were likely the source of evolutionary innovations and specific adaptation processes for each lineage [46].

Comparative analysis of each BOG was done by sequence alignment. *P*. *patens* sequences were used as basal group to underline character changes along plant evolution. We included the orthologous sequences from the analyzed angiosperm plants with the orthologous proteins shared in rice and grapevine to broaden the scope of the analysis. Plesiomorphic amino acids were detected along the NAC domain and in the C-terminal region through diverse lineages of plants, and lineage-specific synapomorphies for monocot/eudicot species were detected in the NAC domain (S2 Fig). However, the evolutionary distance of the plant lineages and amino acids in the NAC domain were very similar, confirming the importance of this domain along its evolutionary history. Overall, the NAC domain in all five BOGs has structural conservation, giving evidence of the importance of this DNA-binding domain through the plant lineage.

Furthermore, a visual inspection of the C-terminal region of each BOG was performed using the MEME software [64], identifying several conserved motifs that could be considered as the signature of these basal groups (S3 Fig). In the case of BOG1, all its members have the signature LP-box (LP[QX]L[ED]SP) and the WQ-box (W[RA]ALD[KR][FL][VL]ASQL) that were previously defined as the responsible motifs for the transcriptional activity of gene ANAC012 (SND1; [93]). Some conserved motifs were present both in early diverging plants and in more recent flowering plant lineages, suggesting they originated before the split of the monocot and eudicot lineages. Members of each OG are supposed to be derived from the same ancestor gene, sharing well-conserved motifs, structures and likely functions [49]. Our findings reveal the presence of five ancestral proteins preserved both in monocot and eudicot lineages. These proteins probably played key roles in the adaptation and survival of flowering plant lineage and expanded through different duplication mechanisms to cope with the environment challenges.

### NAC phylogenetic analyses

**Fig 3:**
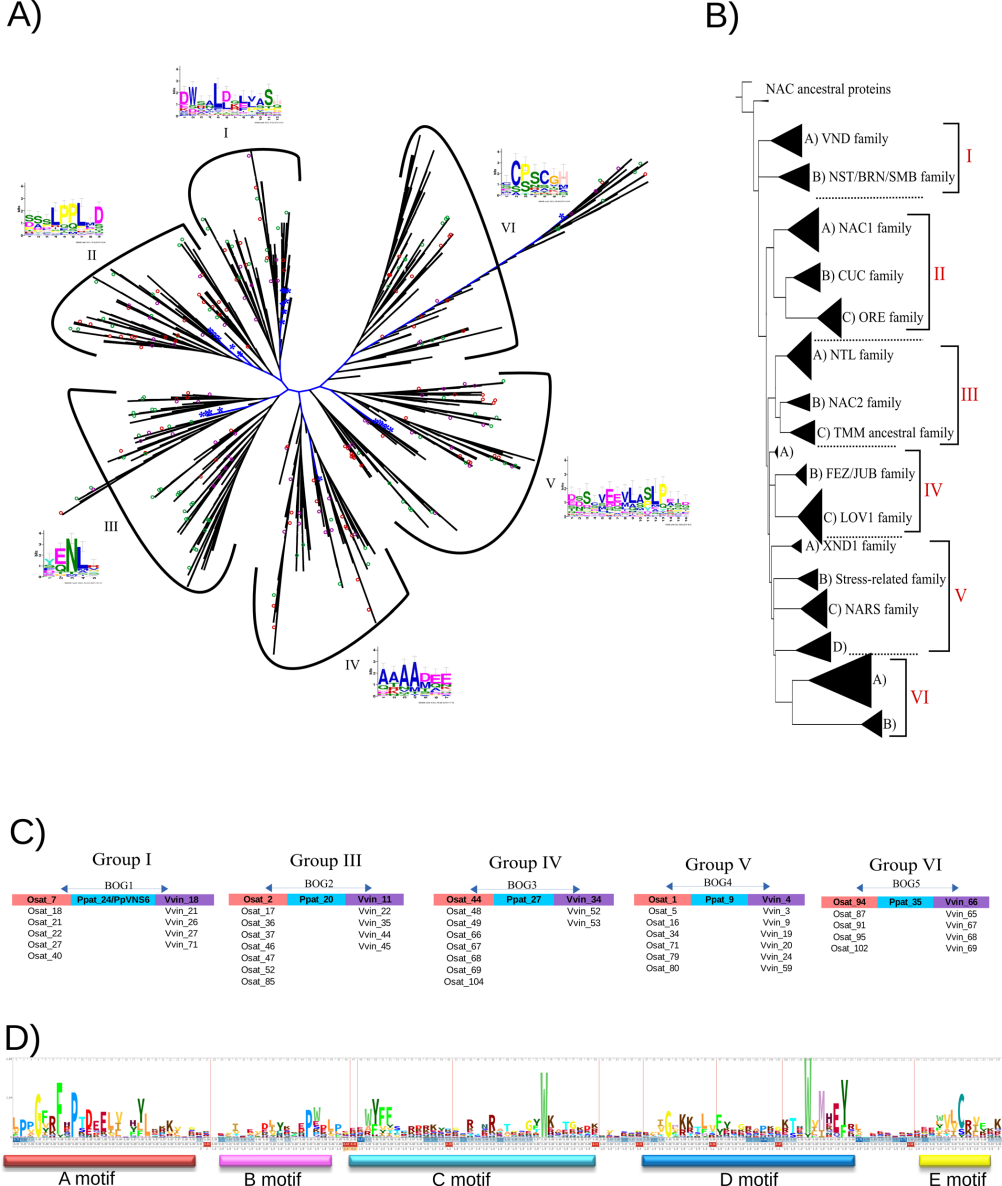
Phylogenetic relationships of the NAC gene family. A) Maximum likelihood phylogenetic tree derived from a MUSCLE alignment of the NAC domains from 24 plant species. The unrooted phylogenetic tree of the 2106 NAC proteins was clustered into six major families. Specific motifs found in the C-terminal region of the NAC proteins are shown next to each group. Blue lines and blue asterisks indicate sequences of *P*. *patens* in the tree, green circles show Arabidopsis proteins, and purple and red circles show grapevine and rice sequences, respectively. B) Schematic depiction of the proposed NAC family classification. Names of each subgroup are shown next to each clade. Triangles represent the NAC subgroups (detailed phylogenetic analysis is available in S4 Fig; branch lengths are arbitrary). C) Schematic representation of the five NAC's BOGs detected in the plant lineage and their affiliation into the major groups. Paralogous proteins for rice and grapevine are shown below each colored box. The affiliation of each BOG into the phylogenetic tree is available in S5C Fig) HMM LOGO of the NAC domain of 2106 proteins used in the phylogenetic tree.

We conducted a phylogenetic tree reconstruction from 24 green plants in order to understand the NAC family evolution and distribution through the angiosperms lineage and identify species-specific NAC gene expansions or reductions. The global set of NAC sequences was clustered by CD-HIT software ([61]; see Materials and Methods) to reduce sequence redundancy, resulting in 2016 non-redundant sequences used for the phylogenetic analyses. The phylogenetic tree was arranged into six major clusters, as shown in the Fig 3.

There were some conserved residues in the NAC domain, suggesting a vital role in their structure due to their prevalence through distinct lineages, as in the case of the GxxFxP residues into A-motif, the W residue into the C-motif, and the WxMHEY signature in the D-motif. The importance of these residues should be regarded in future studies to unravel the origin and evolution of the NAC domain in plant lineage.

In the phylogenetic analysis, sequences from monocots and eudicots tend to cluster in the same clades, suggesting a lineage-specific expansion after divergence from their common ancestor (see S4 Fig). Arabidopsis sequences were used as references for the phylogenomic analysis of the NAC family because of the experimental support of this model species. The NAC group I (see Fig 3) contains proteins involved in controlling cell wall composition, biosynthesis, and xylem development [94]. According to our results, Group I is proposed as the basal NAC group, given the essential angiosperm-specific innovations in water conduction controlled by a Vascular NAC Domain (VND) subgroup of NAC family. Previous authors [54] determined that NAC transcription factors may have contributed to the evolution of both water conducting and cell supporting during the adaptation of plants to land. In Group I, *P*. *patens'* 12 sequences were clustered, with only one sequence with a TMM, and this group contains 8 grapevine and 12 rice sequences. We determined that OG Vvin21-Athal22 (ANAC070, BEARSKIN2, BRN2; S7 Table) is involved in root cap maturation and cell wall modifications. Athal22 contains only one paralogous sequence: Athal35 (ANAC070, BEARSKIN1). All eudicot species share the orthologous sequence BRN2, except for the papaya tree [95]. The OG Vvin27-Athal40 (ANAC043, NST1) has two paralogous sequences, Athal44 (ANAC012, NST3) and Athal46 (ANAC066, NST2), which are involved in secondary wall synthesis, and have redundancy with the VND proteins in Arabidopsis [96]. We could only detect one NST in papaya tree, cucumber, and cacao; two sequences for rice, soybean and tomato; three sequences in barley, Arabidopsis, cassava and Poplar tree; four sequences for maize; and five sequences for banana tree.

We determined the OG Vvin25-Athal34 (ANAC037, VND1) has two paralogous sequences, Athal30 (ANAC076, VND2) and Athal45 (ANAC105, VND3). The other VND sequences of Arabidopsis are in the OGs Vvin16-Athal28 (VND4, ANAC007), which has only one paralogous sequence Athal33 (VND5/ANAC026), and Vvin28-Athal42 (VND7, ANAC030). Other researchers [54] found eight NAC sequences in *P*. *patens* with similarity to the VND/NST/SND proteins, and they named the sequences PpVNS [VND-, NST/SND-, SMB (SOMBRERO)-related protein]. These sequences have a conserved transcriptional regulation and cellular function between moss and Arabidopsis water-conducting cells. Ppat23 (PpVNS7) can regulate many putative orthologous to the VND/NST/SND direct targets, such as *MYB46/83/103*; transcriptional activators for secondary wall formation; *CesA*, a cellulose synthase subunit; *IRX7/FRA8*, a glucuronoxylan biosynthesis protein; *4CL*, a lignin biosynthesis, and *XCP*, a papain-like cysteine peptidase. According to our analysis, the Ppat23 (PpVNS7) sequence of *P*. *patens*, which was specific to eudicot plants, is orthologous to the Vvin25 protein, which in turn is orthologous to the VND1 sequence in Arabidopsis. The OG PpVNS7-Vvin25 appears to be the basal group of the VND-related proteins, suggesting the expansion of this group through concerted evolution. We hypothesize that Vvin25, Vvin16 and Vvin28 (group I-A) were the principal NAC VND sequences that expanded through angiosperm lineage. We found three VND genes for papaya tree, tomato, and cucumber, seven genes for Arabidopsis, and 15 VND genes for Poplar. In previous work, it was determined that only two VND genes are present in *P*. *abies* (Gymnosperm) compared with seven genes in Arabidopsis [78].

The NAC group II (see Fig 3) hosts 8 *P*. *patens* sequences, 9 grapevine sequences and 24 rice sequences. The OG Vvin6-Athal2 (ANAC100, an ethylene responsive gene; see S7 Table), contains the paralogous sequence Athal6 (ANAC080). ANAC080 has a characterized orthologous RhNAC100 in *Rosa hybrid*, and it is involved in cell expansion in flower petals via ethylene network [97]. The Vvin38 protein is orthologous to Athal62 (ANAC22), which is involved in auxin signaling promoting lateral root development [98]. This group II hosts the CUC1-3 (Cup-shape cotyledon) proteins that are involved in shoot apical meristem (SAM) formation and cotyledon separation during embryogenesis in Arabidopsis [35, 99]. The OG Vvin10-Athal16 (CUC2, ANAC098) has the paralogous sequence Athal27 (CUC1, ANAC054) in Arabidopsis. This protein has orthologous sequences in all analyzed eudicot plants, except in the Medicago plant. The OG Vvin30-Athal39 (CUC3, ANAC031) contains other characterized CUC3 proteins in the model species *A*. *thaliana*. Within group II functions, there are processes of specific development among the different organs, such as leaf, root, and floral development, and other processes are probably involved in ethylene-auxin pathways.

For the NAC group III (see Fig 3), 5 *P*. *patens* sequences were identified, while 17 and 12 sequences were found for grapevine and rice respectivelye, respectively. Furthermore, 142 of the 164 sequences containing TMMs were clustered into this group. Therefore, we named it the TMM Group. The TMM group possesses all the NTL reported sequences from Arabidopsis (NTL1-NTL14). These TMM motif sequences are regulated by proteolytic cleavage of the anchor by intra-membrane proteases and are often mediated by environmental factors and stress signaling, such as high salinity (NTL4, NTL5, NTL6 and NTL8), heat (NTL1 and NTL11), cold (NTL2, NTL3, NTL6, NTL7 and NTL9), drought (NTL2, NTL6 and NTL9), H_2_O_2_ (NTL4, NTL5, NTL7 and NTL9), ABA (NTL6-regulating PR genes), cell division (NTL12), mitochondrial retrograde signaling mediating primary stress responses (NTL7), and gibberellic acid (NTL8) [23, 100, 101, 102, 103, 104]. This strategy ensures rapid transcriptional responses to environmental fluctuations, and it would be an efficient way to maximize plant survival under adverse conditions [22]. We determined five sequences in grapevine (Vvin35, Vvin36, Vvin41, Vvin46, and Vvin48) that host the basal NTL sequences in Arabidopsis (see S7 Table), which most probably expanded differently through each plant lineage by different duplication mechanisms to adapt to the needs of each open niche in the environment. The Populus and apple tree are the two species with the highest number of gene expansions into the group of eudicot plants analyzed, with 34 and 26 NTL sequences, unlike grapevine, papaya tree, common bean, and *M*. *truncatul*a that contain a lower number of NTL sequences in their genomes.

The NAC group IV (see Fig 3) hosts a *P*. *patens* sequence (Ppat27), 15 grapevine sequences and 13 rice sequences. The Ppat27 sequence belongs to BOG3, which is shared with the Vvin34 sequence of grapevine and Osat44 from rice. The Vvin34 sequence is orthologous to Athal59 (ANAC035, LOV1; see S7 Table). LOV1 controls the flowering time by negatively regulating CONSTANS (CO), a floral promoter in the circadian light pathway and cold response [105]. Another OG hosted in this clade is Vvin31-Athal48 (ANAC009, FEZ), which controls the reorientation and timing of cell division in a subset of stem cells [106]. The Athal63 gene from Arabidopsis (ANAC042, JUNGBRUNNEN1/JUB1) is orthologous to the Vvin42 sequence in grapevines. JUB1 is a hydrogen peroxide (H_2_O_2_)-induced NAC transcription factor that plays a central role in the longevity regulation of Arabidopsis, and its over-expression delays senescence and enhances tolerance to various abiotic stresses [33].

The NAC group V (see Fig 3) hosts 8 *P*. *patens* sequences, 14 grapevine sequences, and 16 rice sequences. The OG Vvin3-Athal4 (ANAC056, NARS1, ORS1; see S7 Table) contains a Jasmonic Acid (JA) regulatory protein [107]. The OG Vvin59-Athal93 (ANAC104, XND1) contains a characterized Arabidopsis protein that acts as a negative regulator of ligno-cellulose synthesis and programmed cell death in the xylem. A previous [108] report found four putative orthologs to XND1 in Poplar's genome, suggesting the presence of a more complicated xylem in Poplar versus Arabidopsis. We found the same four XND-related sequences in Poplar contained in the OG Vvin59-Ptric191. This sequence also contained three paralogous genes: Ptric195, Ptric198 and Ptric199. Grapevine, papaya tree, *E*. *salsugineum*, *Ricinus*, cassava, and cucumber plant all have only one XND-related sequence. The OG Vvin2-Athal1 (ANAC029, AtNAP) is responsible for controlling leaf senescence and is involved in programmed cell death (PCD) in Arabidopsis [109]. This gene has orthologous members in all the analyzed angiosperms, with several paralogous genes each. The heterologous overexpression of PvNAP (common bean) and OsNAP (rice) was able to restore the Arabidopsis *atnap* null mutant to wild-type, suggesting that NAP may be a universal regulator in plant leaf senescence. Additionally, AtNAP controls stomatal movement and water loss during leaf senescence via the ABA pathway [110]. We defined the OG Vvin12-Athal7 (ANAC072, RD26) as an important abiotic stress-responsive gene from Arabidopsis, which has two paralogous sequences, Athal19 (ANAC019) and Athal18 (ANAC055). All the analyzed eudicot plants, with the exception of *M*. *truncatula*, contain this orthologous sequence, suggesting the importance of this mechanism to cope with abiotic stress. These proteins improve tolerance against several abiotic stresses in plant systems [20, 111]. Athal10 (ANAC002, ATAF1), another Arabidopsis stress responsive gene, negatively regulates several stress related genes as COR47, ERD10, KIN1, RD22 and RD29. The a*taf1* mutants displayed a recovery rate about seven times higher than wild-type plants in a drought response test [40]. This gene is in the OG Vvin7-Athal10 and has orthologous sequences in all the studied plants except the apple tree. Furthermore, the group V contains many others sequences that have been characterized in other species and have improved tolerance against abiotic stresses in tomato proteins Slyc6 (SlNAC1) and Slyc3 (SlNAM1), soybean proteins Gmax24 (GmNAC20) and Gmax109 (GmNAC11), and rice proteins Osat16 (OsNAC5), Osat5 (OsNAC6, SNAC2), and Osat1 (OsNAC10) [112, 113, 114, 115]. According to our results, Group V is proposed as Stress Group of NAC family.

Finally, the NAC group VI contains one *P*. *patens* sequence (belonging to BOG5), 5 grapevine sequences, and 18 rice sequences. This group hosts many species-specific sequences for rice and contains a great abundance of species-specific sequences for the rest of angiosperms, especially Populus, apple tree, Nicotiana, and tomato. These species have experienced various WGDs through their evolutionary history, resulting in the expansion of their gene families. Our results show that each lineage continued expanding after divergence from the common ancestor, resulting in species-specific innovations according to their needs.

The fate of paralogous genes is poorly understood. The prevailing theory predicts that duplicated genes will eventually be lost or mutated. However, several duplications are retained in the genome, probably via new functionalities or sub-functionalities [116]. In Arabidopsis, it has been demonstrated that many genes involved in signal transduction and transcription were preferentially retained after the most recent WGD event [117, 118], suggesting the important roles of TF duplications in plant evolution. Genomic comparisons allow us to transfer genomic knowledge and generate functional hypotheses, moving from acquired experimental information on model species to less-studied taxa. The analysis of orthologous groups in all the other plant species, both monocots and eudicots, thus helped us study the evolutionary dynamics of NACs and to understand shared patterns across the analyzed genomes. This analysis also allowed us to predict each member’s functions using the overall generated knowledge of the model species.

## Conclusions

Comparative genomic analysis and gene functional analysis have shed new light on many aspects of how gene and genome duplicates have contributed to the rapid diversification of the NAC family into the angiosperm lineage by functional innovations such as key pathways, linked to the origin of flowering plants. In this study, we have broadened the number of analyzed species to unravel the origin, evolutionary history, and fate of the expanded NAC family. We observed that the expansion of the NAC family occurred shortly before the origin and rapid radiation of flowering plants. We also observed a gradual increase in the gene number, from the early diverging *P*. *patens*, to more complex trees, such as the Poplar, which wastree consistent with the occurrence of a WGD event in angiosperm history. We have identified 3,187 NAC TFs in 24 green plants and identified the principal sequences from the early diverging *P*. *patens* plant, termed basal sequences, which most likely expanded through the flowering plant lineage by different duplication mechanisms, thus allowing the diversification and adaptation of angiosperms to almost all environments. We found evidence, which had remained unknown before this work, of these five basal sequences in both monocot and eudicot plants. Comparative genomic analysis of the NAC family has opened new possibilities for the systematic functional analysis of new members while providing the basis for further functional research on this vascular plant gene family. Learning how regulatory proteins acquire new molecular functions is essential for developing an understanding of how organisms adapt to new biological niches. The NAC family represents a useful framework in which to address this question. This work is a starting point for characterizing the function of many neglected NAC genes and has provided us with a better understanding of the origin and evolution of the NAC family.

## Supporting Information

S1 Fig HMM LOGO analysis of the NAC domains of 24 plant species. This file contains the HMM NAC domain LOGOS of each of the 24 plant species analyzed.
(PDF)

S2 Fig Comparative analysis of the BOGs and their relative sequences. BOXSHADE analysis of the BOGs and their relative sequences in monocot and eudicot plant species. Plesiomorphic characters are shown in black shadow boxes, principal synapomorphies are shown in red and green letters, and autapomorphies are shown in yellow letters. Similar sequences are colored in gray shadow boxes. The TMM region is shown in blue letters.
(PDF)

S3 Fig Motifs found outside the NAC domain in the five BOGs. Motifs scan of the BOGs and their relative sequences in monocot and eudicot plants. A) BOG1, B) BOG2, C) BOG3, D) BOG4, and E) BOG5. Motifs are shown in gray boxes.
(PDF)

S4 Fig Phylogenetic tree of the NAC proteins in the 24 land plant species. The tree was arranged in six major clades (I-VI) and subclassified in minor groups. Major clades are highlighted in different colors. The ID sequences for moss *P*. *patens* are shown in blue, for *S*. *moellendorffii* in pink, for monocots in red and for eudicots in green.
(PDF)

S5 Fig Phylogenetic tree of the NAC TF proteins in the basal plant lineages. Maximum Likelihood phylogenetic tree of NAC transcription factor proteins. Phylogenetic analysis was carried out with sequences of three basal plant groups: grapevine (Magnoliopsida), rice (Liliopsida), and *P*. *patens* (Bryophyta). The tree was arranged into six major clades and was then subclassified in minor groups. The ID sequences for *P*. *patens*, rice and grapevine are shown in blue, red and green, respectively. Black asterisks indicated the BOGs shared in the three species and are indicated by red and purple dashed lines. Green colored asterisks and dashed lines indicate the OG for the *P*. *patens*-grapevine; the cyan colored asterisks and dashed lines indicate the OG for the *P*. *patens*-rice.
(PDF)

S1 Table Plant species used for the retrieval and analysis of the NAC sequences. ^A^EnsemblPlants, the ^B^Phytozome database V.9.1, and the ^C^ *S*ol Genomics Network were used to collect sequences.
(PDF)

S2 Table List from 24 plants used in this study. This file contains the IDs assigned for the retrieved NAC sequences, the names of the protein annotation files for each species, the amino acid sequences for each NAC protein, and short descriptions and annotations generated in this study.
(XLS)

S3 Table List of NAC sequences detected in *C*. *Papaya*. The sequences were named using the prefix CpNAC and a denoting their order of discovery. Above each column, the CpNAC ID, the Phytozome V9.0. database ID number, the best BLASTP hit in a nr-database (GenBank), and the E-value of the best hit are shown.
(PDF)

S4 Table Complete list of the NAC OG proteins in monocots. NAC OG proteins in the Liliopida Class. *Oryza sativa* NAC sequences were used as references. Red colored blocks represent orthologous genes. Species-specific duplications of each gene are shown below the colored blocks. Sequences belonging to the basal orthologous groups are numbered and marked with yellow stars.
(PDF)

S5 Table Complete list of NAC OG proteins in eudicots. List of OG proteins from the Class Magnoliopsida using the *Vitis vinifera* sequences as references. Purple colored blocks represent orthologous sequences. Species-specific duplications of each gene are shown below the colored blocks. Sequences belonging to the basal orthologous groups are numbered and marked with yellow stars.
(PDF)

S6 Table Complete list of NAC OGs shared by monocots and eudicots. List of 31 NAC OG proteins in basal angiosperm species. Sequences of rice and grapevine are marked in red and purple boxes, respectively. Sequences with the reciprocal BBH are shown in colored boxes. Paralogous sequences are shown below the colored boxes. The five BOGs are marked with yellow stars.
(PDF)

S7 Table Complete set of NAC OGs identified in this study. This file contains the OGs identified in the monocot lineage, the eudicot lineage, and between the monocot and eudicot lineages, as well as the OGs detected between *P*. *patens* as an outgroup and each representative taxa for the monocot and eudicot lineages. The Arabidopsis sequences were annotated to compare with the eudicot OGs. The basal sequences of *P*. *patens* are shown next to their respective orthologous sequences in both the monocot and eudicot lineages.
(XLS)
