# Supplementary figures and images for: Comparative Genomics of NAC Transcriptional Factors in Angiosperms: Implications for the Adaptation and Diversification of Flowering Plants
Source: PLoS One. 2015 Nov 16;10(11):e0141866. doi: 10.1371/journal.pone.0141866 (PMC4646352; doi:10.1371/journal.pone.0141866)

Figure S1

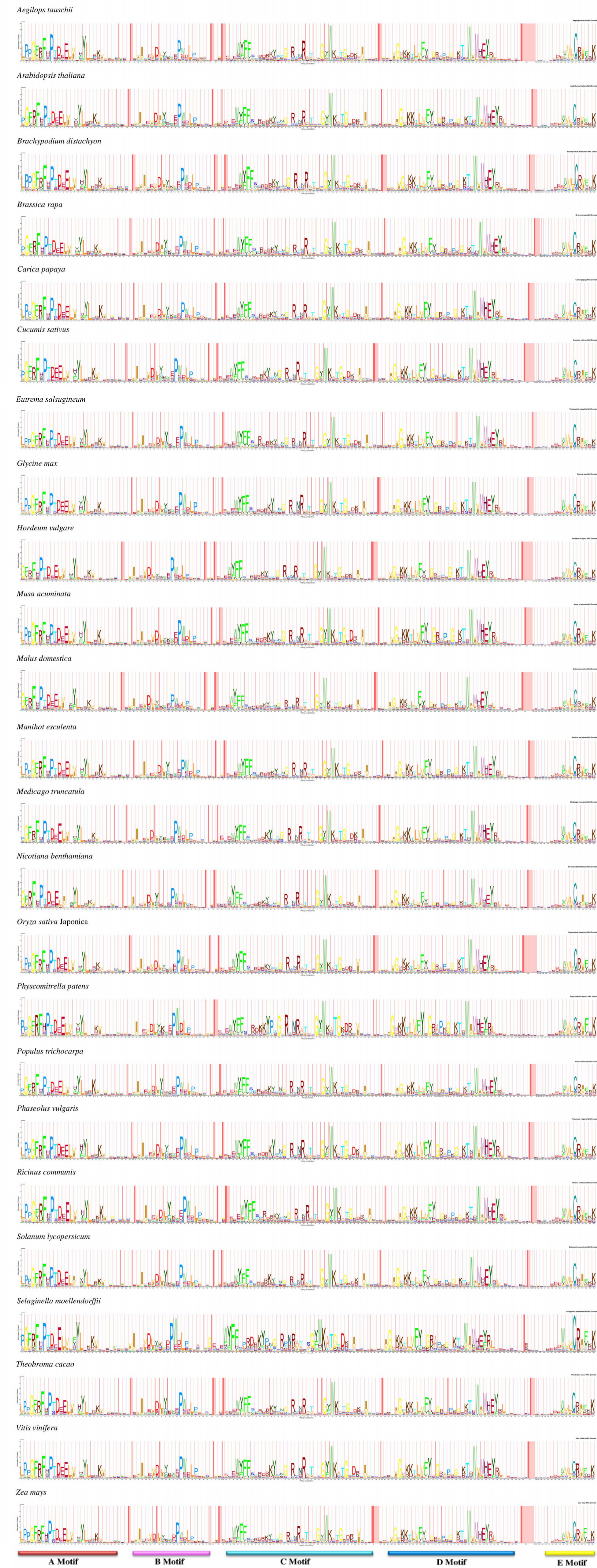

HMM logos of the NAC domain in 24 plant species

Supplement: S1 Fig — This file contains the HMM NAC domain LOGOS of each of the 24 plant species analyzed. (PDF) [file pone.0141866.s001.pdf]
