# Supplementary material for: Comparative Genomics of NAC Transcriptional Factors in Angiosperms: Implications for the Adaptation and Diversification of Flowering Plants
Source: PLoS One. 2015 Nov 16;10(11):e0141866. doi: 10.1371/journal.pone.0141866 (PMC4646352; doi:10.1371/journal.pone.0141866)
Supplement: S2 Fig — BOXSHADE analysis of the BOGs and their relative sequences in monocot and eudicot plant species. Plesiomorphic characters are shown in black shadow boxes, principal synapomorphies are shown in red and green letters, and autapomorphies are shown in yellow letters. Similar sequences are colored in gray shadow boxes. The TMM region is shown in blue letters. (PDF) [file pone.0141866.s002.pdf]

Figure S2

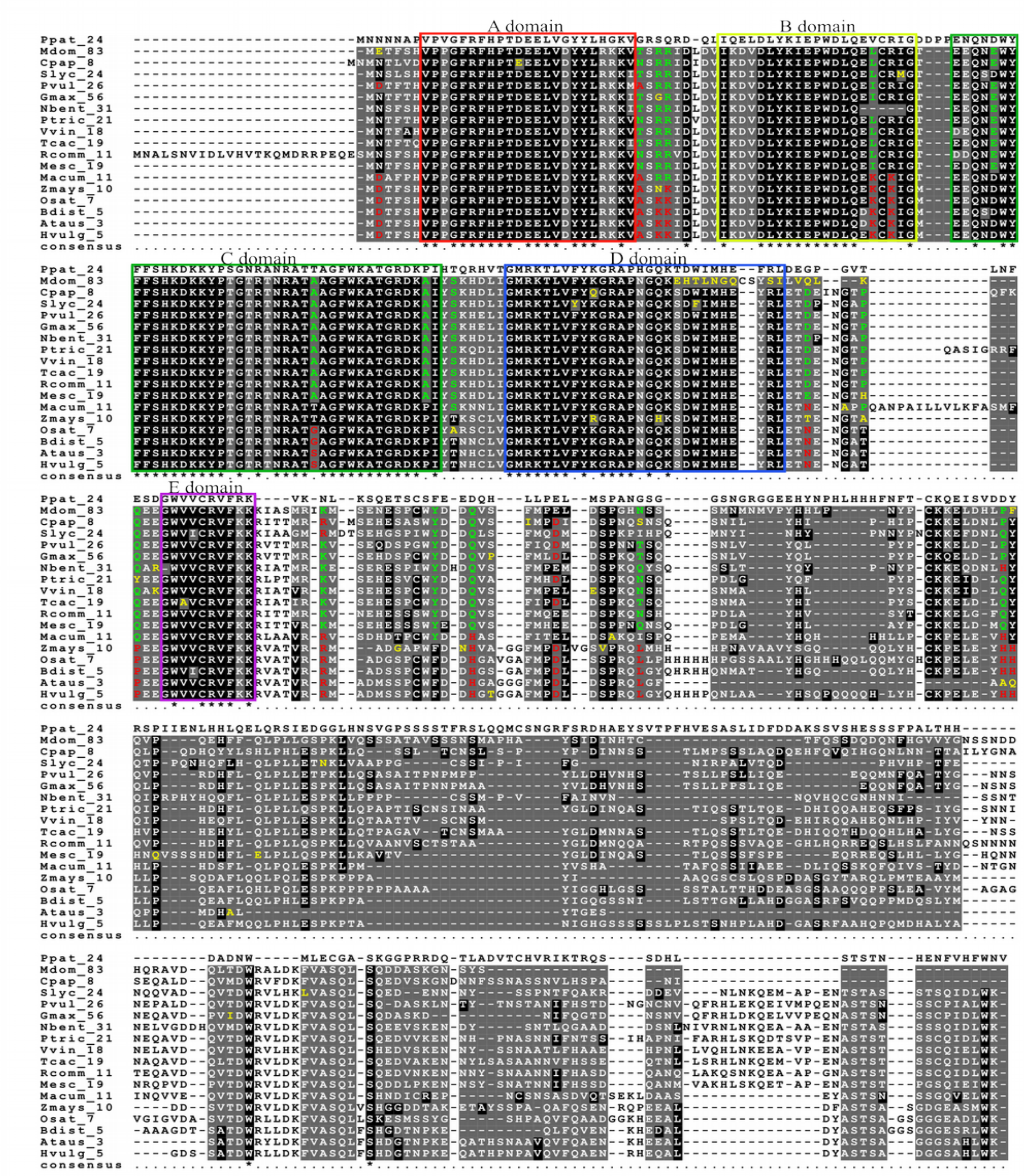

Boxshade analysis of the BOG1 and their relative sequences in monocot and eudicot plant species. Plesiomorphic characters are shown in black shadow boxes, principal synapomorphies are shown in red and green letters, and autoapomorphies are shown in yellow letters. Similar sequences are colored in gray shadow boxes.

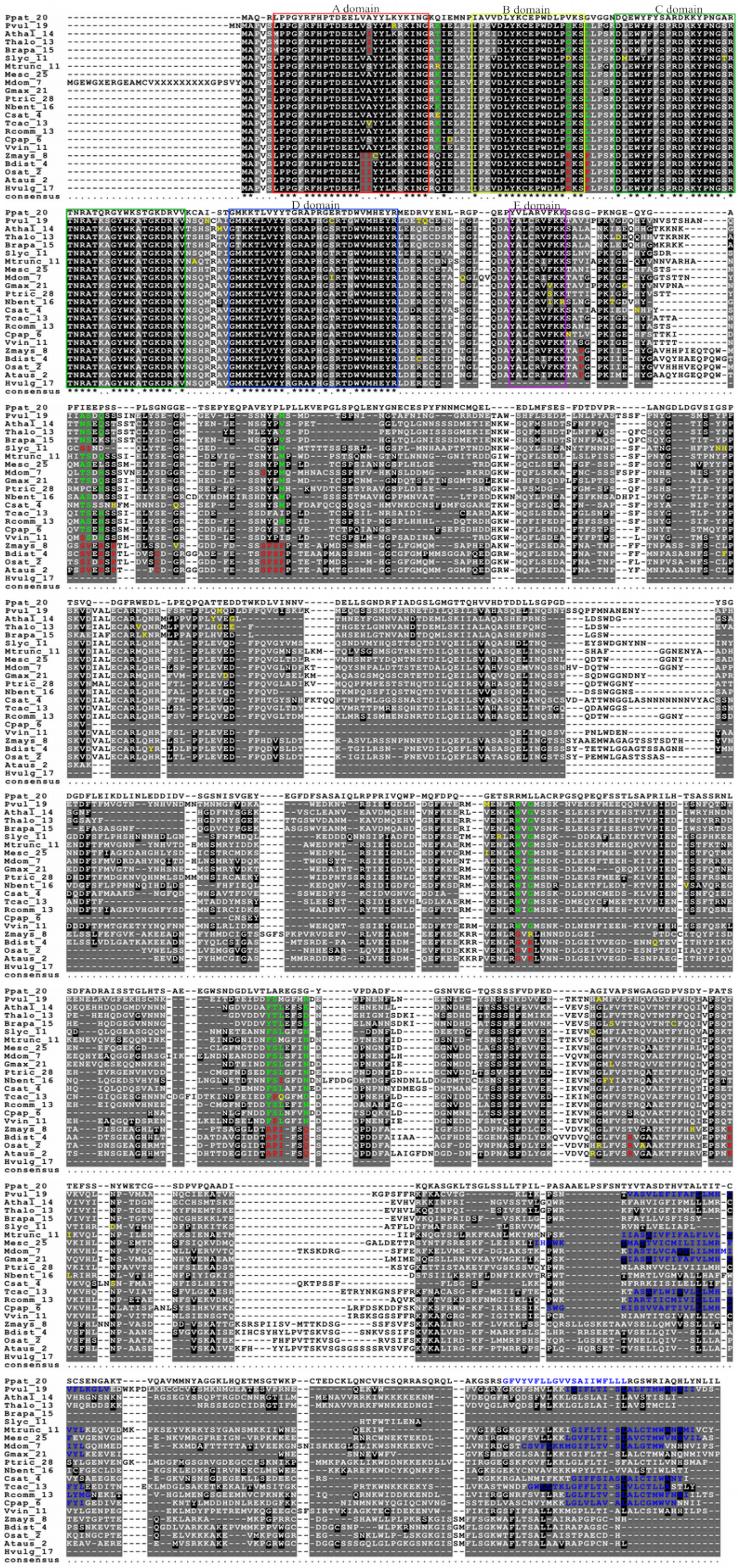

Boxshade analysis of the BOG2 and their relative sequences in monocot and eudicot plant species. Plesiomorphic characters are shown in black shadow boxes, principal synapomorphies are shown in red and green letters, and autoapomorphies are shown in yellow letters. Similar sequences are colored in gray shadow boxes. TMM region are shown in blue letters.

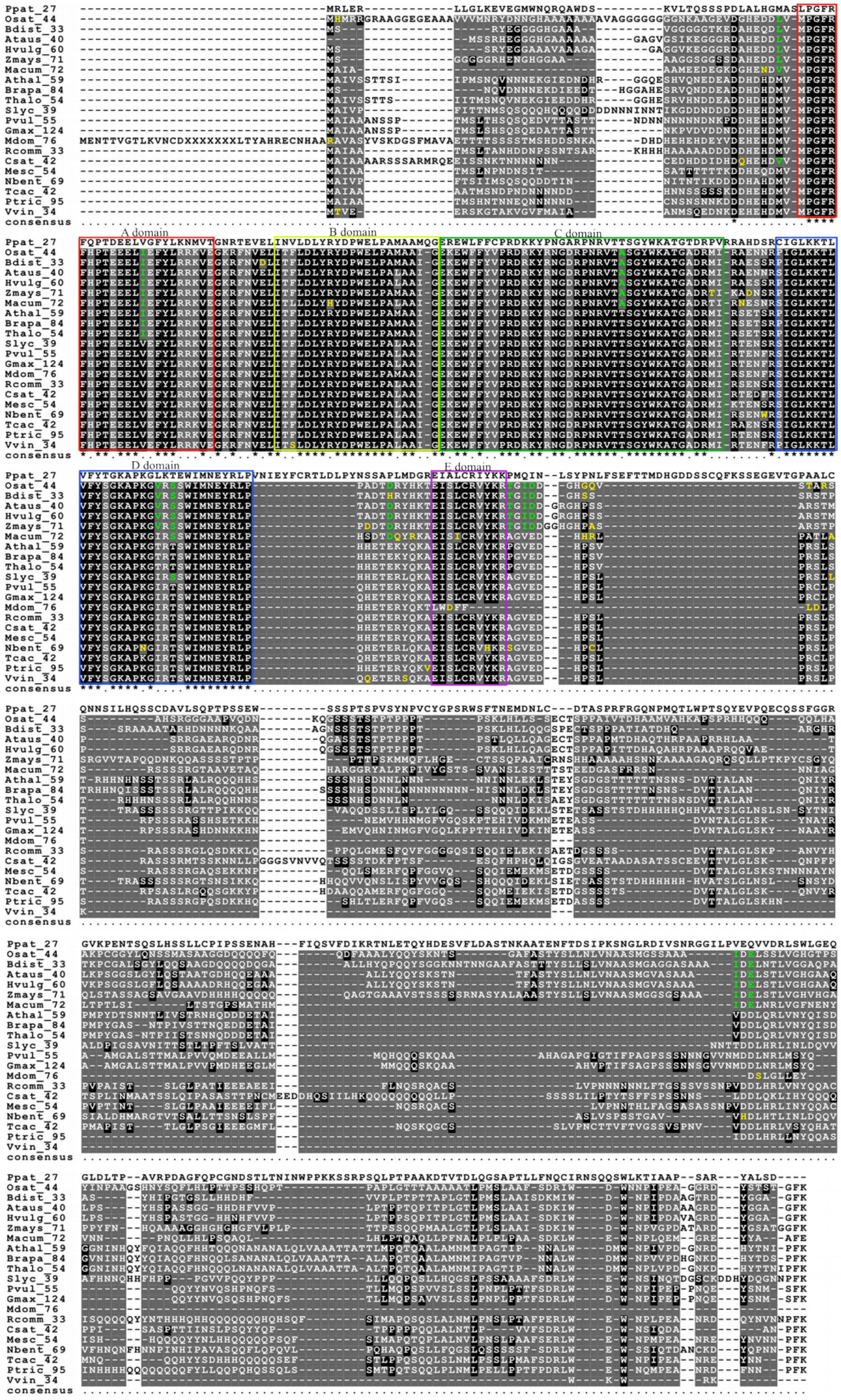

Boxshade analysis of the BOG3 and their relative sequences in monocot and eudicot plant species. Plesiomorphic characters are shown in black shadow boxes, principal synapomorphies are shown in red and green letters, and autoapomorphies are shown in yellow letters. Similar sequences are colored in gray shadow boxes.

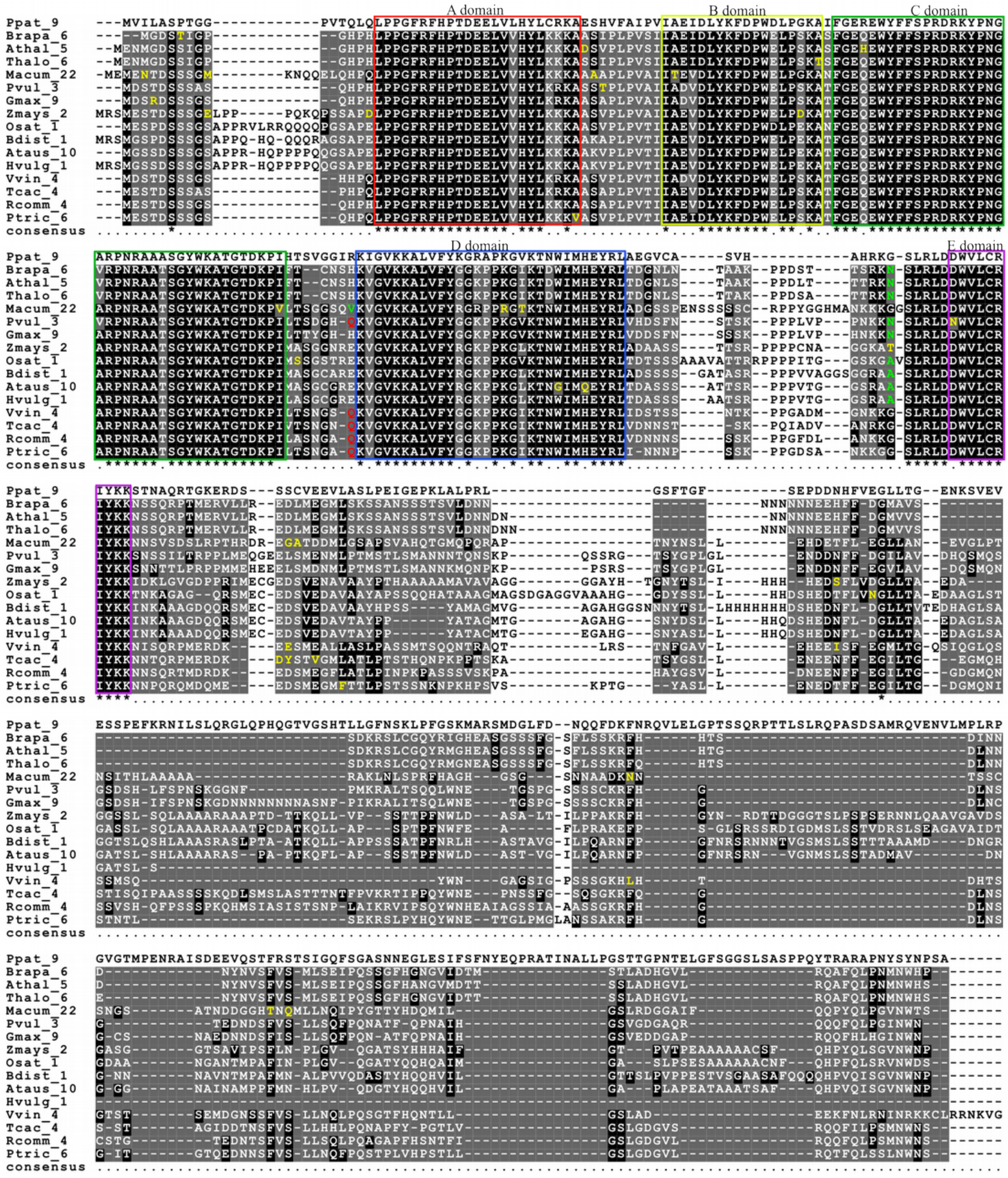

Boxshade analysis of the BOG4 and their relative sequences in monocot and eudicot plant species. Plesiomorphic characters are shown in black shadow boxes, principal synapomorphies are shown in red and green letters, and autoapomorphies are shown in yellow letters. Similar sequences are colored in gray shadow boxes.

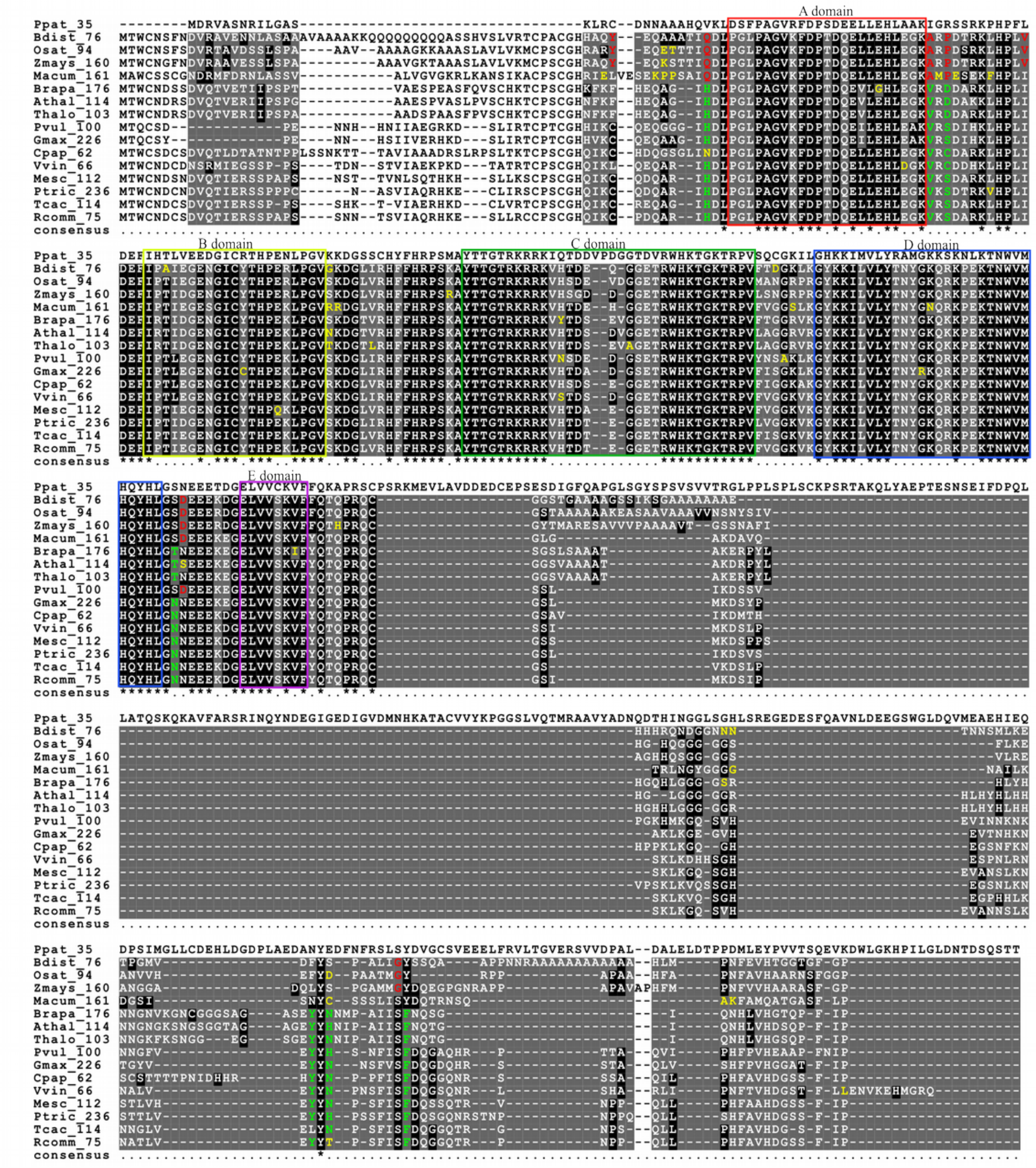

Boxshade analysis of the BOG5 and their relative sequences in monocot and eudicot plant species. Plesiomorphic characters are shown in black shadow boxes, principal synapomorphies are shown in red and green letters, and autoapomorphies are shown in yellow letters. Similar sequences are colored in gray shadow boxes.
