# Supplementary material for: Comparative Genomics of NAC Transcriptional Factors in Angiosperms: Implications for the Adaptation and Diversification of Flowering Plants
Source: PLoS One. 2015 Nov 16;10(11):e0141866. doi: 10.1371/journal.pone.0141866 (PMC4646352; doi:10.1371/journal.pone.0141866)
Supplement: S3 Fig — Motifs scan of the BOGs and their relative sequences in monocot and eudicot plants. A) BOG1, B) BOG2, C) BOG3, D) BOG4, and E) BOG5. Motifs are shown in gray boxes. (PDF) [file pone.0141866.s003.pdf]

Figure S3

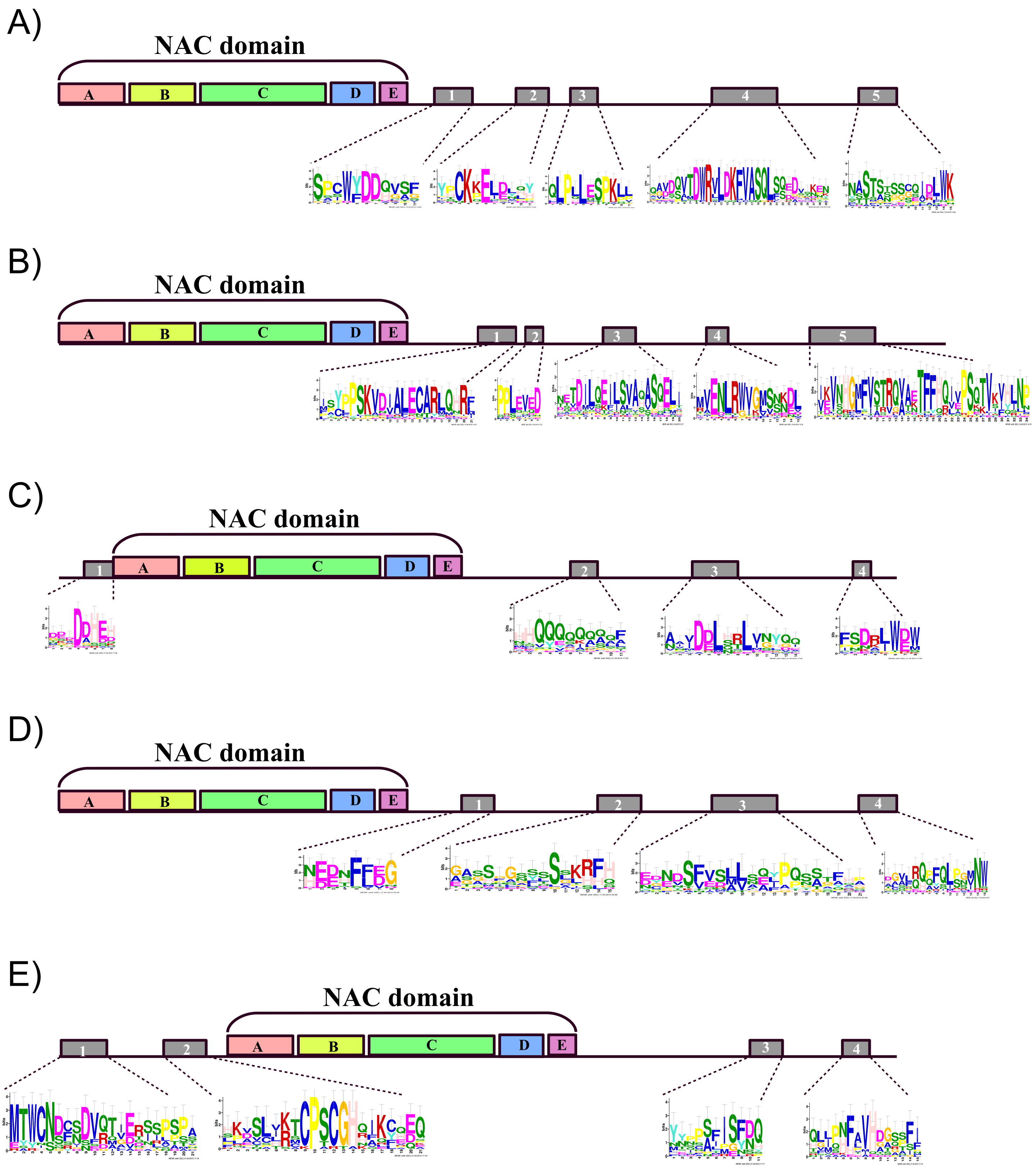

Motifs found outside the NAC domain in the five BOGs. (A) BOG1, (B) BOG2, (C) BOG3, (D) BOG4, and (D) BOG5. Motifs are shown in gray boxes.
