# Supplementary material for: Comparative Genomics of NAC Transcriptional Factors in Angiosperms: Implications for the Adaptation and Diversification of Flowering Plants
Source: PLoS One. 2015 Nov 16;10(11):e0141866. doi: 10.1371/journal.pone.0141866 (PMC4646352; doi:10.1371/journal.pone.0141866)
Supplement: S5 Fig — Maximum Likelihood phylogenetic tree of NAC transcription factor proteins. Phylogenetic analysis was carried out with sequences of three basal plant groups: grapevine (Magnoliopsida), rice (Liliopsida), and P. patens (Bryophyta). The tree was arranged into six major clades and was then subclassified in minor groups. The ID sequences for P. patens, rice and grapevine are shown in blue, red and green, respectively. Black asterisks indicated the BOGs shared in the three species and are indicated by red and purple dashed lines. Green colored asterisks and dashed lines indicate the OG for the P. patens-grapevine; the cyan colored asterisks and dashed lines indicate the OG for the P. patens-rice. (PDF) [file pone.0141866.s005.pdf]

Figure S5

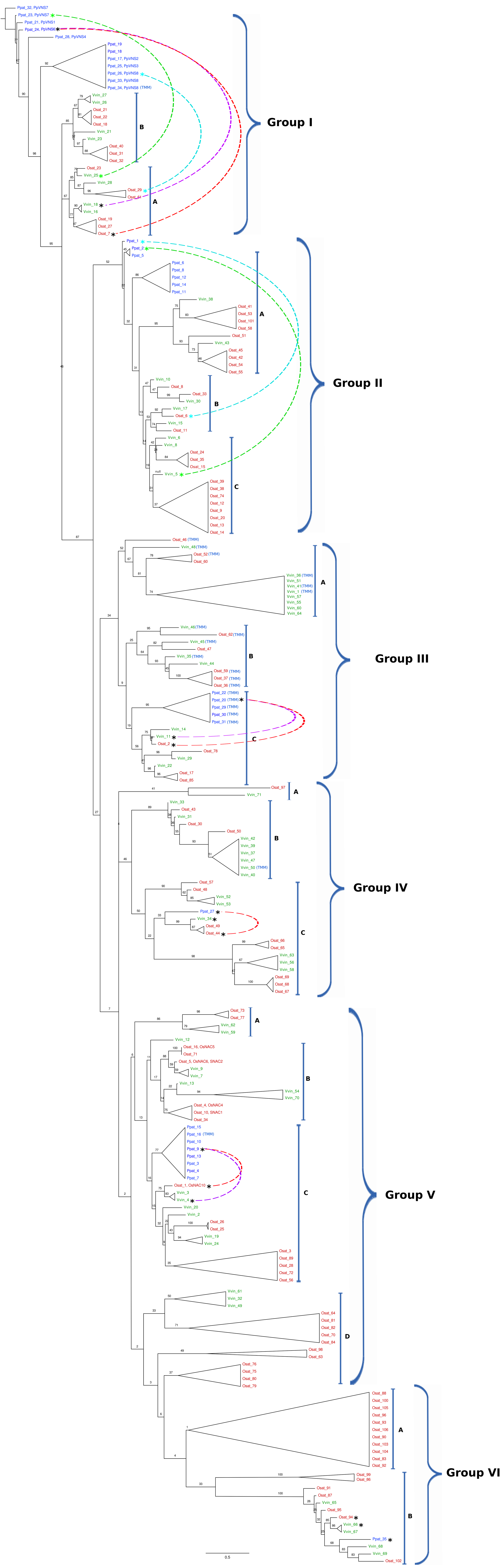

Maximun Likelihood phylogenetic tree of NAC transcription factors proteins in basal plant lineages. Sequences ID for *P. patens*, rice and grapevine are shown in blue, red and green respectively. Black asterisks indicated the BOGs shared in the three species and are indicated by red and purple dashed lines. Green colored dashed lines indicated OG for *P. patens*-grapevine, and cian colored dashed lines indicated OG for *P. patens*-rice.
