# Supplementary material for: Comparative Genomics of NAC Transcriptional Factors in Angiosperms: Implications for the Adaptation and Diversification of Flowering Plants
Source: PLoS One. 2015 Nov 16;10(11):e0141866. doi: 10.1371/journal.pone.0141866 (PMC4646352; doi:10.1371/journal.pone.0141866)
Supplement: S1 Table — AEnsemblPlants, the BPhytozome database V.9.1, and the C Sol Genomics Network were used to collect sequences. (PDF) [file pone.0141866.s006.pdf]

Table S1. Plant species used for the retrieval and analysis of the NAC sequences

Aegilops tauschii BGI[A]	[1]	*Musa acuminata* DH-Pahang CIRAD[A]	[13]
Arabidopsis thaliana TAIR10[B]	[2]	*Nicotiana benthamiana* v0.4.4[C]	[14]
Brachypodium distachyon v2.1[B]	[3]	*Oryza sativa* var. japonica RAP-DB[A]	[15]
Brassica rapa FPsc v1.3[B]	[4]	*Physcomitrella patens* v3.0[B]	[16]
Carica papaya ASGPBv0.4[B]	[5]	*Populus trichocarpa* v3.0[B]	[17]
Cucumis sativus v1.0[B]	[6]	*Phaseolus vulgaris* v1.0[B]	[18]
Eutrema salsugineum v1.0[B]	[7]	*Ricinus communis* v0.1[B]	[19]
Glycina max Wm82.a2.v1[B]	[8]	*Selaginella moellendorffii* v1.0[B]	[20]
Hordeum vulgare IBSC[A]	[9]	*Solanum lycopersicum* iTAG2.3[B]	[21]
Malus domestica v1.0[B]	[10]	*Theobroma cacao* v1.1[B]	[22]
Manihot esculenta v4.1[B]	[11]	*Vitis vinifera* Genoscope.12x[B]	[23]
Medicago truncatula Mt4.0v1[B]	[12]	*Zea mays* 6a[B]	[24]

1. Jia J, Zhao S, Kong X, Li Y, Zhao G, He W, et al. Aegilops tauschii draft genome sequence reveals a gene repertoire for wheat adaptation. Nature. 2013; 496(7443):91-5. doi: 10.1038/nature12028 PMID: 23535592

2. Arabidopsis Genome Initiative. Analysis of the genome sequence of the flowering plant Arabidopsis thaliana. Nature. 2000; 408(6814):796-815. PMID: 11130711

3. International Brachypodium Initiative. Genome sequencing and analysis of the model grass Brachypodium distachyon. Nature. 2010; 463(7282):763-768. doi: 10.1038/nature08747 PMID: 20148030

4. Johnston JS, Pepper AE, Hall AE, Chen ZJ, Hodnett G, Drabek J, et al. Evolution of Genome Size in Brassicaceae. Ann Bot. 2005; 95(1):229-35. PMID: 15596470

5. Ming R, Hou S, Feng Y, Yu Q, Dionne-Laporte A, Saw JH, et al. The draft genome of the transgenic tropical fruit tree papaya (Carica papaya Linnaeus). Nature. 2008; 452(7190):991-996. doi: 10.1038/nature06856 PMID: 18432245

6. Huang S, Li R, Zhang Z, Li L, Gu X, Fan W, et al. The genome of the cucumber, Cucumis sativus L. Nat Genet. 2009; 41(12):1275-81. doi: 10.1038/ng.475. PMID: 19881527

7. Yang R, Jarvis DE, Chen H, Beilstein MA, Grimwood J, Jenkins J, et al. The reference genome of the halophytic plant Eutrema salsugineum. Front Pant Sci. 2013; 4(46). doi: 10.3389/fpls.2013.00046 PMID: 23518688

8. Schmutz J, Cannon SB, Schlueter J, Ma J, Mitros T, Nelson W, et al. Genome sequence of the Palaeopolyploid soybean. Nature. 2010; 463(7278):178-183. doi: 10.1038/nature08670 PMID: 20075913

9. International Barley Genome Sequencing Consortium. A physical, genetic and functional sequence assembly of the barley genome. Nature. 2012; 491(7426):711-716. doi: 10.1038/nature11543 PMID: 23075845

10. Velasco R, Zharkikh A, Affourtit J, Dhingra A, Cestaro A, Kalyanaraman A, et al. The genome of the domesticated apple (Malus × domestica Borkh.). Nat genet. 2010; 42(10):833-839. doi: 10.1038/ng.654 PMID: 20802477

11. Prochnik S, Marri PR, Desany B, Rabinowicz PD, Kodira C, Mohiuddin M, et al. The Cassava Genome: Current Progress, Future Directions. Trop Plant Biol. 2012; 5(1):88-94. PMID: 22523606

12. Young ND, Debelle F, Oldroyd GED, Geurts R, Cannon SB, Udvardi MK, et al. The Medicago genome provides insight into the evolution of rhizobial symbioses. Nature. 2011; 480(7378):520-524. doi: 10.1038/nature10625 PMID: 22089132

13. D'Hont A, Denoeud F, Aury JM, Baurens FC, Carreel F, Garsmeur O, et al. The banana (Musa acuminata) genome and the evolution of monocotyledonous plants. Nature. 2012; 488:213-217. doi: 10.1038/nature11241

14. Bombarely A, Rosli HG, Vrebalov J, Moffett P, Mueller LA, Martin GB. A Draft Genome Sequence of Nicotiana benthamiana to Enhance Molecular Plant-Microbe Biology Research, MPMI. 2012; 25(12): 1523-1530. doi: http://dx.doi.org/10.1094/MPMI-06-12-0148-TA

15. International Rice Genome Sequencing Project. The map-based sequence of the rice genome. Nature. 2005; 436(7052):793-800. PMID: 16100779

16. Rensing SA, Lang D, Zimmer AD, Terry A, Salamov A, Shapiro H, et al. The Physcomitrella genome reveals evolutionary insights into the conquest of land by plants. Science. 2008; 319(5859):64-9. PMID: 18079367

17. Tuskan GA, Difazio S, Jansson S, Bohlmann J, Grigoriev I, Hellsten U, et al. The genome of black cottonwood, Populus trichocarpa (Torr. & Gray). Science. 2006; 313(5793):1596-1604. PMID: 16973872

18. Schmutz J, McClean PE, Mamidi S, Wu GA, Cannon SB, Grimwood J, et al. A reference genome for common bean and genome-wide analysis of dual domestications. Nature genetics. 2014; 46(7):707-713. doi: 10.1038/ng.3008 PMID: 24908249

19. Chan AP, Crabtree J, Zhao Q, Lorenzi H, Orvis J, Puiu D. et al. Draft genome sequence of the oilseed species Ricinus communis. Nat Biotechnol. 2010; 28:951-956. doi: 10.1038/nbt.1674

20. Wang W, Tanurdzic M, Luo M, Sisneros N, Kim HR, Weng JK, et al. Construction of a bacterial artificial chromosome library from the spikemoss Selaginella moellendorffii: a new resource for plant comparative genomics. BMC Plant Biology. 2005; 5(10). PMID: 15955246

21. Tomato Genome Consortium. The tomato genome sequence provides insights into fleshy fruit evolution. Nature. 2012; 485(7400):635-641. doi: 10.1038/nature11119 PMID: 22660326

22. Argout X, Salse J, Aury JM, Guiltinan MJ, Droc G, Gouzy J, et al. The genome of Theobroma cacao, Nature genetics. 2011: 43(2):101-108. doi: 10.1038/ng.736

23. Velasco R, Zharkikh A, Troggio M, Cartwright DA, Cestaro A, Pruss D, et al. A high quality draft consensus sequence of the genome of a heterozygous grapevine variety. PloS one. 2007; 2(12):e1326. PMID: 18094749

24. Schnable PS, Ware D, Fulton RS, Stein JC, Wei F, Pasternak S, et al. The B73 maize genome: complexity, diversity, and dynamics. Science. 2009; 326(5956):1112-1115. Erratum in: Science. 2012; 337(6098):1040. doi: 110.1126/science.1178534 PMID: 19965430

[A]http://plants.ensembl.org/index.html, EMBL-EBI

[B]Goodstein DM, Shu S, Howson R, Neupane R, Hayes RD, Fazo J, et al. Phytozome: a comparative platform for green plant genomics. Nucleic Acids Res. 2012; 40 (Database issue):D1178-86. doi: 10.1093/nar/gkr944 PMID: 22110026

[C]Fernandez-Pozo N, Menda N, Edwards JD, Saha S, Tecle IY, Strickler SR, et al. The Sol Genomics Network (SGN)-from genotype to phenotype to breeding. Nucl. Acids Res. 2014; doi: 10.1093/nar/gku1195
