# Supplementary material for: Comparative Genomics of NAC Transcriptional Factors in Angiosperms: Implications for the Adaptation and Diversification of Flowering Plants
Source: PLoS One. 2015 Nov 16;10(11):e0141866. doi: 10.1371/journal.pone.0141866 (PMC4646352; doi:10.1371/journal.pone.0141866)
Supplement: S3 Table — The sequences were named using the prefix CpNAC and a denoting their order of discovery. Above each column, the CpNAC ID, the Phytozome V9.0. database ID number, the best BLASTP hit in a nr-database (GenBank), and the E-value of the best hit are shown. (PDF) [file pone.0141866.s008.pdf]

Table S3. List of NAC sequencenes detected in *C. papaya*.

Name	Contig #	Best BLAST and GenBank accession no.	E value
**CpNAC1**	Supercontig_1	NAC domain protein, IPR003441 [Theobroma cacao] XP_007046037.1	8E-101
**CpNAC2**	Supercontig_80	NAC domain-containing protein, putative [Ricinus communis] XP_002532683.1	8E-175
**CpNAC3**	Supercontig_111	NAC domain-containing protein 21/22, putative [Ricinus communis] XP_002514736.1	7E-175
**CpNAC4**	Supercontig_80	NAC domain class transcription factor isoform 1 [Theobroma cacao] XP_007021328.1	0
**CpNAC5**	Supercontig_64	NAC domain protein, IPR003441 [Theobroma cacao] XP_007019132.1	1e-163
**CpNAC6**	Supercontig_178	Unnamed protein product [Vitis vinifera] CBI27341.3	2E-145
**CpNAC7**	Supercontig_27	Transcription factor, putative [Ricinus communis] XP_002519008.1	5E-158
**CpNAC8**	Supercontig_3	PREDICTED: NAC domain-containing protein 7-like [Vitis vinifera] XP_002264986.2	2E-163
**CpNAC9**	Supercontig_594	NAC domain-containing protein 21/22, putative [Ricinus communis] XP_002525465.1	9E-141
**CpNAC10**	Supercontig_131	TPA: TPA_inf: CUP-SHAPED COTYLEDON 2 [Carica papaya] DAA34939.1	0
**CpNAC11**	Supercontig_244	Transcription factor, putative [Ricinus communis] XP_002527980.1	5E-172
**CpNAC12**	Supercontig_14	hypothetical protein CICLE_v10012093mg [Citrus clementina] XP_006431201.1	4E-172
**CpNAC13**	Supercontig_29	Transcription factor [Populus trichocarpa] BAK14360.1	3E-159
**CpNAC14**	Supercontig_37	NAM-like protein [Bruguiera gymnorhiza] BAG15877.1	3E-145
**CpNAC15**	Supercontig_435	PREDICTED: uncharacterized protein LOC100253566 [Vitis vinifera] XP_002282243.2	4E-097
**CpNAC16**	Supercontig_118	NAC domain protein, IPR003441 [Populus trichocarpa] XP_002316163.1	2E-151
**CpNAC17**	Contig_29561	Transcription factor, putative [Ricinus communis] XP_002525485.1	2E-147
**CpNAC18**	Contig_33298	PREDICTED: uncharacterized protein LOC100248298 [Vitis vinifera] XP_002275457.1	9E-116
**CpNAC19**	Contig_30558	NAC domain-containing protein, putative [Ricinus communis] XP_002526818.1	3E-145
**CpNAC20**	Supercontig_3	NAC domain protein, IPR003441 [Populus trichocarpa] XP_002321475.1	6E-144
**CpNAC21**	Supercontig_38	NAC domain protein, IPR003441 [Populus trichocarpa] XP_002316156.1	5E-118
**CpNAC22**	Supercontig_37	NAC domain-containing protein, putative [Ricinus communis] XP_002518924.1	4E-164
**CpNAC23**	Supercontig_286	TPA: TPA_inf: CUP-SHAPED COTYLEDON 3 [Carica papaya] DAA34940.1	0
**CpNAC24**	Supercontig_6	NAC domain protein, IPR003441 [Populus trichocarpa] XP_002310297.1	7E-104
**CpNAC25**	Supercontig_90	PREDICTED: NAC domain-containing protein 68-like [Vitis vinifera] XP_002280894.2	1E-121
**CpNAC26**	Supercontig_99	NAC domain protein, IPR003441 [Populus trichocarpa] XP_002304662.1	2E-105
**CpNAC27**	Supercontig_37	NAC domain protein, IPR003441 [Populus trichocarpa] XP_002300524.1	9E-151
**CpNAC28**	Supercontig_10	Transcriptional factor NAC35 [Glycine max] NP_001235901.1	6E-120
**CpNAC29**	Supercontig_11	NAC domain protein, IPR003441 [Populus trichocarpa] XP_002325069.1	6E-056
**CpNAC30**	Supercontig_165	NAC domain protein, IPR003441 [Populus trichocarpa] XP_002307652.1	3E-123
**CpNAC31**	Supercontig_42	Hypothetical protein ARALYDRAFT_487745 [Arabidopsis lyrata] XP_002873395.1	2E-102
**CpNAC32**	Supercontig_128	PREDICTED: protein FEZ-like [Vitis vinifera] XP_002270334.1	6E-143
**CpNAC33**	Supercontig_26	NAC domain protein, IPR003441 [Populus trichocarpa] XP_002302150.1	5E-155
**CpNAC34**	Supercontig_3	NAC domain protein, IPR003441 [Populus trichocarpa] XP_002321956.1	6E-140
**CpNAC35**	Supercontig_84	NAC domain protein, IPR003441 [Populus trichocarpa] XP_002314078.1	2E-056
**CpNAC36**	Supercontig_1	NAC domain protein, IPR003441 [Populus trichocarpa] XP_002314078.1	6E-055
**CpNAC37**	Supercontig_94	NAC domain protein, IPR003441 [Populus trichocarpa] XP_002325069.1	1E-049
**CpNAC38**	Supercontig_320	Uncharacterized protein LOC100816770 [Glycine max] NP_001242390.1	7E-123
**CpNAC39**	Supercontig_195	NAC domain protein, IPR003441, putative isoform 1 [Theobroma cacao] XP_007015733.1	8E-140
**CpNAC40**	Supercontig_104	PREDICTED: NAC domain-containing protein 90 [Vitis vinifera] XP_002276192.1	5E-083
**CpNAC41**	Supercontig_57	PREDICTED: NAC domain-containing protein 69-like [Glycine max] XP_003548387.1	4E-049
**CpNAC42**	Supercontig_4	NAC domain protein [Glycine max] NP_001238078.1	9E-075
**CpNAC43**	Supercontig_195	NAC domain protein, IPR003441 [Populus trichocarpa] XP_002298277.1	1E-111
**CpNAC44**	Supercontig_67	NAC domain protein, IPR003441 [Populus trichocarpa] XP_002310103.1	1E-103
**CpNAC45**	Supercontig_63	NAC domain protein, IPR003441 isoform 1 [Theobroma cacao] XP_007043034.1	1E-091
**CpNAC46**	Supercontig_14	PREDICTED: protein FEZ-like [Pyrus x bretschneideri] XP_009376262.1	3E-088
**CpNAC47**	Supercontig_70	Transcription factor, putative [Ricinus communis] XP_002512886.1	3E-053
**CpNAC48**	Supercontig_1	hypothetical protein CISIN_1g046940mg [Citrus sinensis] KDO81808.1	9E-023
**CpNAC49**	Supercontig_53	PREDICTED: NAC domain-containing protein 4-like isoform X1 [Tarenaya hassleriana] XP_010543579	3E-050
**CpNAC50**	Supercontig_42	hypothetical protein CICLE_v10003379mg [Citrus clementina] XP_006432173.1	6E-053
**CpNAC51**	Supercontig_94	NAC transcription factor 058 [Jatropha curcas] AGL39714.1	9E-046
**CpNAC52**	Supercontig_113	Uncharacterized protein TCM_043185 [Theobroma cacao] XP_007009879.1	6E-030
**CpNAC53**	Supercontig_202	PREDICTED: NAC domain-containing protein 45-like isoform X1 [Populus euphratica] XP_011003272	4E-038
**CpNAC54**	Supercontig_113	Uncharacterized protein TCM_043185 [Theobroma cacao] XP_007009879.1	4E-023
**CpNAC55**	Supercontig_33	NAC transcription factor 060 [Jatropha curcas] AGL39716.1	5E-018
**CpNAC56**	Supercontig_33	NAC transcription factor 060 [Jatropha curcas] AGL39716.1	7E-015
**CpNAC57**	Supercontig_4056	hypothetical protein AALP_AA1G056200 [Arabis alpina] KFK42926.1	1E-013
**CpNAC58**	Supercontig_192	Uncharacterized protein TCM_025221 [Theobroma cacao] XP_007029346.1	6E-022
**CpNAC59**	Supercontig_78	PREDICTED: NAC domain-containing protein 8 isoform X3 [Vitis vinifera] XP_010656246.1	0
**CpNAC60**	Supercontig_17	Protein CUP-SHAPED COTYLEDON 3 [Morus notabilis] XP_010094111.1	7E-017
**CpNAC61**	Supercontig_566	PREDICTED: protein FEZ-like [Glycine max] XP_003528722.1	3E-034
**CpNAC62**	Supercontig_83	transcription factor, putative [Ricinus communis] XP_002517849.1	2E-161
**CpNAC63**	Supercontig_53	NAC domain protein, IPR003441 isoform 1 [Theobroma cacao] XP_007046313.1	0
**CpNAC64**	Supercontig_2586	NAC domain containing protein 73 [Theobroma cacao] XP_007025530.1	1E-145
**CpNAC65**	Supercontig_80	conserved hypothetical protein [Ricinus communis] XP_002525549.1	3E-031
**CpNAC66**	Supercontig_80	conserved hypothetical protein [Ricinus communis] XP_002525549.1	2E-030
**CpNAC67**	Supercontig_106	hypothetical protein EUGRSUZ_F03962 [Eucalyptus grandis] KCW70813.1	6E-022
**CpNAC68**	Supercontig_80	conserved hypothetical protein [Ricinus communis] XP_002525549.1	7E-030
**CpNAC69**	Supercontig_64	hypothetical protein CICLE_v10001389mg [Citrus clementina] XP_006434932.1	7E-045
**CpNAC70**	Supercontig_80	conserved hypothetical protein [Ricinus communis] XP_002525549.1	1E-032
**CpNAC71**	Supercontig_80	DNA binding protein, putative [Theobroma cacao] XP_007021609.1	7E-077
**CpNAC72**	Supercontig_80	conserved hypothetical protein [Ricinus communis] XP_002525549.1	6E-032
**CpNAC73**	Supercontig_80	conserved hypothetical protein [Ricinus communis] XP_002525549.1	5E-031
**CpNAC74**	Supercontig_12	hypothetical protein JCGZ_21515 [Jatropha curcas] KDP21044.1	2E-043
**CpNAC75**	Supercontig_200	hypothetical protein EUGRSUZ_F03962 [Eucalyptus grandis] KCW70813.1	1E-020
**CpNAC76**	Contig_37010	unnamed protein product [Vitis vinifera] CBI21309.3	8E-016
**CpNAC77**	Supercontig_21	PREDICTED: NAC domain-containing protein 72-like [Fragaria vesca subsp. Vesca] XP_004291091.1	3E-033
