# Supplementary material for: Comparative Genomics of NAC Transcriptional Factors in Angiosperms: Implications for the Adaptation and Diversification of Flowering Plants
Source: PLoS One. 2015 Nov 16;10(11):e0141866. doi: 10.1371/journal.pone.0141866 (PMC4646352; doi:10.1371/journal.pone.0141866)
Supplement: S4 Table — NAC OG proteins in the Liliopida Class. Oryza sativa NAC sequences were used as references. Red colored blocks represent orthologous genes. Species-specific duplications of each gene are shown below the colored blocks. Sequences belonging to the basal orthologous groups are numbered and marked with yellow stars. (PDF) [file pone.0141866.s009.pdf]

**Table S4**

OGs of NAC's proteins in Monocots. Red colored blocks represents orthologous genes. Paralogous genes are shown below the colored blocks. Sequences of rice belonging to the BOG are numbered and marked with yellow stars.

| | | | | | | |
|---|---|---|---|---|---|---|
| 4 | **Osat_1** | **Ataus_10** | **Bdist_1** | **Hvulg_1** | **Zmays_2** | **Macum_22** |
| | | Ataus_8 | Bdist_31 | | Zmays_1 | Macum_13 |
| | | | | | Zmays_124 | Macum_93 |
| | | | | | Zmays_152 | Macum_110 |
| | | | | | | Macum_118 |
| | | | | | | Macum_125 |
| | | | | | | Macum_127 |
| | | | | | | Macum_130 |
| | | | | | | Macum_132 |
| | | | | | | Macum_142 |
| 2 | **Osat_2** | **Ataus_2** | **Bdist_4** | **Hvulg_17** | **Zmays_8** | **Macum_9** |
| | | | Bdist_29 | Hvulg_135 | Zmays_4 | Macum_8 |
| | | | | Hvulg_136 | Zmays_83 | Macum_15 |
| | | | | | | Macum_45 |
| | | | | | | Macum_74 |
| | | | | | | Macum_79 |
| | | | | | | Macum_83 |
| | | | | | | Macum_90 |
| | | | | | | Macum_113 |
| | | | | | | Macum_126 |
| | | | | | | Macum_128 |
| | **Osat_3** | **Ataus_27** | **Bdist_2** | **Hvulg_2** | **Zmays_3** | **Macum_2** |
| | | Ataus_14 | Bdist_15 | | Zmays_6 | Macum_1 |
| | | Ataus_33 | | | Zmays_37 | Macum_3 |
| | | Ataus_55 | | | Zmays_38 | Macum_4 |
| | | Ataus_80 | | | | Macum_5 |
| | | | | | | Macum_7 |
| | | | | | | Macum_19 |
| | **Osat_4** | **Ataus_64** | **Bdist_6** | **Hvulg_15** | **Zmays_19** | **Macum_27** |
| | | Ataus_76 | | Hvulg_46 | Zmays_15 | Macum_12 |
| | | | | Hvulg_48 | Zmays_17 | Macum_17 |
| | | | | | Zmays_22 | Macum_34 |
| | | | | | Zmays_150 | Macum_42 |
| | | | | | | Macum_43 |
| | **Osat_5** | **Ataus_4** | **Bdist_3** | **Hvulg_6** | **Zmays_11** | **Macum_33** |
| | | | Bdist_11 | Hvulg_10 | Zmays_12 | Macum_21 |
| | | | | Hvulg_40 | Zmays_68 | Macum_28 |
| | | | | Hvulg_45 | Zmays_153 | Macum_32 |
| | | | | Hvulg_50 | Zmays_154 | Macum_41 |
| | | | | | | Macum_50 |
| | | | | | | Macum_167 |
| | **Osat_6** | **Ataus_5** | **Bdist_7** | **Hvulg_7** | **Zmays_18** | **Macum_6** |
| | | Ataus_17 | Bdist_65 | Hvulg_29 | Zmays_5 | Macum_10 |
| | | Ataus_23 | | Hvulg_31 | Zmays_7 | Macum_53 |
| | | Ataus_25 | | Hvulg_32 | Zmays_36 | |
| | | | | Hvulg_47 | | |
| | | | | Hvulg_64 | | |

**Table S4** Continued

1

| | | | | | |
|---|---|---|---|---|---|
| **Osat_7** | **Ataus_3** | **Bdist_5** | **Hvulg_5** | **Zmays_10** | **Macum_11** |
| | | Bdist_16 | Hvulg_3 | Zmays_16 | Macum_26 |
| | | | Hvulg_4 | Zmays_23 | Macum_46 |
| | | | Hvulg_22 | | |
| | | | Hvulg_27 | | |
| | | | Hvulg_116 | | |
| **Osat_8** | **Ataus_45** | | **Hvulg_36** | **Zmays_32** | **Macum_14** |
| | | | | Zmays_24 | Macum_24 |
| | | | | Zmays_51 | |
| | | | | Zmays_178 | |
| **Osat_9** | | | | | |
| **Osat_10** | | **Bdist_17** | **Hvulg_16** | **Zmays_27** | |
| | | Bdist_70 | Hvulg_52 | Zmays_29 | |
| | | | | Zmays_33 | |
| | | | | Zmays_43 | |
| | | | | Zmays_77 | |
| | | | | Zmays_88 | |
| | | | | Zmays_148 | |
| | | | | Zmays_149 | |
| **Osat_11** | **Ataus_7** | **Bdist_9** | **Hvulg_8** | **Zmays_9** | **Macum_57** |
| | Ataus_82 | | | | Macum_31 |
| | | | | | Macum_135 |
| **Osat_12** | **Ataus_11** | | | | |
| | Ataus_13 | | | | |
| | Ataus_20 | | | | |
| | Ataus_32 | | | | |
| **Osat_13** | | | | | |
| **Osat_14** | | **Bdist_30** | | **Zmays_53** | |
| **Osat_15** | **Ataus_15** | **Bdist_10** | | **Zmays_57** | **Macum_44** |
| | | | | Zmays_55 | Macum_37 |
| | | | | Zmays_56 | Macum_65 |
| | | | | Zmays_85 | |
| | | | | Zmays_86 | |
| **Osat_16** | | **Bdist_25** | **Hvulg_25** | **Zmays_31** | |
| | | | Hvulg_56 | Zmays_69 | |
| | | | Hvulg_57 | Zmays_161 | |
| | | | Hvulg_61 | | |
| | | | Hvulg_62 | | |
| **Osat_17** | **Ataus_16** | **Bdist_12** | **Hvulg_26** | **Zmays_25** | **Macum_38** |
| | Ataus_9 | | Hvulg_14 | Zmays_127 | Macum_58 |
| | Ataus_65 | | Hvulg_110 | | |
| | Ataus_66 | | Hvulg_113 | | |

**Table S4** Continued

| | | | | | |
|---|---|---|---|---|---|
| **Osat_18** | | | **Hvulg_44** | | |
| | | | Hvulg_19 | | |
| | | | Hvulg_78 | | |
| | | | Hvulg_79 | | |
| **Osat_19** | | **Bdist_8** | **Hvulg_13** | **Zmays_14** | **Macum_18** |
| | | Bdist_60 | Hvulg_11 | Zmays_13 | Macum_63 |
| | | | Hvulg_12 | | |
| **Osat_20** | **Ataus_19** | **Bdist_22** | **Hvulg_37** | **Zmays_30** | **Macum_40** |
| | Ataus_86 | | Hvulg_30 | Zmays_44 | Macum_16 |
| | Ataus_88 | | Hvulg_38 | Zmays_65 | Macum_20 |
| | | | Hvulg_39 | | Macum_23 |
| | | | Hvulg_63 | | Macum_25 |
| | | | Hvulg_100 | | Macum_29 |
| | | | | | Macum_39 |
| | | | | | Macum_49 |
| | | | | | Macum_55 |
| **Osat_21** | **Ataus_24** | **Bdist_20** | **Hvulg_24** | **Zmays_41** | **Macum_52** |
| | | | Hvulg_41 | Zmays_42 | Macum_30 |
| | | | Hvulg_133 | Zmays_63 | Macum_48 |
| | | | | Zmays_133 | Macum_51 |
| | | | | | Macum_54 |
| **Osat_22** | **Ataus_29** | **Bdist_24** | **Hvulg_49** | **Zmays_59** | |
| | Ataus_26 | | Hvulg_121 | Zmays_52 | |
| | Ataus_31 | | | | |
| | Ataus_37 | | | | |
| **Osat_23** | **Ataus_22** | | **Hvulg_18** | **Zmays_35** | **Macum_59** |
| | | | Hvulg_20 | Zmays_34 | Macum_66 |
| | | | Hvulg_23 | | |
| **Osat_24** | | **Bdist_19** | | **Zmays_21** | |
| | | | | Zmays_20 | |
| | | | | Zmays_163 | |
| **Osat_25** | **Ataus_6** | | **Hvulg_9** | **Zmays_54** | |
| **Osat_26** | | | | | |
| **Osat_27** | | | | **Zmays_49** | |
| **Osat_28** | | | | | |
| **Osat_29** | **Ataus_36** | **Bdist_43** | **Hvulg_66** | **Zmays_81** | **Macum_47** |
| | Ataus_28 | Bdist_53 | Hvulg_53 | Zmays_61 | Macum_91 |
| | Ataus_39 | | Hvulg_54 | Zmays_104 | Macum_138 |
| | Ataus_54 | | Hvulg_55 | | |
| | | | Hvulg_80 | | |
| | | | Hvulg_81 | | |
| | | | Hvulg_84 | | |
| | | | Hvulg_89 | | |
| | | | Hvulg_139 | | |

**Table S4** Continued

| | | | | | |
|---|---|---|---|---|---|
| **Osat_30** | **Ataus_35** | **Bdist_28** | **Hvulg_35** | **Zmays_50** | **Macum_61** |
| | Ataus_38 | Bdist_26 | | Zmays_47 | Macum_64 |
| | Ataus_48 | Bdist_27 | | Zmays_48 | |
| | Ataus_70 | | | | |
| **Osat_31** | | | | | |
| **Osat_32** | **Ataus_18** | **Bdist_23** | **Hvulg_28** | **Zmays_45** | **Macum_35** |
| | | Bdist_21 | | Zmays_39 | Macum_36 |
| | | | | Zmays_40 | Macum_60 |
| | | | | Zmays_78 | Macum_148 |
| **Osat_33** | | | **Hvulg_42** | **Zmays_66** | **Macum_69** |
| | | | | Zmays_67 | |
| | | | | Zmays_84 | |
| **Osat_34** | | **Bdist_18** | | **Zmays_46** | |
| | | | | Zmays_74 | |
| | | | | Zmays_89 | |
| **Osat_35** | | | | | |
| **Osat_36** | | | | | |
| **Osat_37** | **Ataus_42** | **Bdist_42** | | **Zmays_72** | |
| | | Bdist_69 | | Zmays_73 | |
| **Osat_38** | **Ataus_30** | | **Hvulg_43** | **Zmays_64** | |
| **Osat_39** | | | | | |
| **Osat_40** | | | | | |
| **Osat_41** | **Ataus_53** | **Bdist_36** | **Hvulg_77** | **Zmays_109** | |
| | | | Hvulg_92 | | |
| **Osat_42** | | **Bdist_37** | **Hvulg_59** | **Zmays_95** | **Macum_84** |
| | | | Hvulg_58 | Zmays_82 | Macum_70 |
| | | | Hvulg_65 | Zmays_91 | Macum_71 |
| | | | Hvulg_141 | Zmays_173 | Macum_78 |
| | | | | | Macum_82 |
| | | | | | Macum_101 |
| | | | | | Macum_102 |
| | | | | | Macum_114 |
| | | | | | Macum_134 |
| **Osat_43** | | | | **Zmays_58** | |
| **Osat_44** | **Ataus_40** | **Bdist_33** | **Hvulg_60** | **Zmays_71** | **Macum_72** |
| | Ataus_47 | Bdist_48 | Hvulg_69 | Zmays_92 | Macum_67 |
| | | | Hvulg_114 | Zmays_94 | Macum_68 |
| | | | Hvulg_123 | Zmays_96 | Macum_73 |
| | | | | Zmays_97 | Macum_85 |
| | | | | Zmays_129 | Macum_87 |
| | | | | | Macum_106 |

**Table S4** Continued

| | | | | | |
|---|---|---|---|---|---|
| **Osat_45** | | | | | |
| **Osat_46** | **Ataus_62** | **Bdist_41** | **Hvulg_68** | **Zmays_108** | **Macum_117** |
| | | | Hvulg_83 | Zmays_105 | Macum_119 |
| **Osat_47** | **Ataus_44** | **Bdist_34** | **Hvulg_87** | **Zmays_80** | **Macum_77** |
| | Ataus_59 | Bdist_32 | Hvulg_94 | Zmays_75 | Macum_62 |
| | Ataus_63 | Bdist_89 | | Zmays_76 | Macum_76 |
| | | | | Zmays_79 | |
| | | | | Zmays_102 | |
| | | | | Zmays_103 | |
| | | | | Zmays_111 | |
| **Osat_48** | **Ataus_58** | **Bdist_46** | **Hvulg_91** | **Zmays_98** | **Macum_97** |
| | Ataus_67 | | | Zmays_99 | Macum_86 |
| | | | | Zmays_112 | Macum_103 |
| | | | | Zmays_135 | Macum_123 |
| | | | | | Macum_151 |
| **Osat_49** | **Ataus_49** | **Bdist_40** | **Hvulg_67** | | |
| | | | Hvulg_82 | | |
| | | | Hvulg_118 | | |
| **Osat_50** | **Ataus_51** | **Bdist_38** | **Hvulg_73** | **Zmays_90** | **Macum_105** |
| | Ataus_52 | Bdist_47 | Hvulg_72 | Zmays_100 | Macum_75 |
| | Ataus_75 | | Hvulg_86 | Zmays_106 | Macum_80 |
| | Ataus_87 | | Hvulg_93 | Zmays_114 | Macum_94 |
| | Ataus_111 | | | Zmays_140 | Macum_112 |
| | | | | | Macum_115 |
| | | | | | Macum_131 |
| **Osat_51** | | | | | |
| **Osat_52** | **Ataus_50** | **Bdist_45** | **Hvulg_76** | **Zmays_115** | **Macum_111** |
| | Ataus_81 | Bdist_44 | Hvulg_70 | Zmays_138 | Macum_96 |
| | Ataus_85 | | Hvulg_71 | | Macum_104 |
| | | | Hvulg_74 | | Macum_107 |
| | | | Hvulg_75 | | Macum_120 |
| | | | | | Macum_122 |
| | | | | | Macum_162 |
| **Osat_53** | **Ataus_60** | **Bdist_49** | **Hvulg_88** | **Zmays_107** | **Macum_99** |
| | Ataus_61 | | Hvulg_85 | Zmays_113 | Macum_81 |
| | | | Hvulg_95 | | Macum_88 |
| | | | | | Macum_89 |
| | | | | | Macum_92 |
| | | | | | Macum_95 |
| | | | | | Macum_98 |
| | | | | | Macum_100 |
| | | | | | Macum_108 |
| | | | | | Macum_121 |

**Table S4** Continued

| | | | | | |
|---|---|---|---|---|---|
| **Osat_54** | | | | | |
| **Osat_55** | **Ataus_56** | **Bdist_50** | **Hvulg_90** | **Zmays_110** | |
| | Ataus_1 | | | Zmays_101 | |
| | Ataus_57 | | | Zmays_132 | |
| | Ataus_107 | | | Zmays_172 | |
| **Osat_56** | | | | | |
| **Osat_57** | | **Bdist_55** | **Hvulg_96** | **Zmays_120** | **Macum_116** |
| | | | | Zmays_117 | Macum_124 |
| | | | | | Macum_129 |
| | | | | | Macum_152 |
| **Osat_58** | **Ataus_41** | **Bdist_35** | | **Zmays_70** | |
| **Osat_59** | | **Bdist_52** | **Hvulg_138** | **Zmays_116** | **Macum_143** |
| | | Bdist_51 | Hvulg_137 | | Macum_133 |
| | | | | | Macum_141 |
| **Osat_60** | | **Bdist_57** | | **Zmays_118** | |
| | | | | Zmays_119 | |
| | | | | Zmays_137 | |
| **Osat_61** | | | | | |
| **Osat_62** | | **Bdist_56** | | **Zmays_121** | |
| | | | | Zmays_146 | |
| **Osat_63** | | | | | |
| **Osat_64** | | **Bdist_54** | | **Zmays_122** | |
| | | | | Zmays_180 | |
| **Osat_65** | | **Bdist_59** | **Hvulg_98** | **Zmays_126** | **Macum_136** |
| | | | | Zmays_130 | Macum_137 |
| | | | | | Macum_145 |
| **Osat_66** | **Ataus_68** | **Bdist_58** | | **Zmays_125** | |
| **Osat_67** | | | | | |
| **Osat_68** | **Ataus_69** | **Bdist_61** | **Hvulg_99** | **Zmays_123** | **Macum_140** |
| **Osat_69** | | | | | |
| **Osat_70** | | **Bdist_64** | | | |
| **Osat_71** | | | | | |
| **Osat_72** | | | | | |
| **Osat_73** | | **Bdist_66** | | **Zmays_128** | **Macum_146** |
| | | | | | Macum_147 |
| | | | | | Macum_149 |
| | | | | | Macum_168 |

**Table S4** Continued

| | | | | | |
|---|---|---|---|---|---|
| **Osat_74** | | | | | |
| **Osat_75** | | | | | |
| **Osat_76** | | | | | |
| **Osat_77** | **Ataus_74** | | **Hvulg_103** | | |
| **Osat_78** | **Ataus_34** | | **Hvulg_102** | | |
| | | | Hvulg_51 | | |
| **Osat_79** | | | | | |
| **Osat_80** | **Ataus_77** | | **Hvulg_104** | **Zmays_141** | |
| | | | | Zmays_142 | |
| | | | | Zmays_144 | |
| | | | | Zmays_175 | |
| **Osat_81** | | | | | |
| **Osat_82** | | | | | |
| **Osat_83** | | | | | |
| **Osat_84** | | | | | |
| **Osat_85** | | | | | |
| **Osat_86** | | | | | |
| **Osat_87** | **Ataus_92** | **Bdist_71** | **Hvulg_111** | **Zmays_157** | **Macum_157** |
| | | Bdist_72 | Hvulg_106 | Zmays_155 | Macum_154 |
| | | Bdist_77 | Hvulg_107 | Zmays_156 | Macum_155 |
| | | | Hvulg_108 | Zmays_158 | Macum_156 |
| | | | Hvulg_109 | Zmays_186 | Macum_158 |
| | | | Hvulg_112 | | |
| | | | Hvulg_115 | | |
| **Osat_88** | | | | | |
| **Osat_89** | | | | | |
| **Osat_90** | | | | | |
| **Osat_91** | | | | | |

**Table S4** Continued

| | | | | | |
|---|---|---|---|---|---|
| **Osat_92** | | | | | |
| **Osat_93** | | | | | |
| 5 **Osat_94** | | **Bdist_76** | | **Zmays_160** | **Macum_161** |
| | | | | Zmays_159 | Macum_159 |
| | | | | Zmays_162 | Macum_163 |
| | | | | Zmays_166 | |
| | | | | Zmays_169 | |
| | | | | Zmays_183 | |
| **Osat_95** | | | **Hvulg_122** | **Zmays_164** | |
| | | | Hvulg_120 | | |
| **Osat_96** | | | | | |
| **Osat_97** | **Ataus_83** | | | | |
| **Osat_98** | | | | | |
| **Osat_99** | | | | | |
| **Osat_100** | | | | | |
| **Osat_101** | | | | | |
| **Osat_102** | **Ataus_90** | **Bdist_80** | **Hvulg_131** | **Zmays_182** | **Macum_165** |
| | Ataus_89 | Bdist_73 | Hvulg_128 | Zmays_165 | Macum_160 |
| | Ataus_94 | Bdist_78 | Hvulg_129 | Zmays_168 | Macum_164 |
| | Ataus_98 | | Hvulg_130 | Zmays_174 | |
| | Ataus_100 | | Hvulg_134 | Zmays_176 | |
| | Ataus_101 | | | Zmays_177 | |
| | | | | Zmays_181 | |
| | | | | Zmays_184 | |
| **Osat_103** | | | | | |
| **Osat_104** | | | | | |
| **Osat_105** | | | | | |
| **Osat_106** | | | | | |
