# Supplementary material for: Comparative Genomics of NAC Transcriptional Factors in Angiosperms: Implications for the Adaptation and Diversification of Flowering Plants
Source: PLoS One. 2015 Nov 16;10(11):e0141866. doi: 10.1371/journal.pone.0141866 (PMC4646352; doi:10.1371/journal.pone.0141866)
Supplement: S5 Table — List of OG proteins from the Class Magnoliopsida using the Vitis vinifera sequences as references. Purple colored blocks represent orthologous sequences. Species-specific duplications of each gene are shown below the colored blocks. Sequences belonging to the basal orthologous groups are numbered and marked with yellow stars. (PDF) [file pone.0141866.s010.pdf]

Table S5

OGs of NAC proteins in Eudicots. Purple colored blocks represents orthologous sequences.Paralogous genes are shown below the colored blocks. Sequences belonging to the BOG are numbered and marked with yellow stars.

**Vvin_1**															
**Vvin_2**	**Tcac_1**	**Cpap_1**	**Athal_1**	**Brapa_1**	**Thalo_1**	**Pvul_2**	**Gmax_4**	**Mtrunc_6**	**Rcomm_1**	**Mesc_1**	**Ptric_1**	**Mdom_2**	**Csat_1**	**Slyc_2**	**Nbent_5**
	Tcac_94	Cpap_29	Athal_105	Brapa_123	Thalo_85	Pvul_1	Gmax_1	Mtrunc_4		Mesc_102	Ptric_57	Mdom_6		Slyc_4	Nbent_3
	Tcac_95	Cpap_35	Athal_126	Brapa_195			Gmax_3	Mtrunc_29		Mesc_122	Ptric_141	Mdom_110		Slyc_70	Nbent_101
	Tcac_104						Gmax_5					Mdom_140		Slyc_96	Nbent_138
												Mdom_180			Nbent_141
												Mdom_230			Nbent_144
															Nbent_145
															Nbent_146
															Nbent_186
															Nbent_187
															Nbent_194
															Nbent_201
															Nbent_204
**Vvin_3**	**Tcac_2**	**Cpap_2**	**Athal_4**	**Brapa_7**	**Thalo_3**	**Pvul_4**	**Gmax_12**		**Rcomm_3**	**Mesc_5**	**Ptric_4**	**Mdom_1**	**Csat_3**	**Slyc_1**	**Nbent_8**
		Cpap_7	Athal_9	Brapa_5	Thalo_8	Pvul_7	Gmax_6		Rcomm_63	Mesc_10	Ptric_3	Mdom_3	Csat_8	Slyc_8	Nbent_1
			Athal_17	Brapa_10			Gmax_10		Rcomm_89		Ptric_146	Mdom_16			Nbent_6
			Athal_96	Brapa_21			Gmax_11		Rcomm_91		Ptric_233	Mdom_162			Nbent_104
			Athal_123				Gmax_15					Mdom_170			Nbent_154
							Gmax_19								
★4 **Vvin_4**	**Tcac_4**		**Athal_5**	**Brapa_6**	**Thalo_6**	**Pvul_3**	**Gmax_9**		**Rcomm_4**		**Ptric_6**				
	Tcac_57		Athal_12			Pvul_6			Rcomm_53		Ptric_8				
			Athal_129						Rcomm_54		Ptric_12				
									Rcomm_85		Ptric_139				
											Ptric_223				
											Ptric_224				
											Ptric_225				
**Vvin_5**	**Tcac_3**	**Cpap_9**	**Athal_26**	**Brapa_24**	**Thalo_21**	**Pvul_22**	**Gmax_59**	**Mtrunc_17**	**Rcomm_2**	**Mesc_4**	**Ptric_2**				
			Athal_23	Brapa_34	Thalo_29	Pvul_31	Gmax_66			Mesc_6	Ptric_13				
			Athal_25	Brapa_38			Gmax_71				Ptric_148				
				Brapa_46			Gmax_74								
				Brapa_72											
**Vvin_6**	**Tcac_11**	**Cpap_12**					**Gmax_25**	**Mtrunc_21**	**Rcomm_6**	**Mesc_9**	**Ptric_15**	**Mdom_10**		**Slyc_13**	**Nbent_24**
	Tcac_6						Gmax_16	Mtrunc_25	Rcomm_27	Mesc_12	Ptric_9	Mdom_4		Slyc_22	Nbent_34
							Gmax_32	Mtrunc_33		Mesc_21	Ptric_17	Mdom_5			Nbent_65
							Gmax_42	Mtrunc_36		Mesc_104	Ptric_171				Nbent_66
							Gmax_78	Mtrunc_53							Nbent_102
							Gmax_92								
							Gmax_93								
							Gmax_94								
							Gmax_95								
							Gmax_96								

Table S5 Continued

**Vvin_7**	**Tcac_8**	**Cpap_5**	**Athal_10**	**Brapa_14**	**Thalo_11**	**Pvul_9**	**Gmax_48**	**Mtrunc_5**	**Rcomm_5**	**Mesc_7**	**Ptric_10**		**Csat_6**	**Slyc_3**	**Nbent_11**
			Athal_21	Brapa_51	Thalo_22	Pvul_13	Gmax_24				Ptric_20		Csat_2	Slyc_12	Nbent_12
							Gmax_26				Ptric_71			Slyc_90	Nbent_29
							Gmax_27				Ptric_72				Nbent_48
							Gmax_31				Ptric_175				Nbent_79
															Nbent_99
															Nbent_100
															Nbent_122
															Nbent_188
															Nbent_203
**Vvin_8**		**Cpap_3**	**Athal_2**	**Brapa_3**	**Thalo_5**	**Pvul_16**					**Ptric_5**	**Mdom_11**	**Csat_11**	**Slyc_15**	**Nbent_23**
			Athal_6	Brapa_2	Thalo_2	Pvul_11					Ptric_18	Mdom_20	Csat_22		Nbent_119
				Brapa_4	Thalo_4								Csat_28		Nbent_121
				Brapa_8									Csat_66		
				Brapa_19									Csat_67		
													Csat_78		
**Vvin_9**	**Tcac_18**	**Cpap_14**					**Gmax_43**		**Rcomm_14**	**Mesc_14**	**Ptric_40**	**Mdom_18**	**Csat_7**	**Slyc_6**	**Nbent_9**
	Tcac_117						Gmax_33		Rcomm_76	Mesc_126	Ptric_42	Mdom_224		Slyc_45	Nbent_10
	Tcac_118						Gmax_177		Rcomm_78		Ptric_43	Mdom_239			Nbent_136
	Tcac_120										Ptric_44	Mdom_243			Nbent_155
	Tcac_121														Nbent_205
	Tcac_123														
**Vvin_10**	**Tcac_14**	**Cpap_10**	**Athal_16**	**Brapa_11**	**Thalo_10**	**Pvul_17**	**Gmax_45**		**Rcomm_9**	**Mesc_20**	**Ptric_25**	**Mdom_40**	**Csat_12**	**Slyc_9**	**Nbent_13**
			Athal_27	Brapa_27	Thalo_37	Pvul_23	Gmax_49			Mesc_15	Ptric_36	Mdom_51	Csat_14		Nbent_18
				Brapa_58			Gmax_50				Ptric_37				
				Brapa_65			Gmax_51								
**Vvin_11**	**Tcac_13**	**Cpap_6**	**Athal_14**	**Brapa_15**	**Thalo_13**	**Pvul_19**	**Gmax_21**	**Mtrunc_11**	**Rcomm_13**	**Mesc_25**	**Ptric_28**	**Mdom_7**	**Csat_4**	**Slyc_11**	**Nbent_16**
	Tcac_115		Athal_29	Brapa_35	Thalo_28	Pvul_15	Gmax_28	Mtrunc_7		Mesc_13	Ptric_48	Mdom_44		Slyc_10	Nbent_14
			Athal_32	Brapa_40	Thalo_30		Gmax_30							Slyc_65	Nbent_15
				Brapa_41	Thalo_35		Gmax_52							Slyc_74	Nbent_39
				Brapa_144											Nbent_120
				Brapa_147											Nbent_132
**Vvin_12**	**Tcac_12**	**Cpap_4**	**Athal_7**	**Brapa_9**	**Thalo_9**	**Pvul_18**	**Gmax_34**		**Rcomm_8**	**Mesc_18**	**Ptric_16**	**Mdom_14**	**Csat_5**	**Slyc_7**	**Nbent_7**
	Tcac_75	Cpap_52	Athal_18	Brapa_23	Thalo_15	Pvul_5	Gmax_22		Rcomm_70	Mesc_8	Ptric_14	Mdom_15	Csat_9	Slyc_5	Nbent_17
	Tcac_76	Cpap_58	Athal_19	Brapa_31	Thalo_24		Gmax_23		Rcomm_72			Mdom_219	Csat_32	Slyc_67	
	Tcac_96	Cpap_70		Brapa_45	Thalo_94		Gmax_29					Mdom_227			
	Tcac_97	Cpap_71		Brapa_53	Thalo_95		Gmax_35								
	Tcac_98	Cpap_72		Brapa_54	Thalo_114										
	Tcac_100	Cpap_73			Thalo_120										
	Tcac_127														
**Vvin_13**															
**Vvin_14**	**Tcac_5**	**Cpap_21**	**Athal_31**	**Brapa_57**	**Thalo_17**	**Pvul_27**	**Gmax_44**	**Mtrunc_13**	**Rcomm_17**	**Mesc_17**	**Ptric_45**	**Mdom_12**	**Csat_13**		
				Brapa_77		Pvul_91	Gmax_38		Rcomm_51		Ptric_41	Mdom_29	Csat_81		
									Rcomm_81		Ptric_180	Mdom_175			
											Ptric_259	Mdom_176			
											Ptric_260	Mdom_207			
												Mdom_210			
												Mdom_238			
												Mdom_244			

Table S5 Continued

**Vvin_15**	**Tcac_21**	**Cpap_20**	**Athal_15**	**Brapa_36**	**Thalo_23**	**Pvul_41**	**Gmax_79**	**Mtrunc_19**	**Rcomm_22**	**Mesc_35**	**Ptric_55**	**Mdom_21**	**Csat_27**	**Slyc_23**	**Nbent_27**
		Cpap_54		Brapa_18	Thalo_39	Pvul_46	Gmax_84			Mesc_27	Ptric_50	Mdom_28	Csat_79	Slyc_26	Nbent_26
				Brapa_42			Gmax_104				Ptric_51	Mdom_36			Nbent_32
							Gmax_118					Mdom_171			Nbent_110
															Nbent_162
**Vvin_16**	**Tcac_15**	**Cpap_13**	**Athal_28**	**Brapa_43**	**Thalo_27**	**Pvul_34**	**Gmax_63**	**Mtrunc_14**	**Rcomm_12**	**Mesc_22**	**Ptric_53**	**Mdom_41**	**Csat_15**		
	Tcac_16		Athal_33	Brapa_47	Thalo_19	Pvul_33	Gmax_47	Mtrunc_18		Mesc_93	Ptric_19	Mdom_42	Csat_30		
	Tcac_101			Brapa_50		Pvul_37	Gmax_53				Ptric_26	Mdom_60			
	Tcac_102			Brapa_97		Pvul_38	Gmax_62				Ptric_279	Mdom_62			
**Vvin_17**	**Tcac_10**	**Cpap_11**	**Athal_11**	**Brapa_12**	**Thalo_12**	**Pvul_40**	**Gmax_57**	**Mtrunc_26**	**Rcomm_10**	**Mesc_23**	**Ptric_23**	**Mdom_30**	**Csat_16**	**Slyc_19**	**Nbent_22**
				Brapa_13		Pvul_28	Gmax_13	Mtrunc_20			Ptric_22	Mdom_27		Slyc_16	Nbent_52
						Pvul_29	Gmax_14	Mtrunc_67			Ptric_24	Mdom_155		Slyc_78	Nbent_137
						Pvul_32	Gmax_17					Mdom_181			
							Gmax_20					Mdom_187			
							Gmax_36					Mdom_188			
							Gmax_37					Mdom_202			
							Gmax_40					Mdom_212			
							Gmax_41								
							Gmax_54								
							Gmax_55								
							Gmax_58								
							Gmax_181								
1 **Vvin_18**	**Tcac_19**	**Cpap_8**				**Pvul_26**	**Gmax_56**		**Rcomm_11**	**Mesc_19**	**Ptric_21**	**Mdom_83**		**Slyc_24**	**Nbent_31**
	Tcac_106					Pvul_21	Gmax_46				Ptric_27	Mdom_64		Slyc_18	Nbent_37
	Tcac_122						Gmax_60				Ptric_32	Mdom_211			Nbent_38
	Tcac_132						Gmax_64				Ptric_246				Nbent_50
							Gmax_65								Nbent_191
							Gmax_68								Nbent_193
							Gmax_72								Nbent_200
**Vvin_19**	**Tcac_17**	**Cpap_18**				**Pvul_36**	**Gmax_61**	**Mtrunc_16**	**Rcomm_15**	**Mesc_24**	**Ptric_62**	**Mdom_43**			
	Tcac_40						Gmax_102	Mtrunc_65	Rcomm_71	Mesc_95	Ptric_68	Mdom_163			
							Gmax_125			Mesc_101	Ptric_70	Mdom_164			
							Gmax_150				Ptric_149	Mdom_166			
							Gmax_162					Mdom_167			
							Gmax_168					Mdom_168			
												Mdom_169			
												Mdom_189			
												Mdom_218			
**Vvin_20**	**Tcac_7**			**Brapa_49**	**Thalo_14**	**Pvul_8**	**Gmax_2**	**Mtrunc_8**	**Rcomm_7**	**Mesc_16**	**Ptric_7**	**Mdom_9**			
	Tcac_9			Brapa_25		Pvul_12	Gmax_18			Mesc_3	Ptric_11	Mdom_8			
	Tcac_128						Gmax_39								
							Gmax_109								
**Vvin_21**	**Tcac_28**		**Athal_22**	**Brapa_30**	**Thalo_18**	**Pvul_30**	**Gmax_76**	**Mtrunc_22**	**Rcomm_18**	**Mesc_30**	**Ptric_65**	**Mdom_22**	**Csat_23**	**Slyc_27**	**Nbent_19**
			Athal_35	Brapa_29	Thalo_34		Gmax_70			Mesc_39	Ptric_33	Mdom_23			Nbent_72
															Nbent_95
															Nbent_117

Table S5 Continued

**Vvin_22**	**Tcac_23**	**Cpap_17**	**Athal_24**	**Brapa_32**	**Thalo_25**	**Pvul_39**	**Gmax_83**		**Rcomm_19**	**Mesc_26**	**Ptric_39**	**Mdom_31**	**Csat_18**	**Slyc_14**	**Nbent_21**
		Cpap_48			Thalo_92		Gmax_82				Ptric_29	Mdom_26	Csat_82		Nbent_179
											Ptric_30	Mdom_233			
											Ptric_38				
											Ptric_234				
											Ptric_271				
**Vvin_23**	**Tcac_26**	**Cpap_16**	**Athal_36**	**Brapa_71**	**Thalo_33**	**Pvul_35**	**Gmax_73**	**Mtrunc_15**	**Rcomm_16**	**Mesc_29**	**Ptric_34**	**Mdom_19**	**Csat_24**	**Slyc_20**	**Nbent_28**
	Tcac_24			Brapa_39			Gmax_77			Mesc_28	Ptric_31	Mdom_17			Nbent_40
	Tcac_25			Brapa_63								Mdom_55			Nbent_199
**Vvin_24**	**Tcac_22**					**Pvul_20**	**Gmax_67**	**Mtrunc_10**	**Rcomm_21**	**Mesc_32**		**Mdom_47**			
	Tcac_74						Gmax_75		Rcomm_67			Mdom_45			
									Rcomm_88			Mdom_46			
												Mdom_231			
**Vvin_25**	**Tcac_27**	**Cpap_24**	**Athal_34**	**Brapa_44**	**Thalo_26**	**Pvul_43**	**Gmax_90**	**Mtrunc_24**	**Rcomm_20**	**Mesc_37**	**Ptric_47**	**Mdom_38**	**Csat_21**	**Slyc_21**	**Nbent_25**
	Tcac_20		Athal_30	Brapa_37	Thalo_32	Pvul_48	Gmax_85	Mtrunc_23		Mesc_31	Ptric_35	Mdom_37			Nbent_53
	Tcac_107		Athal_45	Brapa_48	Thalo_40		Gmax_86			Mesc_33	Ptric_46	Mdom_39			Nbent_114
	Tcac_111			Brapa_66			Gmax_87				Ptric_49	Mdom_50			Nbent_140
							Gmax_88				Ptric_56				Nbent_142
							Gmax_89				Ptric_59				Nbent_143
							Gmax_91				Ptric_60				
											Ptric_64				
**Vvin_26**	**Tcac_29**								**Rcomm_23**	**Mesc_36**	**Ptric_52**	**Mdom_35**	**Csat_26**		
											Ptric_63	Mdom_24			
											Ptric_285	Mdom_25			
												Mdom_95			
**Vvin_27**	**Tcac_31**	**Cpap_22**	**Athal_40**	**Brapa_56**	**Thalo_36**	**Pvul_42**	**Gmax_81**		**Rcomm_25**	**Mesc_43**	**Ptric_66**	**Mdom_33**	**Csat_25**	**Slyc_32**	**Nbent_30**
			Athal_44	Brapa_61	Thalo_44		Gmax_80			Mesc_40	Ptric_61	Mdom_48		Slyc_29	Nbent_20
			Athal_46	Brapa_62						Mesc_96	Ptric_67	Mdom_143			Nbent_41
				Brapa_68											Nbent_42
				Brapa_74											Nbent_63
				Brapa_76											
**Vvin_28**	**Tcac_30**	**Cpap_19**	**Athal_42**	**Brapa_64**	**Thalo_42**	**Pvul_47**	**Gmax_98**		**Rcomm_24**	**Mesc_34**	**Ptric_54**	**Mdom_34**		**Slyc_25**	**Nbent_36**
				Brapa_70			Gmax_97			Mesc_42	Ptric_58	Mdom_32		Slyc_28	Nbent_33
				Brapa_174			Gmax_99				Ptric_80				Nbent_35
							Gmax_100								Nbent_106
							Gmax_101								
**Vvin_29**	**Tcac_38**	**Cpap_25**	**Athal_57**	**Brapa_78**	**Thalo_43**	**Pvul_56**	**Gmax_122**	**Mtrunc_44**	**Rcomm_28**	**Mesc_45**	**Ptric_87**	**Mdom_53**	**Csat_34**	**Slyc_37**	**Nbent_51**
			Athal_37	Brapa_55	Thalo_41		Gmax_120	Mtrunc_43	Rcomm_64		Ptric_78	Mdom_54		Slyc_34	Nbent_54
			Athal_72	Brapa_59	Thalo_63		Gmax_197				Ptric_81	Mdom_160		Slyc_66	Nbent_60
				Brapa_94			Gmax_198				Ptric_84	Mdom_226		Slyc_93	Nbent_71
				Brapa_113			Gmax_199				Ptric_85				
				Brapa_161							Ptric_86				
											Ptric_215				
											Ptric_219				
											Ptric_226				
											Ptric_227				
											Ptric_230				
											Ptric_231				
											Ptric_232				
											Ptric_258				

Table S5 Continued

**Vvin_30**	**Tcac_32**	**Cpap_23**	**Athal_39**	**Brapa_79**	**Thalo_49**	**Pvul_52**	**Gmax_106**	**Mtrunc_30**	**Rcomm_26**	**Mesc_44**	**Ptric_73**	**Mdom_52**	**Csat_29**		**Nbent_46**
		Cpap_51	Athal_111	Brapa_73	Thalo_93		Gmax_108			Mesc_41	Ptric_69	Mdom_71	Csat_45		Nbent_43
			Athal_116		Thalo_112							Mdom_117	Csat_47		Nbent_149
					Thalo_116										
					Thalo_117										
					Thalo_118										
**Vvin_31**	**Tcac_39**	**Cpap_32**	**Athal_48**	**Brapa_80**	**Thalo_48**	**Pvul_53**	**Gmax_119**	**Mtrunc_32**	**Rcomm_31**	**Mesc_55**		**Mdom_59**	**Csat_41**	**Slyc_31**	
	Tcac_72			Brapa_86	Thalo_45	Pvul_67	Gmax_111			Mesc_47		Mdom_58	Csat_44	Slyc_35	
				Brapa_104			Gmax_112					Mdom_191			
							Gmax_113								
							Gmax_188								
**Vvin_32**	**Tcac_37**	**Cpap_26**	**Athal_66**	**Brapa_91**	**Thalo_58**	**Pvul_51**	**Gmax_107**	**Mtrunc_31**	**Rcomm_32**	**Mesc_48**	**Ptric_82**	**Mdom_67**	**Csat_37**	**Slyc_41**	**Nbent_68**
			Athal_68	Brapa_100	Thalo_64	Pvul_54	Gmax_121	Mtrunc_49	Rcomm_36	Mesc_56	Ptric_79	Mdom_66	Csat_51	Slyc_61	Nbent_70
			Athal_69	Brapa_101	Thalo_90	Pvul_66	Gmax_154	Mtrunc_51	Rcomm_69	Mesc_67	Ptric_83	Mdom_79	Csat_71		Nbent_96
			Athal_107	Brapa_102		Pvul_71	Gmax_155	Mtrunc_61		Mesc_118	Ptric_102	Mdom_82			Nbent_107
				Brapa_112		Pvul_90	Gmax_158	Mtrunc_64		Mesc_125	Ptric_103				Nbent_109
				Brapa_167			Gmax_178				Ptric_115				Nbent_115
							Gmax_179				Ptric_122				Nbent_151
							Gmax_186				Ptric_124				Nbent_152
							Gmax_196				Ptric_178				Nbent_153
							Gmax_203				Ptric_179				
											Ptric_210				
											Ptric_211				
											Ptric_217				
											Ptric_220				
											Ptric_221				
											Ptric_261				
**Vvin_33**			**Athal_56**		**Thalo_46**	**Pvul_49**	**Gmax_103**		**Rcomm_30**	**Mesc_52**	**Ptric_91**				**Nbent_49**
							Gmax_105			Mesc_50	Ptric_188				Nbent_118
										Mesc_109	Ptric_214				
**Vvin_34**	**Tcac_42**		**Athal_59**	**Brapa_84**	**Thalo_54**	**Pvul_55**	**Gmax_124**		**Rcomm_33**	**Mesc_54**	**Ptric_95**	**Mdom_76**	**Csat_42**	**Slyc_39**	**Nbent_69**
			Athal_58	Brapa_82			Gmax_123			Mesc_53	Ptric_92	Mdom_73			Nbent_67
			Athal_127	Brapa_98						Mesc_108	Ptric_112				Nbent_73
				Brapa_177							Ptric_113				Nbent_124
				Brapa_192											Nbent_135
**Vvin_35**	**Tcac_44**		**Athal_47**	**Brapa_81**	**Thalo_47**	**Pvul_64**	**Gmax_149**		**Rcomm_29**	**Mesc_51**	**Ptric_75**	**Mdom_57**	**Csat_36**	**Slyc_36**	**Nbent_64**
	Tcac_43		Athal_49	Brapa_75	Thalo_55		Gmax_192		Rcomm_86	Mesc_114	Ptric_74	Mdom_56	Csat_43	Slyc_30	Nbent_45
				Brapa_89					Rcomm_94		Ptric_76	Mdom_68	Csat_88		Nbent_55
				Brapa_105					Rcomm_95		Ptric_88	Mdom_149	Csat_95		Nbent_58
											Ptric_89	Mdom_203			Nbent_148
											Ptric_90	Mdom_234			Nbent_157
											Ptric_273	Mdom_235			Nbent_158
															Nbent_159
															Nbent_160
															Nbent_161
															Nbent_175
															Nbent_192
															Nbent_197
															Nbent_206

Table S5 Continued

**Vvin_36**	**Tcac_46**	**Cpap_34**	**Athal_67**	**Brapa_106**	**Thalo_61**	**Pvul_59**	**Gmax_132**	**Mtrunc_38**	**Rcomm_34**	**Mesc_63**		**Mdom_69**	**Csat_40**	**Slyc_48**	**Nbent_76**
	Tcac_48	Cpap_41	Athal_71	Brapa_109	Thalo_59	Pvul_57	Gmax_126	Mtrunc_35	Rcomm_61	Mesc_106				Slyc_43	Nbent_75
	Tcac_83	Cpap_53		Brapa_110	Thalo_60		Gmax_130	Mtrunc_37							Nbent_84
	Tcac_84			Brapa_111			Gmax_135	Mtrunc_39							Nbent_86
	Tcac_85			Brapa_143			Gmax_237	Mtrunc_45							Nbent_182
**Vvin_37**		**Cpap_44**				**Pvul_72**		**Mtrunc_47**		**Mesc_71**	**Ptric_140**	**Mdom_85**		**Slyc_51**	**Nbent_103**
											Ptric_197	Mdom_232			Nbent_85
**Vvin_38**	**Tcac_33**	**Cpap_28**	**Athal_62**	**Brapa_108**	**Thalo_62**	**Pvul_61**	**Gmax_129**	**Mtrunc_34**	**Rcomm_35**	**Mesc_49**	**Ptric_94**	**Mdom_72**		**Slyc_42**	**Nbent_74**
	Tcac_41		Athal_65	Brapa_99	Thalo_56		Gmax_131			Mesc_46	Ptric_101	Mdom_127			Nbent_77
			Athal_95	Brapa_103							Ptric_104	Mdom_133			
											Ptric_123	Mdom_200			
											Ptric_177	Mdom_201			
**Vvin_39**													**Csat_55**		
**Vvin_40**	**Tcac_53**						**Gmax_151**		**Rcomm_37**				**Csat_46**		
**Vvin_41**	**Tcac_34**	**Cpap_27**	**Athal_51**	**Brapa_83**	**Thalo_52**				**Rcomm_39**	**Mesc_68**	**Ptric_120**	**Mdom_70**			
	Tcac_36		Athal_50	Brapa_145							Ptric_107	Mdom_61			
	Tcac_105		Athal_52								Ptric_116	Mdom_151			
			Athal_53								Ptric_117	Mdom_173			
			Athal_54								Ptric_118	Mdom_193			
											Ptric_119	Mdom_213			
											Ptric_121	Mdom_217			
											Ptric_172	Mdom_229			
											Ptric_189				
											Ptric_202				
											Ptric_203				
											Ptric_204				
											Ptric_228				
											Ptric_229				
**Vvin_42**	**Tcac_49**		**Athal_63**	**Brapa_95**	**Thalo_57**	**Pvul_63**	**Gmax_137**	**Mtrunc_42**		**Mesc_72**	**Ptric_114**		**Csat_50**	**Slyc_46**	**Nbent_90**
	Tcac_58		Athal_130	Brapa_92	Thalo_106	Pvul_58	Gmax_127				Ptric_133		Csat_48	Slyc_55	Nbent_105
				Brapa_96	Thalo_110		Gmax_128				Ptric_134				Nbent_163
							Gmax_133				Ptric_135				Nbent_166
							Gmax_145								
							Gmax_147								
**Vvin_43**	**Tcac_45**	**Cpap_30**	**Athal_76**	**Brapa_120**	**Thalo_70**	**Pvul_60**	**Gmax_138**			**Mesc_64**	**Ptric_110**	**Mdom_74**		**Slyc_44**	**Nbent_82**
	Tcac_119	Cpap_77	Athal_100	Brapa_115		Pvul_62	Gmax_134			Mesc_69	Ptric_108	Mdom_131		Slyc_47	Nbent_91
			Athal_109	Brapa_116			Gmax_136				Ptric_185	Mdom_208		Slyc_83	Nbent_93
				Brapa_193			Gmax_139				Ptric_281			Slyc_85	Nbent_123
				Brapa_198							Ptric_286				Nbent_125
											Ptric_288				Nbent_126
															Nbent_202

Table S5 Continued

**Vvin_44**	**Tcac_35**		**Athal_60**	**Brapa_87**	**Thalo_53**	**Pvul_50**	**Gmax_114**			**Mesc_38**	**Ptric_77**	**Mdom_65**	**Csat_38**	**Slyc_33**	**Nbent_56**
			Athal_55	Brapa_88	Thalo_50	Pvul_65	Gmax_110				Ptric_183	Mdom_63	Csat_35	Slyc_38	Nbent_57
			Athal_61	Brapa_90	Thalo_51		Gmax_148					Mdom_150	Csat_39	Slyc_81	Nbent_59
			Athal_64	Brapa_93	Thalo_78							Mdom_245			Nbent_61
			Athal_70	Brapa_107											Nbent_150
			Athal_88	Brapa_135											Nbent_156
				Brapa_153											Nbent_174
															Nbent_184
															Nbent_185
**Vvin_45**	**Tcac_55**	**Cpap_31**	**Athal_80**	**Brapa_114**	**Thalo_66**	**Pvul_74**	**Gmax_140**	**Mtrunc_40**	**Rcomm_38**	**Mesc_58**	**Ptric_127**	**Mdom_89**	**Csat_53**	**Slyc_50**	**Nbent_89**
	Tcac_59	Cpap_57	Athal_79	Brapa_117	Thalo_65	Pvul_69	Gmax_142	Mtrunc_41	Rcomm_87	Mesc_57	Ptric_126	Mdom_109	Csat_52	Slyc_60	Nbent_81
	Tcac_62			Brapa_127	Thalo_67		Gmax_159	Mtrunc_46		Mesc_62	Ptric_128	Mdom_113	Csat_83	Slyc_87	
	Tcac_86			Brapa_154	Thalo_68		Gmax_160			Mesc_65	Ptric_129	Mdom_161			
	Tcac_93				Thalo_69		Gmax_187			Mesc_66	Ptric_130	Mdom_241			
	Tcac_113						Gmax_201				Ptric_131				
											Ptric_132				
											Ptric_136				
											Ptric_216				
**Vvin_46**	**Tcac_51**	**Cpap_33**	**Athal_74**	**Brapa_122**	**Thalo_72**	**Pvul_68**	**Gmax_146**		**Rcomm_57**	**Mesc_61**	**Ptric_111**	**Mdom_81**	**Csat_49**	**Slyc_49**	**Nbent_88**
	Tcac_50		Athal_75	Brapa_125	Thalo_74		Gmax_141			Mesc_60	Ptric_96	Mdom_80	Csat_54	Slyc_53	Nbent_78
	Tcac_52		Athal_77	Brapa_126	Thalo_77		Gmax_143				Ptric_98	Mdom_91	Csat_56		Nbent_80
	Tcac_80		Athal_78	Brapa_128			Gmax_144				Ptric_99	Mdom_105			Nbent_127
	Tcac_81		Athal_85	Brapa_132							Ptric_100	Mdom_156			
											Ptric_105	Mdom_179			
											Ptric_106	Mdom_185			
											Ptric_109	Mdom_228			
											Ptric_222				
											Ptric_249				
											Ptric_287				
**Vvin_47**				**Brapa_131**											
**Vvin_48**	**Tcac_64**	**Cpap_39**	**Athal_73**	**Brapa_118**	**Thalo_71**	**Pvul_70**	**Gmax_152**	**Mtrunc_48**	**Rcomm_42**	**Mesc_76**	**Ptric_143**	**Mdom_90**	**Csat_57**	**Slyc_52**	**Nbent_98**
	Tcac_63		Athal_81	Brapa_119	Thalo_73		Gmax_153		Rcomm_93		Ptric_274	Mdom_97	Csat_74	Slyc_54	Nbent_92
	Tcac_68		Athal_84	Brapa_124	Thalo_79										Nbent_94
			Athal_106	Brapa_130	Thalo_81										Nbent_97
				Brapa_133	Thalo_89										
				Brapa_166	Thalo_96										
				Brapa_169											
				Brapa_171											
				Brapa_196											
**Vvin_49**															
**Vvin_50**															
**Vvin_51**															
**Vvin_52**	**Tcac_61**	**Cpap_38**	**Athal_82**	**Brapa_121**	**Thalo_76**	**Pvul_73**	**Gmax_157**	**Mtrunc_52**	**Rcomm_43**	**Mesc_74**	**Ptric_145**	**Mdom_87**	**Csat_59**	**Slyc_56**	**Nbent_131**
				Brapa_129			Gmax_156			Mesc_77	Ptric_142	Mdom_88	Csat_60	Slyc_57	Nbent_116
				Brapa_134			Gmax_182				Ptric_144				

Table S5 Continued

**Vvin_53**										**Mesc_75**					
**Vvin_54**	**Tcac_47**								**Rcomm_41**	**Mesc_59**					
	Tcac_65								Rcomm_48	Mesc_73					
	Tcac_66									Mesc_84					
	Tcac_99									Mesc_103					
**Vvin_55**															
**Vvin_56**							**Gmax_161**								**Nbent_111**
							Gmax_165								Nbent_128
							Gmax_167								
**Vvin_57**															
**Vvin_58**	**Tcac_69**	**Cpap_43**	**Athal_86**	**Brapa_137**	**Thalo_80**	**Pvul_75**		**Mtrunc_56**	**Rcomm_44**	**Mesc_79**	**Ptric_147**	**Mdom_92**	**Csat_61**	**Slyc_59**	
			Athal_91	Brapa_138	Thalo_82	Pvul_78			Rcomm_45	Mesc_78	Ptric_150	Mdom_94	Csat_62		
			Athal_94	Brapa_141		Pvul_80			Rcomm_46	Mesc_81	Ptric_151	Mdom_96	Csat_63		
				Brapa_142						Mesc_82	Ptric_152	Mdom_98	Csat_65		
										Mesc_83	Ptric_190	Mdom_103			
										Mesc_87	Ptric_196	Mdom_112			
											Ptric_270	Mdom_134			
**Vvin_59**	**Tcac_73**	**Cpap_45**	**Athal_93**	**Brapa_146**	**Thalo_84**	**Pvul_79**	**Gmax_171**		**Rcomm_47**	**Mesc_90**	**Ptric_191**	**Mdom_114**	**Csat_70**	**Slyc_63**	**Nbent_113**
	Tcac_77			Brapa_148		Pvul_82	Gmax_172				Ptric_195	Mdom_123		Slyc_64	Nbent_130
				Brapa_149			Gmax_174				Ptric_198	Mdom_165			Nbent_133
							Gmax_175				Ptric_199				
							Gmax_176								
							Gmax_180								
**Vvin_60**															
**Vvin_61**										**Mesc_94**	**Ptric_187**				
											Ptric_193				
**Vvin_62**							**Gmax_191**				**Ptric_192**				
							Gmax_189				Ptric_200				
							Gmax_190								
**Vvin_63**	**Tcac_71**									**Mesc_85**	**Ptric_153**				
	Tcac_70										Ptric_154				
											Ptric_155				
											Ptric_156				
**Vvin_64**															

Table S5 Continued

	**Vvin_65**	**Tcac_110**	**Cpap_59**	**Athal_117**	**Brapa_179**	**Thalo_105**	**Pvul_92**	**Gmax_206**	**Mtrunc_69**	**Rcomm_74**	**Mesc_111**	**Ptric_241**	**Mdom_184**	**Csat_84**	**Slyc_71**	**Nbent_172**
		Tcac_108		Athal_118	Brapa_178	Thalo_107	Pvul_94	Gmax_205			Mesc_110	Ptric_235	Mdom_178	Csat_85		Nbent_165
		Tcac_109			Brapa_180		Pvul_95	Gmax_207				Ptric_237		Csat_87		Nbent_180
		Tcac_112					Pvul_96	Gmax_208				Ptric_238				
								Gmax_209				Ptric_239				
								Gmax_210				Ptric_240				
								Gmax_211								
								Gmax_212								
								Gmax_213								
								Gmax_214								
								Gmax_215								
								Gmax_216								
								Gmax_243								
5	**Vvin_66**	**Tcac_114**	**Cpap_62**	**Athal_114**	**Brapa_176**	**Thalo_103**	**Pvul_100**	**Gmax_226**		**Rcomm_75**	**Mesc_112**	**Ptric_236**				
					Brapa_175		Pvul_99	Gmax_217			Mesc_113	Ptric_243				
							Pvul_101	Gmax_220								
								Gmax_221								
								Gmax_225								
	**Vvin_67**	**Tcac_116**					**Pvul_103**	**Gmax_227**	**Mtrunc_68**	**Rcomm_77**	**Mesc_115**	**Ptric_244**	**Mdom_194**	**Csat_86**	**Slyc_73**	**Nbent_168**
							Pvul_97	Gmax_218	Mtrunc_72		Mesc_116	Ptric_242	Mdom_192	Csat_93		Nbent_167
							Pvul_98	Gmax_219				Ptric_245				
							Pvul_102	Gmax_222								
								Gmax_223								
								Gmax_224								
	**Vvin_68**	**Tcac_124**	**Cpap_63**	**Athal_121**	**Brapa_184**	**Thalo_108**	**Pvul_93**	**Gmax_229**	**Mtrunc_71**	**Rcomm_79**	**Mesc_119**	**Ptric_269**	**Mdom_198**	**Csat_94**	**Slyc_82**	**Nbent_178**
		Tcac_125			Brapa_185			Gmax_228	Mtrunc_70		Mesc_117	Ptric_263	Mdom_204			Nbent_177
								Gmax_230	Mtrunc_74			Ptric_265	Mdom_205			
								Gmax_231	Mtrunc_75			Ptric_266				
								Gmax_241				Ptric_267				
												Ptric_268				
	**Vvin_69**	**Tcac_126**		**Athal_124**	**Brapa_191**	**Thalo_113**	**Pvul_105**	**Gmax_239**		**Rcomm_83**	**Mesc_121**	**Ptric_276**	**Mdom_225**	**Csat_100**	**Slyc_79**	**Nbent_176**
				Athal_119	Brapa_183	Thalo_104	Pvul_106	Gmax_232			Mesc_120	Ptric_275		Csat_99		
								Gmax_233				Ptric_278				
								Gmax_234								
								Gmax_235								
								Gmax_236								
								Gmax_238								
								Gmax_240								
								Gmax_242								
								Gmax_244								
								Gmax_245								
								Gmax_246								
	**Vvin_70**															
	**Vvin_71**									**Rcomm_82**	**Mesc_123**	**Ptric_252**				
												Ptric_250				
												Ptric_251				
												Ptric_253				
												Ptric_254				
												Ptric_255				
												Ptric_256				
												Ptric_257				
