# Supplementary material for: Comparative Genomics of NAC Transcriptional Factors in Angiosperms: Implications for the Adaptation and Diversification of Flowering Plants
Source: PLoS One. 2015 Nov 16;10(11):e0141866. doi: 10.1371/journal.pone.0141866 (PMC4646352; doi:10.1371/journal.pone.0141866)
Supplement: S6 Table — List of 31 NAC OG proteins in basal angiosperm species. Sequences of rice and grapevine are marked in red and purple boxes, respectively. Sequences with the reciprocal BBH are shown in colored boxes. Paralogous sequences are shown below the colored boxes. The five BOGs are marked with yellow stars. (PDF) [file pone.0141866.s011.pdf]

Table S6

OGs proteins shared among basal angiosperm species. Sequences of rice and grapevine are marked in red and purple colored boxes respectively. BBH are shown in colored boxes. Paralogous sequences are shown below colored boxes. The five BOG are marked with a yellow star.

BOG (star)	BBH Osat	BBH Vvin	Paralogous proteins (Osat)	Paralogous proteins (Vvin)
4	Osat_1	Vvin_4	Osat_3, Osat_25, Osat_26, Osat_28, Osat_56, Osat_86	Vvin_2, Vvin_3, Vvin_24, Vvin_49, Vvin_70
	Osat_20	Vvin_5	Osat_9, Osat_12, Osat_13, Osat_14, Osat_38, Osat_39	
	Osat_15	Vvin_8	Osat_24	Vvin_6
	Osat_5	Vvin_9	Osat_71	Vvin_7
	Osat_8	Vvin_10		
2	Osat_2	Vvin_11	Osat_46	Vvin_14, Vvin_29
	Osat_4	Vvin_13	Osat_10, Osat_34, Osat_93	Vvin_12, Vvin_54, Vvin_61
	Osat_11	Vvin_15		
	Osat_6	Vvin_17	Osat_63, Osat_101	
1	Osat_7	Vvin_18		Vvin_16
	Osat_17	Vvin_22	Osat_85	
	Osat_32	Vvin_23	Osat_31, Osat_40	
	Osat_23	Vvin_25	Osat_61	Vvin_28
	Osat_18	Vvin_26		Vvin_21
	Osat_21	Vvin_27	Osat_22	
	Osat_33	Vvin_30	Osat_105	
	Osat_30	Vvin_31		Vvin_33
3	Osat_44	Vvin_34	Osat_49	
	Osat_36	Vvin_35	Osat_37, Osat_59	Vvin_44
	Osat_53	Vvin_38	Osat_41, Osat_58	
	Osat_52	Vvin_41	Osat_60	Vvin_1, Vvin_36, Vvin_51, Vvin_55, Vvin_57
	Osat_50	Vvin_42	Osat_88, Osat_96, Osat_104	Vvin_37, Vvin_39, Vvin_40, Vvin_47, Vvin_50
	Osat_42	Vvin_43	Osat_45, Osat_51, Osat_54, Osat_55	
	Osat_47	Vvin_45		Vvin_48
	Osat_62	Vvin_46		
	Osat_48	Vvin_52	Osat_57	Vvin_53
	Osat_65	Vvin_56	Osat_66, Osat_67, Osat_68, Osat_69	
	Osat_73	Vvin_59	Osat_77	
	Osat_87	Vvin_65	Osat_91, Osat_95	
5	Osat_94	Vvin_66		
	Osat_102	Vvin_69		
