# Supplementary material for: Comparative Genomics of NAC Transcriptional Factors in Angiosperms: Implications for the Adaptation and Diversification of Flowering Plants
Source: PLoS One. 2015 Nov 16;10(11):e0141866. doi: 10.1371/journal.pone.0141866 (PMC4646352; doi:10.1371/journal.pone.0141866)
Supplement: S4 Fig — The tree was arranged in six major clades (I-VI) and subclassified in minor groups. Major clades are highlighted in different colors. The ID sequences for moss P. patens are shown in blue, for S. moellendorffii in pink, for monocots in red and for eudicots in green. (PDF) [file pone.0141866.s004.pdf]

Figure S4

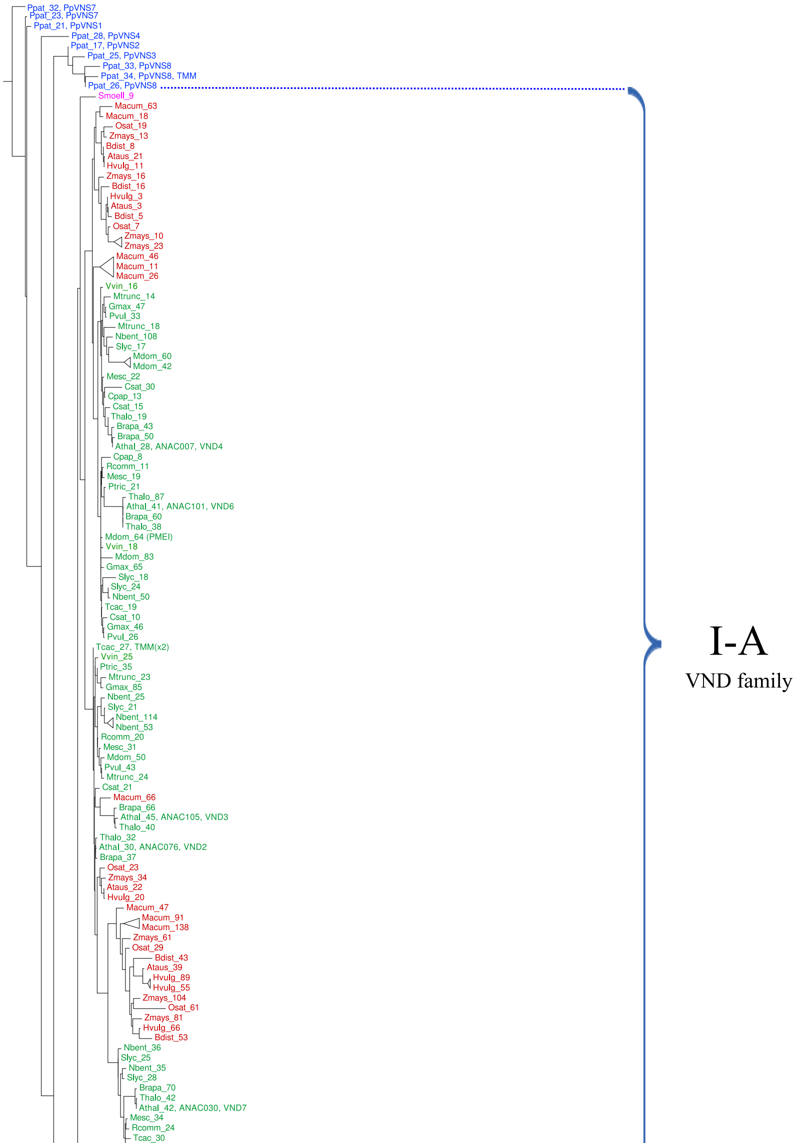
Ppat_32, PpVNS7
Ppat_23, PpVNS7
Ppat_21, PpVNS1
Ppat_28, PpVNS4
Ppat_17, PpVNS2
Ppat_25, PpVNS3
Ppat_33, PpVNS8
Ppat_34, PpVNS8, TMM
Ppat_26, PpVNS8
Smoell_9
Macum_63
Macum_18
Osat_19
Zmays_13
Bdist_8
Ataus_21
Hvulg_11
Zmays_16
Bdist_16
Hvulg_3
Ataus_3
Bdist_5
Osat_7
Zmays_10
Zmays_23
Macum_46
Macum_11
Macum_26
Vvin_16
Mtrunc_14
Gmax_47
Pvul_33
Mtrunc_18
Nbent_108
Slyc_17
Mdom_60
Mdom_42
Mesc_22
Csat_30
Cpap_13
Csat_15
Thalo_19
Brapa_43
Brapa_50
Athal_28, ANAC007, VND4
Cpap_8
Rcomm_11
Mesc_19
Ptric_21
Thalo_87
Athal_41, ANAC101, VND6
Brapa_60
Thalo_38
Mdom_64 (PMEI)
Vvin_18
Mdom_83
Gmax_65
Slyc_18
Slyc_24
Nbent_50
Tcac_19
Csat_10
Gmax_46
Pvul_26
Tcac_27, TMM(x2)
Vvin_25
Ptric_35
Mtrunc_23
Gmax_85
Nbent_25
Slyc_21
Nbent_114
Nbent_53
Rcomm_20
Mesc_31
Mdom_50
Pvul_43
Mtrunc_24
Csat_21
Macum_66
Brapa_66
Athal_45, ANAC105, VND3
Thalo_40
Thalo_32
Athal_30, ANAC076, VND2
Brapa_37
Osat_23
Zmays_34
Ataus_22
Hvulg_20
Macum_47
Macum_91
Macum_138
Zmays_61
Osat_29
Bdist_43
Ataus_39
Hvulg_89
Hvulg_55
Zmays_104
Osat_61
Zmays_81
Hvulg_66
Bdist_53
Nbent_36
Slyc_25
Nbent_35
Slyc_28
Brapa_70
Thalo_42
Athal_42, ANAC030, VND7
Mesc_34
Rcomm_24
Tcac_30
I-A
VND family

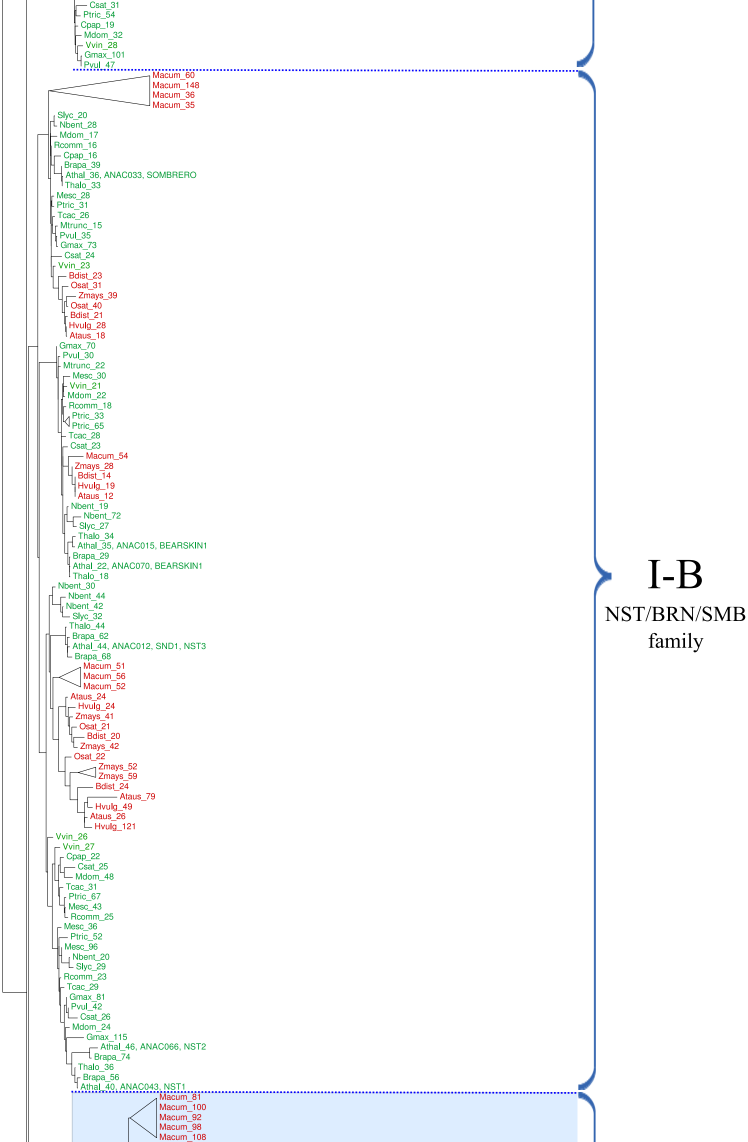
Csat_31
Ptric_54
Cpap_19
Mdom_32
Vvin_28
Gmax_101
Pvul_47
Macum_60
Macum_148
Macum_36
Macum_35
Slyc_20
Nbent_28
Mdom_17
Rcomm_16
Cpap_16
Brapa_39
Athal_36, ANAC033, SOMBRERO
Thalo_33
Mesc_28
Ptric_31
Tcac_26
Mtrunc_15
Pvul_35
Gmax_73
Csat_24
Vvin_23
Bdist_23
Osat_31
Zmays_39
Osat_40
Bdist_21
Hvulg_28
Ataus_18
Gmax_70
Pvul_30
Mtrunc_22
Mesc_30
Vvin_21
Mdom_22
Rcomm_18
Ptric_33
Ptric_65
Tcac_28
Csat_23
Macum_54
Zmays_28
Bdist_14
Hvulg_19
Ataus_12
Nbent_19
Nbent_72
Slyc_27
Thalo_34
Athal_35, ANAC015, BEARSKIN1
Brapa_29
Athal_22, ANAC070, BEARSKIN1
Thalo_18
Nbent_30
Nbent_44
Nbent_42
Slyc_32
Thalo_44
Brapa_62
Athal_44, ANAC012, SND1, NST3
Brapa_68
Macum_51
Macum_56
Macum_52
Ataus_24
Hvulg_24
Zmays_41
Osat_21
Bdist_20
Zmays_42
Osat_22
Zmays_52
Zmays_59
Bdist_24
Ataus_79
Hvulg_49
Ataus_26
Hvulg_121
Vvin_26
Vvin_27
Cpap_22
Csat_25
Mdom_48
Tcac_31
Ptric_67
Mesc_43
Rcomm_25
Mesc_36
Ptric_52
Mesc_96
Nbent_20
Slyc_29
Rcomm_23
Tcac_29
Gmax_81
Pvul_42
Csat_26
Mdom_24
Gmax_115
Athal_46, ANAC066, NST2
Brapa_74
Thalo_36
Brapa_56
Athal_40, ANAC043, NST1
Macum_81
Macum_100
Macum_92
Macum_98
Macum_108
I-B
NST/BRN/SMB
family

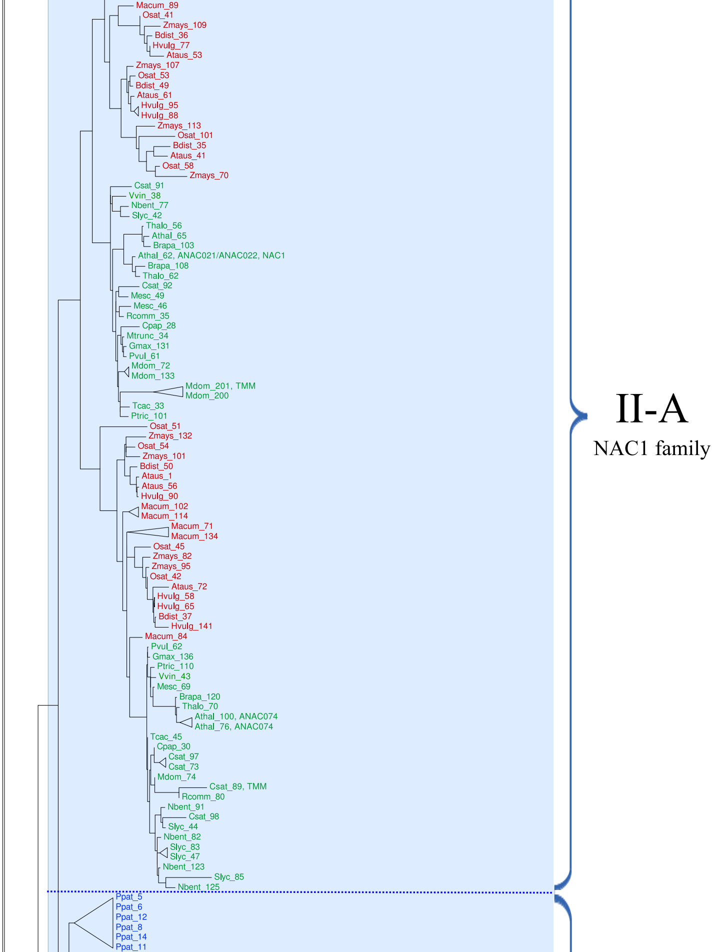
Macum_89
Osat_41
Zmays_109
Bdist_36
Hvulg_77
Ataus_53
Zmays_107
Osat_53
Bdist_49
Ataus_61
Hvulg_95
Hvulg_88
Zmays_113
Osat_101
Bdist_35
Ataus_41
Osat_58
Zmays_70
Csat_91
Vvin_38
Nbent_77
Slyc_42
Thalo_56
Athal_65
Brapa_103
Athal_62, ANAC021/ANAC022, NAC1
Brapa_108
Thalo_62
Csat_92
Mesc_49
Mesc_46
Rcomm_35
Cpap_28
Mtrunc_34
Gmax_131
Pvul_61
Mdom_72
Mdom_133
Mdom_201, TMM
Mdom_200
Tcac_33
Ptric_101
Osat_51
Zmays_132
Osat_54
Zmays_101
Bdist_50
Ataus_1
Ataus_56
Hvulg_90
Macum_102
Macum_114
Macum_71
Macum_134
Osat_45
Zmays_82
Zmays_95
Osat_42
Ataus_72
Hvulg_58
Hvulg_65
Bdist_37
Hvulg_141
Macum_84
Pvul_62
Gmax_136
Ptric_110
Vvin_43
Mesc_69
Brapa_120
Thalo_70
Athal_100, ANAC074
Athal_76, ANAC074
Tcac_45
Cpap_30
Csat_97
Csat_73
Mdom_74
Csat_89, TMM
Rcomm_80
Nbent_91
Csat_98
Slyc_44
Nbent_82
Slyc_83
Slyc_47
Nbent_123
Slyc_85
Nbent_125
II-A
NAC1 family
Ppat_5
Ppat_6
Ppat_12
Ppat_8
Ppat_14
Ppat_11

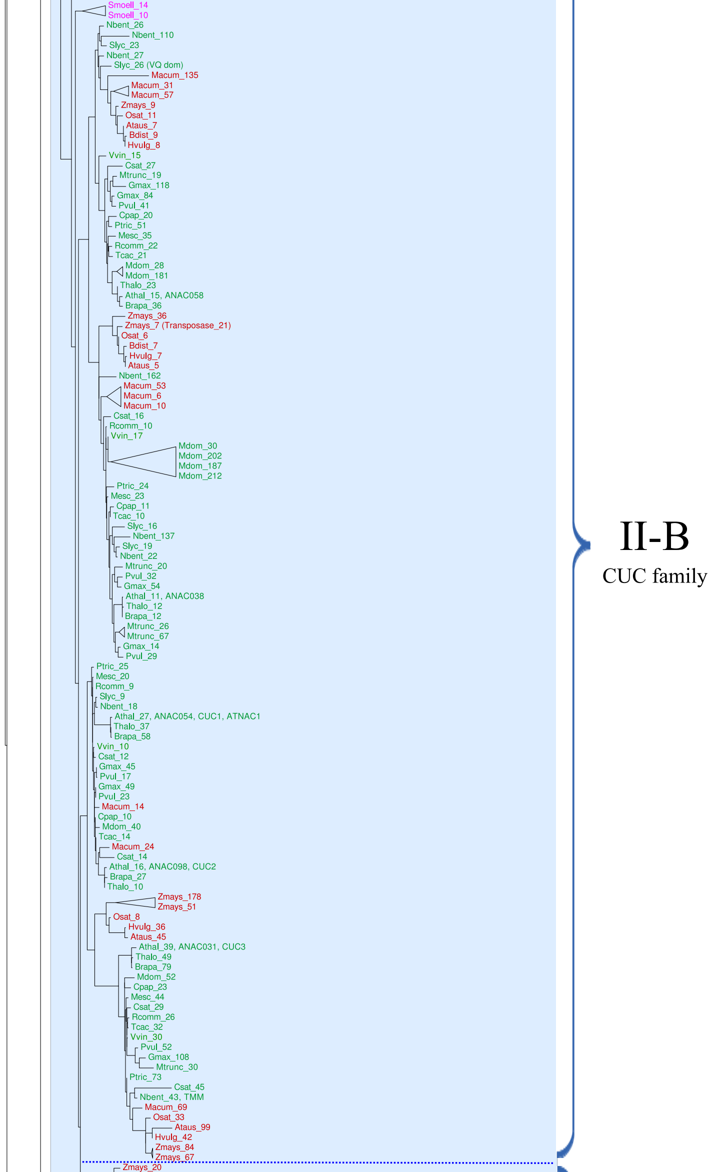
Smoell_14
Smoell_10
Nbent_26
Nbent_110
Slyc_23
Nbent_27
Slyc_26 (VQ dom)
Macum_135
Macum_31
Macum_57
Zmays_9
Osat_11
Ataus_7
Bdist_9
Hvulg_8
Vvin_15
Csat_27
Mtrunc_19
Gmax_118
Gmax_84
Pvul_41
Cpap_20
Ptric_51
Mesc_35
Rcomm_22
Tcac_21
Mdom_28
Mdom_181
Thalo_23
Athal_15, ANAC058
Brapa_36
Zmays_36
Zmays_7 (Transposase_21)
Osat_6
Bdist_7
Hvulg_7
Ataus_5
Nbent_162
Macum_53
Macum_6
Macum_10
Csat_16
Rcomm_10
Vvin_17
Mdom_30
Mdom_202
Mdom_187
Mdom_212
Ptric_24
Mesc_23
Cpap_11
Tcac_10
Slyc_16
Nbent_137
Slyc_19
Nbent_22
Mtrunc_20
Pvul_32
Gmax_54
Athal_11, ANAC038
Thalo_12
Brapa_12
Mtrunc_26
Mtrunc_67
Gmax_14
Pvul_29
Ptric_25
Mesc_20
Rcomm_9
Slyc_9
Nbent_18
Athal_27, ANAC054, CUC1, ATNAC1
Thalo_37
Brapa_58
Vvin_10
Csat_12
Gmax_45
Pvul_17
Gmax_49
Pvul_23
Macum_14
Cpap_10
Mdom_40
Tcac_14
Macum_24
Csat_14
Athal_16, ANAC098, CUC2
Brapa_27
Thalo_10
Zmays_178
Zmays_51
Osat_8
Hvulg_36
Ataus_45
Athal_39, ANAC031, CUC3
Thalo_49
Brapa_79
Mdom_52
Cpap_23
Mesc_44
Csat_29
Rcomm_26
Tcac_32
Vvin_30
Pvul_52
Gmax_108
Mtrunc_30
Ptric_73
Csat_45
Nbent_43, TMM
Macum_69
Osat_33
Ataus_99
Hvulg_42
Zmays_84
Zmays_67
Zmays_20
II-B
CUC family

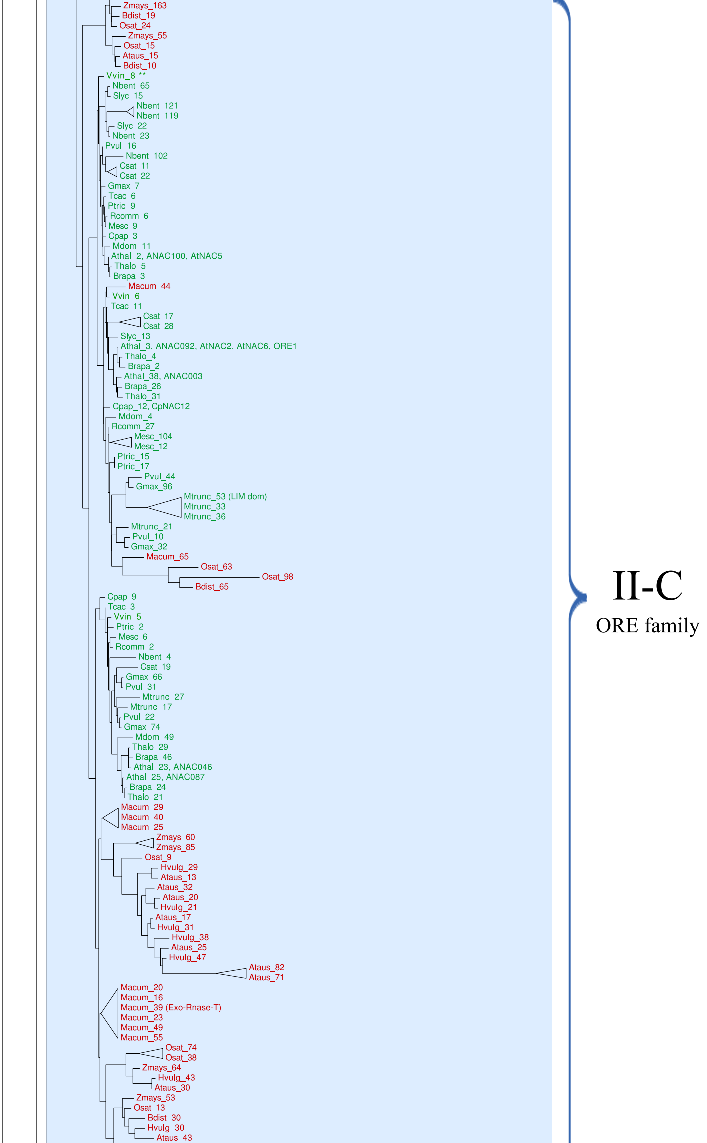
Zmays_163
Bdist_19
Osat_24
Zmays_55
Osat_15
Ataus_15
Bdist_10
Vvin_8 **
Nbent_65
Slyc_15
Nbent_121
Nbent_119
Slyc_22
Nbent_23
Pvul_16
Nbent_102
Csat_11
Csat_22
Gmax_7
Tcac_6
Ptric_9
Rcomm_6
Mesc_9
Cpap_3
Mdom_11
Athal_2, ANAC100, AtNAC5
Thalo_5
Brapa_3
Macum_44
Vvin_6
Tcac_11
Csat_17
Csat_28
Slyc_13
Athal_3, ANAC092, AtNAC2, AtNAC6, ORE1
Thalo_4
Brapa_2
Athal_38, ANAC003
Brapa_26
Thalo_31
Cpap_12, CpNAC12
Mdom_4
Rcomm_27
Mesc_104
Mesc_12
Ptric_15
Ptric_17
Pvul_44
Gmax_96
Mtrunc_53 (LIM dom)
Mtrunc_33
Mtrunc_36
Mtrunc_21
Pvul_10
Gmax_32
Macum_65
Osat_63
Osat_98
Bdist_65
Cpap_9
Tcac_3
Vvin_5
Ptric_2
Mesc_6
Rcomm_2
Nbent_4
Csat_19
Gmax_66
Pvul_31
Mtrunc_27
Mtrunc_17
Pvul_22
Gmax_74
Mdom_49
Thalo_29
Brapa_46
Athal_23, ANAC046
Athal_25, ANAC087
Brapa_24
Thalo_21
Macum_29
Macum_40
Macum_25
Zmays_60
Zmays_85
Osat_9
Hvulg_29
Ataus_13
Ataus_32
Ataus_20
Hvulg_21
Ataus_17
Hvulg_31
Hvulg_38
Ataus_25
Hvulg_47
Ataus_82
Ataus_71
Macum_20
Macum_16
Macum_39 (Exo-Rnase-T)
Macum_23
Macum_49
Macum_55
Osat_74
Osat_38
Zmays_64
Hvulg_43
Ataus_30
Zmays_53
Osat_13
Bdist_30
Hvulg_30
Ataus_43
II-C
ORE family

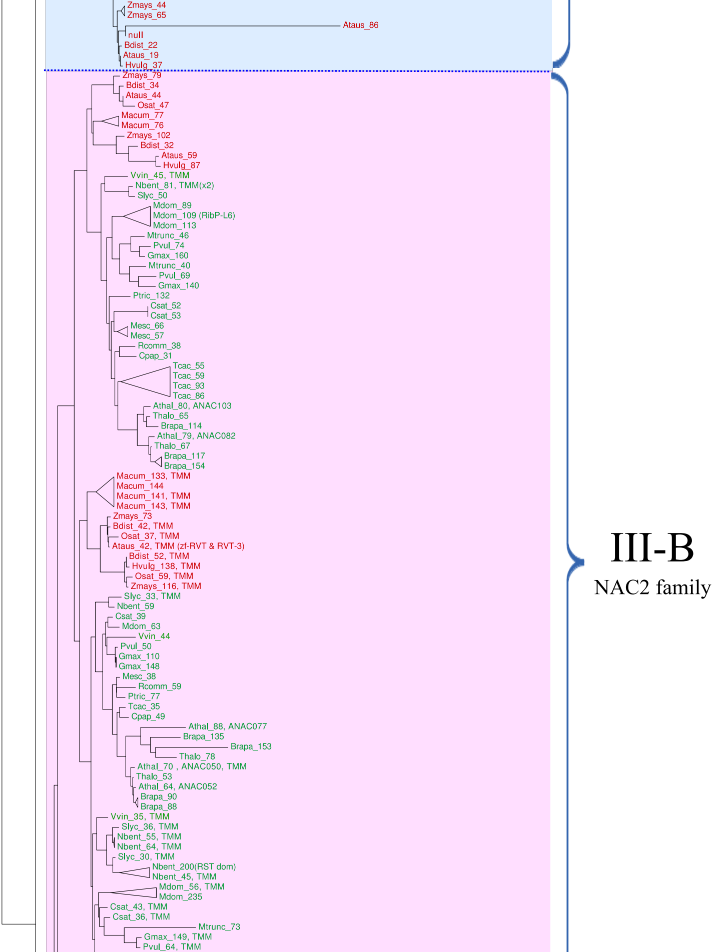

Zmays_44
Zmays_65
Ataus_86
null
Bdist_22
Ataus_19
Hvulg_37
Zmays_79
Bdist_34
Ataus_44
Osat_47
Macum_77
Macum_76
Zmays_102
Bdist_32
Ataus_59
Hvulg_87
Vvin_45, TMM
Nbent_81, TMM(x2)
Slyc_50
Mdom_89
Mdom_109 (RibP-L6)
Mdom_113
Mtrunc_46
Pvul_74
Gmax_160
Mtrunc_40
Pvul_69
Gmax_140
Ptric_132
Csat_52
Csat_53
Mesc_66
Mesc_57
Rcomm_38
Cpap_31
Tcac_55
Tcac_59
Tcac_93
Tcac_86
Athal_80, ANAC103
Thalo_65
Brapa_114
Athal_79, ANAC082
Thalo_67
Brapa_117
Brapa_154
Macum_133, TMM
Macum_144
Macum_141, TMM
Macum_143, TMM
Zmays_73
Bdist_42, TMM
Osat_37, TMM
Ataus_42, TMM (zf-RVT & RVT-3)
Bdist_52, TMM
Hvulg_138, TMM
Osat_59, TMM
Zmays_116, TMM
Slyc_33, TMM
Nbent_59
Csat_39
Mdom_63
Vvin_44
Pvul_50
Gmax_110
Gmax_148
Mesc_38
Rcomm_59
Ptric_77
Tcac_35
Cpap_49
Athal_88, ANAC077
Brapa_135
Brapa_153
Thalo_78
Athal_70 , ANAC050, TMM
Thalo_53
Athal_64, ANAC052
Brapa_90
Brapa_88
Vvin_35, TMM
Slyc_36, TMM
Nbent_55, TMM
Nbent_64, TMM
Slyc_30, TMM
Nbent_200(RST dom)
Nbent_45, TMM
Mdom_56, TMM
Mdom_235
Csat_43, TMM
Csat_36, TMM
Mtrunc_73
Gmax_149, TMM
Pvul_64, TMM
III-B
NAC2 family

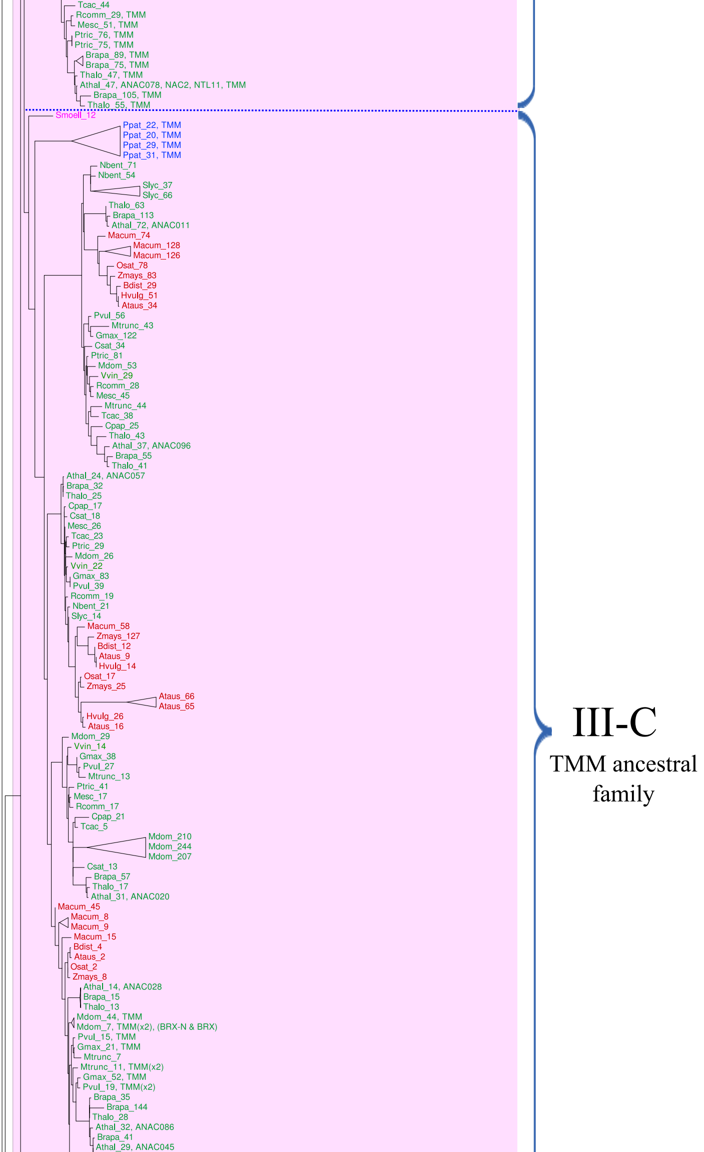
Tcac_44
Rcomm_29, TMM
Mesc_51, TMM
Ptric_76, TMM
Ptric_75, TMM
Brapa_89, TMM
Brapa_75, TMM
Thalo_47, TMM
Athal_47, ANAC078, NAC2, NTL11, TMM
Brapa_105, TMM
Thalo_55, TMM
Smoell_12
Ppat_22, TMM
Ppat_20, TMM
Ppat_29, TMM
Ppat_31, TMM
Nbent_71
Nbent_54
Slyc_37
Slyc_66
Thalo_63
Brapa_113
Athal_72, ANAC011
Macum_74
Macum_128
Macum_126
Osat_78
Zmays_83
Bdist_29
Hvulg_51
Ataus_34
Pvul_56
Mtrunc_43
Gmax_122
Csat_34
Ptric_81
Mdom_53
Vvin_29
Rcomm_28
Mesc_45
Mtrunc_44
Tcac_38
Cpap_25
Thalo_43
Athal_37, ANAC096
Brapa_55
Thalo_41
Athal_24, ANAC057
Brapa_32
Thalo_25
Cpap_17
Csat_18
Mesc_26
Tcac_23
Ptric_29
Mdom_26
Vvin_22
Gmax_83
Pvul_39
Rcomm_19
Nbent_21
Slyc_14
Macum_58
Zmays_127
Bdist_12
Ataus_9
Hvulg_14
Osat_17
Zmays_25
Ataus_66
Ataus_65
Hvulg_26
Ataus_16
Mdom_29
Vvin_14
Gmax_38
Pvul_27
Mtrunc_13
Ptric_41
Mesc_17
Rcomm_17
Cpap_21
Tcac_5
Mdom_210
Mdom_244
Mdom_207
Csat_13
Brapa_57
Thalo_17
Athal_31, ANAC020
Macum_45
Macum_8
Macum_9
Macum_15
Bdist_4
Ataus_2
Osat_2
Zmays_8
Athal_14, ANAC028
Brapa_15
Thalo_13
Mdom_44, TMM
Mdom_7, TMM(x2), (BRX-N & BRX)
Pvul_15, TMM
Gmax_21, TMM
Mtrunc_7
Mtrunc_11, TMM(x2)
Gmax_52, TMM
Pvul_19, TMM(x2)
Brapa_35
Brapa_144
Thalo_28
Athal_32, ANAC086
Brapa_41
Athal_29, ANAC045
III-C
TMM ancestral
family

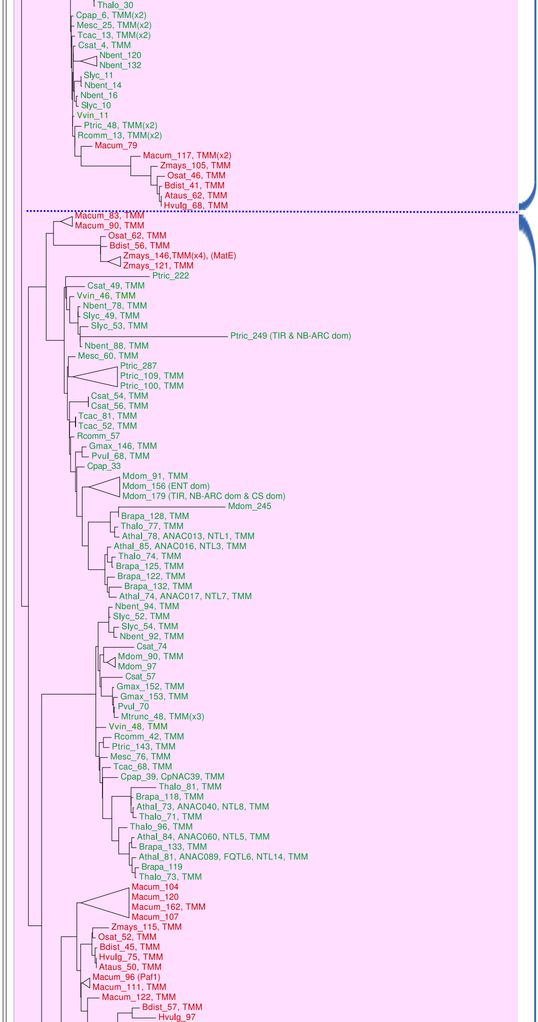
Thalo_30
Cpap_6, TMM(x2)
Mesc_25, TMM(x2)
Tcac_13, TMM(x2)
Csat_4, TMM
Nbent_120
Nbent_132
Slyc_11
Nbent_14
Nbent_16
Slyc_10
Vvin_11
Ptric_48, TMM(x2)
Rcomm_13, TMM(x2)
Macum_79
Macum_117, TMM(x2)
Zmays_105, TMM
Osat_46, TMM
Bdist_41, TMM
Ataus_62, TMM
Hvulg_68, TMM
Macum_83, TMM
Macum_90, TMM
Osat_62, TMM
Bdist_56, TMM
Zmays_146,TMM(x4), (MatE)
Zmays_121, TMM
Ptric_222
Csat_49, TMM
Vvin_46, TMM
Nbent_78, TMM
Slyc_49, TMM
Slyc_53, TMM
Ptric_249 (TIR & NB-ARC dom)
Nbent_88, TMM
Mesc_60, TMM
Ptric_287
Ptric_109, TMM
Ptric_100, TMM
Csat_54, TMM
Csat_56, TMM
Tcac_81, TMM
Tcac_52, TMM
Rcomm_57
Gmax_146, TMM
Pvul_68, TMM
Cpap_33
Mdom_91, TMM
Mdom_156 (ENT dom)
Mdom_179 (TIR, NB-ARC dom & CS dom)
Mdom_245
Brapa_128, TMM
Thalo_77, TMM
Athal_78, ANAC013, NTL1, TMM
Athal_85, ANAC016, NTL3, TMM
Thalo_74, TMM
Brapa_125, TMM
Brapa_122, TMM
Brapa_132, TMM
Athal_74, ANAC017, NTL7, TMM
Nbent_94, TMM
Slyc_52, TMM
Slyc_54, TMM
Nbent_92, TMM
Csat_74
Mdom_90, TMM
Mdom_97
Csat_57
Gmax_152, TMM
Gmax_153, TMM
Pvul_70
Mtrunc_48, TMM(x3)
Vvin_48, TMM
Rcomm_42, TMM
Ptric_143, TMM
Mesc_76, TMM
Tcac_68, TMM
Cpap_39, CpNAC39, TMM
Thalo_81, TMM
Brapa_118, TMM
Athal_73, ANAC040, NTL8, TMM
Thalo_71, TMM
Thalo_96, TMM
Athal_84, ANAC060, NTL5, TMM
Brapa_133, TMM
Athal_81, ANAC089, FQTL6, NTL14, TMM
Brapa_119
Thalo_73, TMM
Macum_104
Macum_120
Macum_162, TMM
Macum_107
Zmays_115, TMM
Osat_52, TMM
Bdist_45, TMM
Hvulg_75, TMM
Ataus_50, TMM
Macum_96 (Paf1)
Macum_111, TMM
Macum_122, TMM
Bdist_57, TMM
Hvulg_97

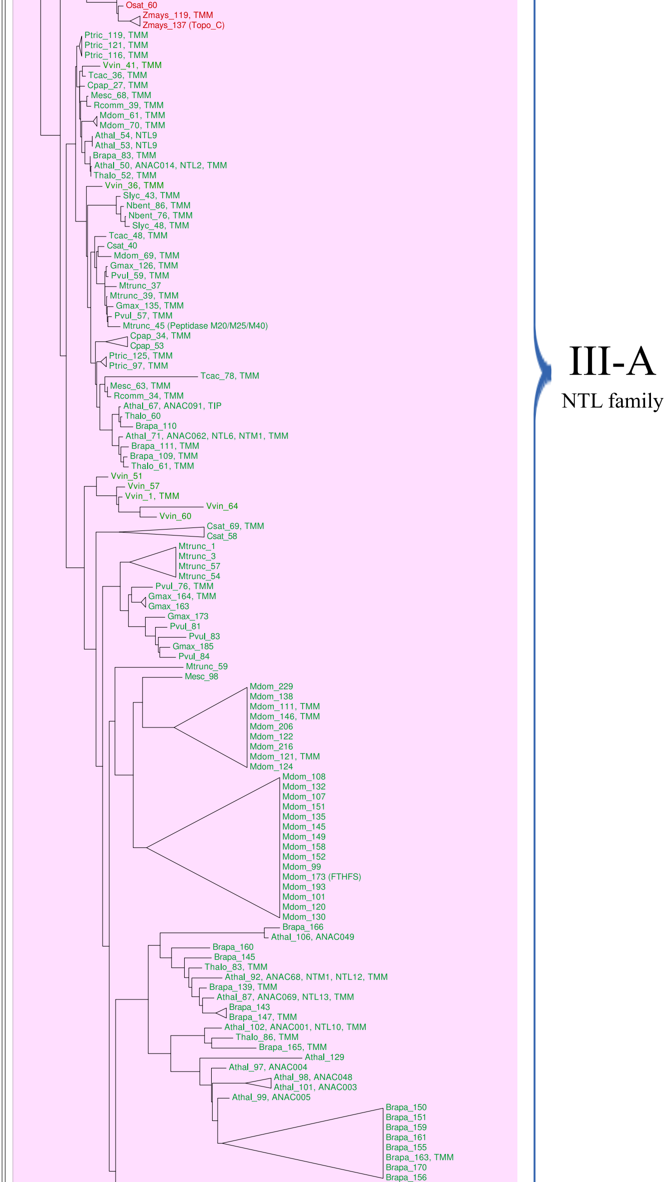
Osat_60
Zmays_119, TMM
Zmays_137 (Topo_C)
Ptric_119, TMM
Ptric_121, TMM
Ptric_116, TMM
Vvin_41, TMM
Tcac_36, TMM
Cpap_27, TMM
Mesc_68, TMM
Rcomm_39, TMM
Mdom_61, TMM
Mdom_70, TMM
Athal_54, NTL9
Athal_53, NTL9
Brapa_83, TMM
Athal_50, ANAC014, NTL2, TMM
Thalo_52, TMM
Vvin_36, TMM
Slyc_43, TMM
Nbent_86, TMM
Nbent_76, TMM
Slyc_48, TMM
Tcac_48, TMM
Csat_40
Mdom_69, TMM
Gmax_126, TMM
Pvul_59, TMM
Mtrunc_37
Mtrunc_39, TMM
Gmax_135, TMM
Pvul_57, TMM
Mtrunc_45 (Peptidase M20/M25/M40)
Cpap_34, TMM
Cpap_53
Ptric_125, TMM
Ptric_97, TMM
Tcac_78, TMM
Mesc_63, TMM
Rcomm_34, TMM
Athal_67, ANAC091, TIP
Thalo_60
Brapa_110
Athal_71, ANAC062, NTL6, NTM1, TMM
Brapa_111, TMM
Brapa_109, TMM
Thalo_61, TMM
Vvin_51
Vvin_57
Vvin_1, TMM
Vvin_64
Vvin_60
Csat_69, TMM
Csat_58
Mtrunc_1
Mtrunc_3
Mtrunc_57
Mtrunc_54
Pvul_76, TMM
Gmax_164, TMM
Gmax_163
Gmax_173
Pvul_81
Pvul_83
Gmax_185
Pvul_84
Mtrunc_59
Mesc_98
Mdom_229
Mdom_138
Mdom_111, TMM
Mdom_146, TMM
Mdom_206
Mdom_122
Mdom_216
Mdom_121, TMM
Mdom_124
Mdom_108
Mdom_132
Mdom_107
Mdom_151
Mdom_135
Mdom_145
Mdom_149
Mdom_158
Mdom_152
Mdom_99
Mdom_173 (FTHFS)
Mdom_193
Mdom_101
Mdom_120
Mdom_130
Brapa_166
Athal_106, ANAC049
Brapa_160
Brapa_145
Thalo_83, TMM
Athal_92, ANAC68, NTM1, NTL12, TMM
Brapa_139, TMM
Athal_87, ANAC069, NTL13, TMM
Brapa_143
Brapa_147, TMM
Athal_102, ANAC001, NTL10, TMM
Thalo_86, TMM
Brapa_165, TMM
Athal_129
Athal_97, ANAC004
Athal_98, ANAC048
Athal_101, ANAC003
Athal_99, ANAC005
Brapa_150
Brapa_151
Brapa_159
Brapa_161
Brapa_155
Brapa_163, TMM
Brapa_170
Brapa_156
III-A
NTL family

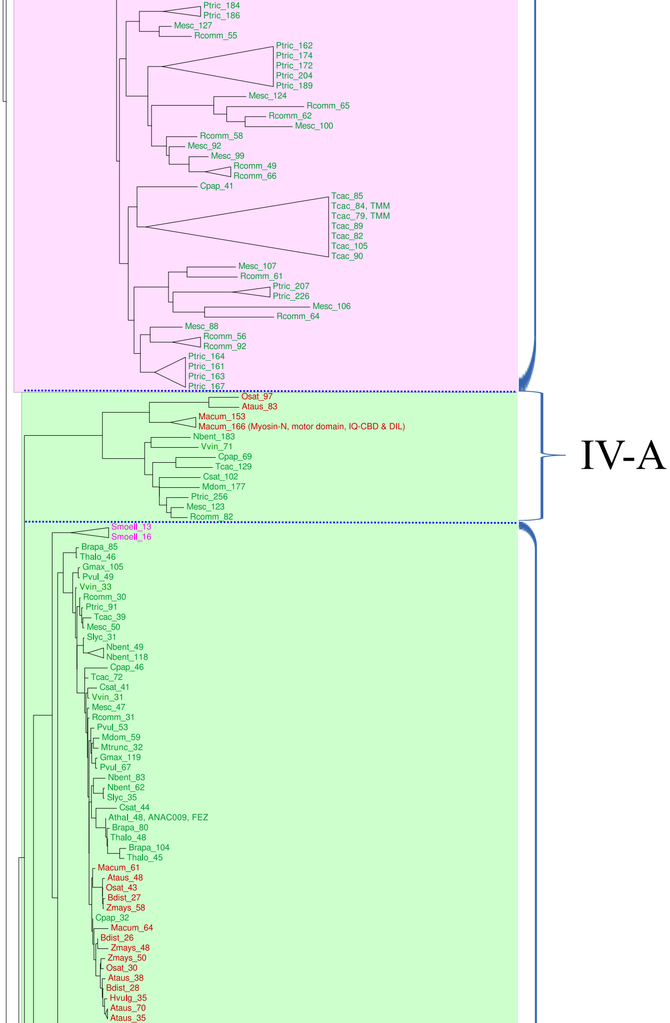
Ptric_184
Ptric_186
Mesc_127
Rcomm_55
Ptric_162
Ptric_174
Ptric_172
Ptric_204
Ptric_189
Mesc_124
Rcomm_65
Rcomm_62
Mesc_100
Rcomm_58
Mesc_92
Mesc_99
Rcomm_49
Rcomm_66
Cpap_41
Tcac_85
Tcac_84, TMM
Tcac_79, TMM
Tcac_89
Tcac_82
Tcac_105
Tcac_90
Mesc_107
Rcomm_61
Ptric_207
Ptric_226
Mesc_106
Rcomm_64
Mesc_88
Rcomm_56
Rcomm_92
Ptric_164
Ptric_161
Ptric_163
Ptric_167
Osat_97
Ataus_83
Macum_153
Macum_166 (Myosin-N, motor domain, IQ-CBD & DIL)
Nbent_183
Vvin_71
Cpap_69
Tcac_129
Csat_102
Mdom_177
Ptric_256
Mesc_123
Rcomm_82
IV-A
Smoell_13
Smoell_16
Brapa_85
Thalo_46
Gmax_105
Pvul_49
Vvin_33
Rcomm_30
Ptric_91
Tcac_39
Mesc_50
Slyc_31
Nbent_49
Nbent_118
Cpap_46
Tcac_72
Csat_41
Vvin_31
Mesc_47
Rcomm_31
Pvul_53
Mdom_59
Mtrunc_32
Gmax_119
Pvul_67
Nbent_83
Nbent_62
Slyc_35
Csat_44
Athal_48, ANAC009, FEZ
Brapa_80
Thalo_48
Brapa_104
Thalo_45
Macum_61
Ataus_48
Osat_43
Bdist_27
Zmays_58
Cpap_32
Macum_64
Bdist_26
Zmays_48
Zmays_50
Osat_30
Ataus_38
Bdist_28
Hvulg_35
Ataus_70
Ataus_35

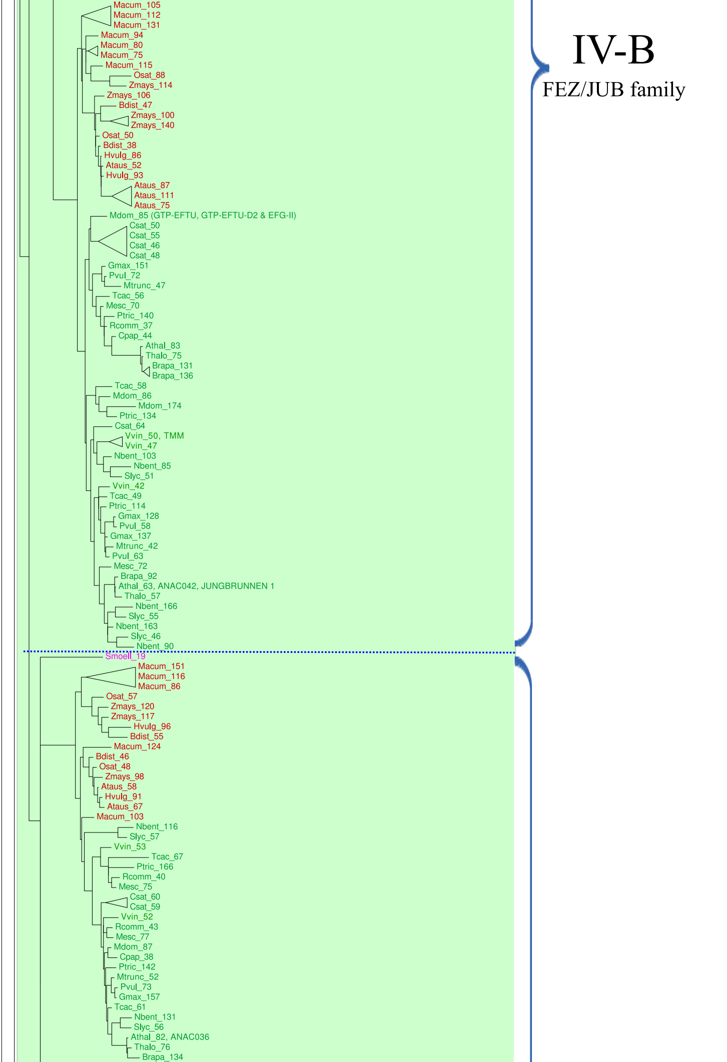
IV-B
FEZ/JUB family
Macum_105
Macum_112
Macum_131
Macum_94
Macum_80
Macum_75
Macum_115
Osat_88
Zmays_114
Zmays_106
Bdist_47
Zmays_100
Zmays_140
Osat_50
Bdist_38
Hvulg_86
Ataus_52
Hvulg_93
Ataus_87
Ataus_111
Ataus_75
Mdom_85 (GTP-EFTU, GTP-EFTU-D2 & EFG-II)
Csat_50
Csat_55
Csat_46
Csat_48
Gmax_151
Pvul_72
Mtrunc_47
Tcac_56
Mesc_70
Ptric_140
Rcomm_37
Cpap_44
Athal_83
Thalo_75
Brapa_131
Brapa_136
Tcac_58
Mdom_86
Mdom_174
Ptric_134
Csat_64
Vvin_50, TMM
Vvin_47
Nbent_103
Nbent_85
Slyc_51
Vvin_42
Tcac_49
Ptric_114
Gmax_128
Pvul_58
Gmax_137
Mtrunc_42
Pvul_63
Mesc_72
Brapa_92
Athal_63, ANAC042, JUNGBRUNNEN 1
Thalo_57
Nbent_166
Slyc_55
Nbent_163
Slyc_46
Nbent_90
Smoell_19
Macum_151
Macum_116
Macum_86
Osat_57
Zmays_120
Zmays_117
Hvulg_96
Bdist_55
Macum_124
Bdist_46
Osat_48
Zmays_98
Ataus_58
Hvulg_91
Ataus_67
Macum_103
Nbent_116
Slyc_57
Vvin_53
Tcac_67
Ptric_166
Rcomm_40
Mesc_75
Csat_60
Csat_59
Vvin_52
Rcomm_43
Mesc_77
Mdom_87
Cpap_38
Ptric_142
Mtrunc_52
Pvul_73
Gmax_157
Tcac_61
Nbent_131
Slyc_56
Athal_82, ANAC036
Thalo_76
Brapa_134

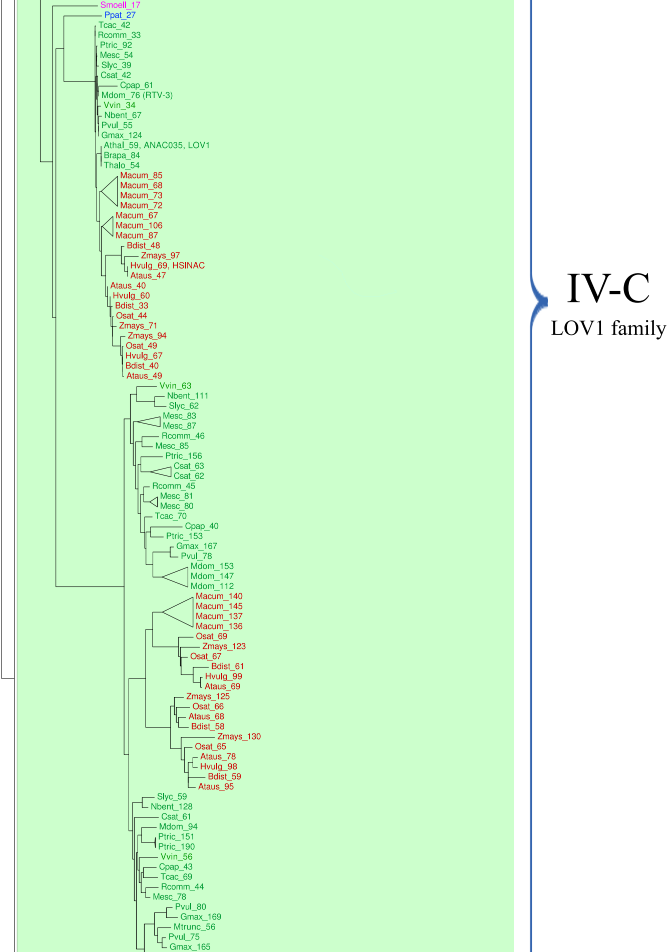
Smoell_17
Ppat_27
Tcac_42
Rcomm_33
Ptric_92
Mesc_54
Slyc_39
Csat_42
Cpap_61
Mdom_76 (RTV-3)
Vvin_34
Nbent_67
Pvul_55
Gmax_124
Athal_59, ANAC035, LOV1
Brapa_84
Thalo_54
Macum_85
Macum_68
Macum_73
Macum_72
Macum_67
Macum_106
Macum_87
Bdist_48
Zmays_97
Hvulg_69, HSINAC
Ataus_47
Ataus_40
Hvulg_60
Bdist_33
Osat_44
Zmays_71
Zmays_94
Osat_49
Hvulg_67
Bdist_40
Ataus_49
Vvin_63
Nbent_111
Slyc_62
Mesc_83
Mesc_87
Rcomm_46
Mesc_85
Ptric_156
Csat_63
Csat_62
Rcomm_45
Mesc_81
Mesc_80
Tcac_70
Cpap_40
Ptric_153
Gmax_167
Pvul_78
Mdom_153
Mdom_147
Mdom_112
Macum_140
Macum_145
Macum_137
Macum_136
Osat_69
Zmays_123
Osat_67
Bdist_61
Hvulg_99
Ataus_69
Zmays_125
Osat_66
Ataus_68
Bdist_58
Zmays_130
Osat_65
Ataus_78
Hvulg_98
Bdist_59
Ataus_95
Slyc_59
Nbent_128
Csat_61
Mdom_94
Ptric_151
Ptric_190
Vvin_56
Cpap_43
Tcac_69
Rcomm_44
Mesc_78
Pvul_80
Gmax_169
Mtrunc_56
Pvul_75
Gmax_165
IV-C
LOV1 family

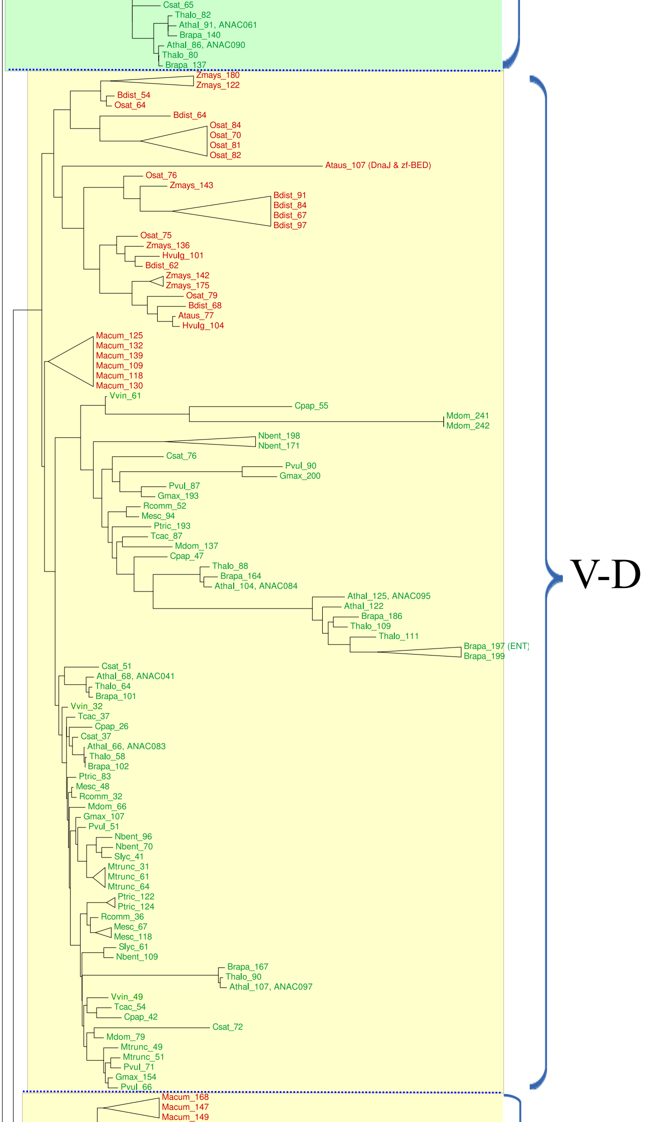

Csat_65
Thalo_82
Athal_91, ANAC061
Brapa_140
Athal_86, ANAC090
Thalo_80
Brapa_137
Zmays_180
Zmays_122
Bdist_54
Osat_64
Bdist_64
Osat_84
Osat_70
Osat_81
Osat_82
Ataus_107 (DnaJ & zf-BED)
Osat_76
Zmays_143
Bdist_91
Bdist_84
Bdist_67
Bdist_97
Osat_75
Zmays_136
Hvulg_101
Bdist_62
Zmays_142
Zmays_175
Osat_79
Bdist_68
Ataus_77
Hvulg_104
Macum_125
Macum_132
Macum_139
Macum_109
Macum_118
Macum_130
Vvin_61
Cpap_55
Mdom_241
Mdom_242
Nbent_198
Nbent_171
Csat_76
Pvul_90
Gmax_200
Pvul_87
Gmax_193
Rcomm_52
Mesc_94
Ptric_193
Tcac_87
Mdom_137
Cpap_47
Thalo_88
Brapa_164
Athal_104, ANAC084
Athal_125, ANAC095
Athal_122
Brapa_186
Thalo_109
Thalo_111
Brapa_197 (ENT)
Brapa_199
Csat_51
Athal_68, ANAC041
Thalo_64
Brapa_101
Vvin_32
Tcac_37
Cpap_26
Csat_37
Athal_66, ANAC083
Thalo_58
Brapa_102
Ptric_83
Mesc_48
Rcomm_32
Mdom_66
Gmax_107
Pvul_51
Nbent_96
Nbent_70
Slyc_41
Mtrunc_31
Mtrunc_61
Mtrunc_64
Ptric_122
Ptric_124
Rcomm_36
Mesc_67
Mesc_118
Slyc_61
Nbent_109
Brapa_167
Thalo_90
Athal_107, ANAC097
Vvin_49
Tcac_54
Cpap_42
Csat_72
Mdom_79
Mtrunc_49
Mtrunc_51
Pvul_71
Gmax_154
Pvul_66
Macum_168
Macum_147
Macum_149
V-D

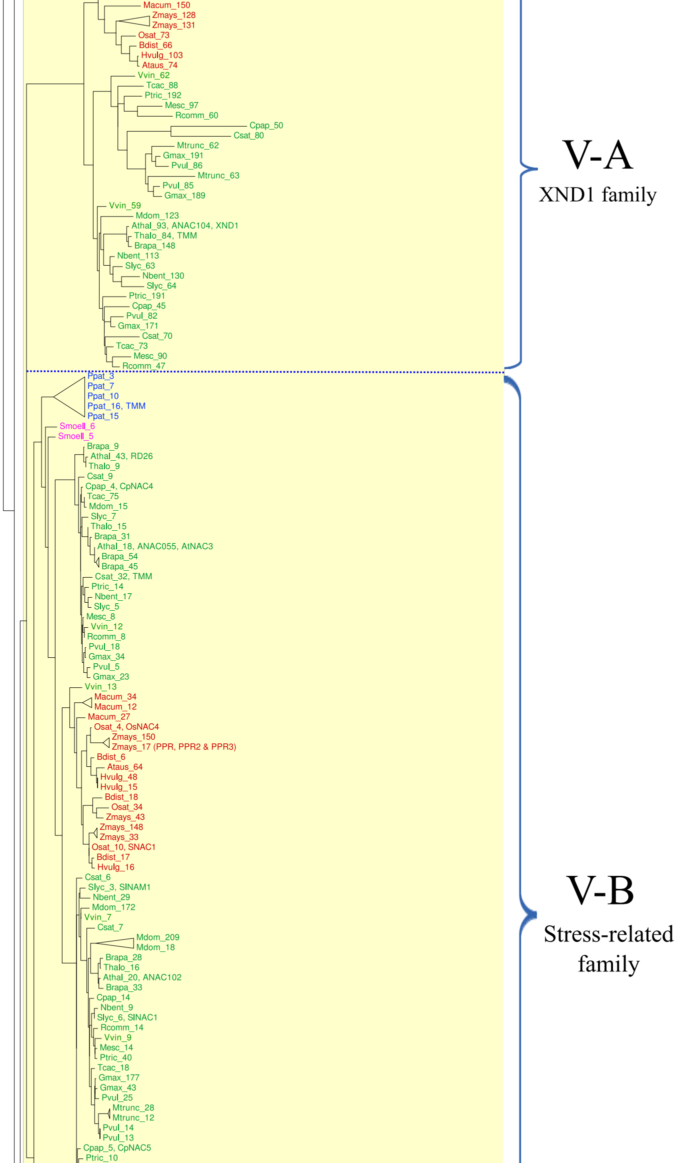
Macum_150
Zmays_128
Zmays_131
Osat_73
Bdist_66
Hvulg_103
Ataus_74
Vvin_62
Tcac_88
Ptric_192
Mesc_97
Rcomm_60
Cpap_50
Csat_80
Mtrunc_62
Gmax_191
Pvul_86
Mtrunc_63
Pvul_85
Gmax_189
Vvin_59
Mdom_123
Athal_93, ANAC104, XND1
Thalo_84, TMM
Brapa_148
Nbent_113
Slyc_63
Nbent_130
Slyc_64
Ptric_191
Cpap_45
Pvul_82
Gmax_171
Csat_70
Tcac_73
Mesc_90
Rcomm_47
Ppat_3
Ppat_7
Ppat_10
Ppat_16, TMM
Ppat_15
Smoell_6
Smoell_5
Brapa_9
Athal_43, RD26
Thalo_9
Csat_9
Cpap_4, CpNAC4
Tcac_75
Mdom_15
Slyc_7
Thalo_15
Brapa_31
Athal_18, ANAC055, AtNAC3
Brapa_54
Brapa_45
Csat_32, TMM
Ptric_14
Nbent_17
Slyc_5
Mesc_8
Vvin_12
Rcomm_8
Pvul_18
Gmax_34
Pvul_5
Gmax_23
Vvin_13
Macum_34
Macum_12
Macum_27
Osat_4, OsNAC4
Zmays_150
Zmays_17 (PPR, PPR2 & PPR3)
Bdist_6
Ataus_64
Hvulg_48
Hvulg_15
Bdist_18
Osat_34
Zmays_43
Zmays_148
Zmays_33
Osat_10, SNAC1
Bdist_17
Hvulg_16
Csat_6
Slyc_3, SlNAM1
Nbent_29
Mdom_172
Vvin_7
Csat_7
Mdom_209
Mdom_18
Brapa_28
Thalo_16
Athal_20, ANAC102
Brapa_33
Cpap_14
Nbent_9
Slyc_6, SINAC1
Rcomm_14
Vvin_9
Mesc_14
Ptric_40
Tcac_18
Gmax_177
Gmax_43
Pvul_25
Mtrunc_28
Mtrunc_12
Pvul_14
Pvul_13
Cpap_5, CpNAC5
Ptric_10
V-A
XND1 family
V-B
Stress-related
family

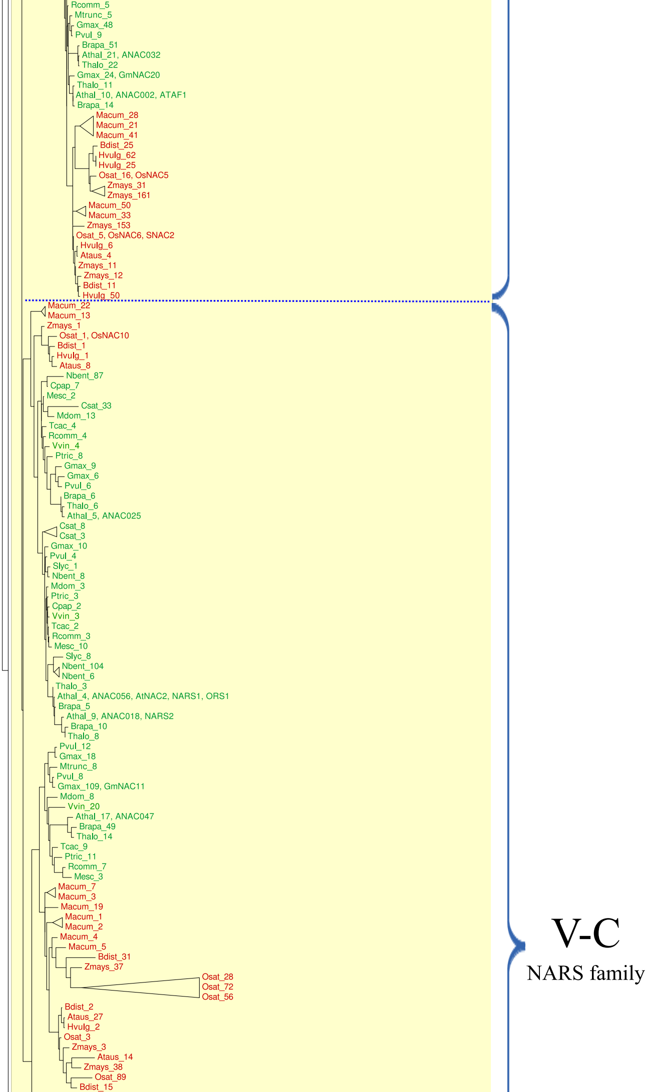

Rcomm_5
Mtrunc_5
Gmax_48
Pvul_9
Brapa_51
Athal_21, ANAC032
Thalo_22
Gmax_24, GmNAC20
Thalo_11
Athal_10, ANAC002, ATAF1
Brapa_14
Macum_28
Macum_21
Macum_41
Bdist_25
Hvulg_62
Hvulg_25
Osat_16, OsNAC5
Zmays_31
Zmays_161
Macum_50
Macum_33
Zmays_153
Osat_5, OsNAC6, SNAC2
Hvulg_6
Ataus_4
Zmays_11
Zmays_12
Bdist_11
Hvulg_50
Macum_22
Macum_13
Zmays_1
Osat_1, OsNAC10
Bdist_1
Hvulg_1
Ataus_8
Nbent_87
Cpap_7
Mesc_2
Csat_33
Mdom_13
Tcac_4
Rcomm_4
Vvin_4
Ptric_8
Gmax_9
Gmax_6
Pvul_6
Brapa_6
Thalo_6
Athal_5, ANAC025
Csat_8
Csat_3
Gmax_10
Pvul_4
Slyc_1
Nbent_8
Mdom_3
Ptric_3
Cpap_2
Vvin_3
Tcac_2
Rcomm_3
Mesc_10
Slyc_8
Nbent_104
Nbent_6
Thalo_3
Athal_4, ANAC056, AtNAC2, NARS1, ORS1
Brapa_5
Athal_9, ANAC018, NARS2
Brapa_10
Thalo_8
Pvul_12
Gmax_18
Mtrunc_8
Pvul_8
Gmax_109, GmNAC11
Mdom_8
Vvin_20
Athal_17, ANAC047
Brapa_49
Thalo_14
Tcac_9
Ptric_11
Rcomm_7
Mesc_3
Macum_7
Macum_3
Macum_19
Macum_1
Macum_2
Macum_4
Macum_5
Bdist_31
Zmays_37
Osat_28
Osat_72
Osat_56
Bdist_2
Ataus_27
Hvulg_2
Osat_3
Zmays_3
Ataus_14
Zmays_38
Osat_89
Bdist_15
V-C
NARS family

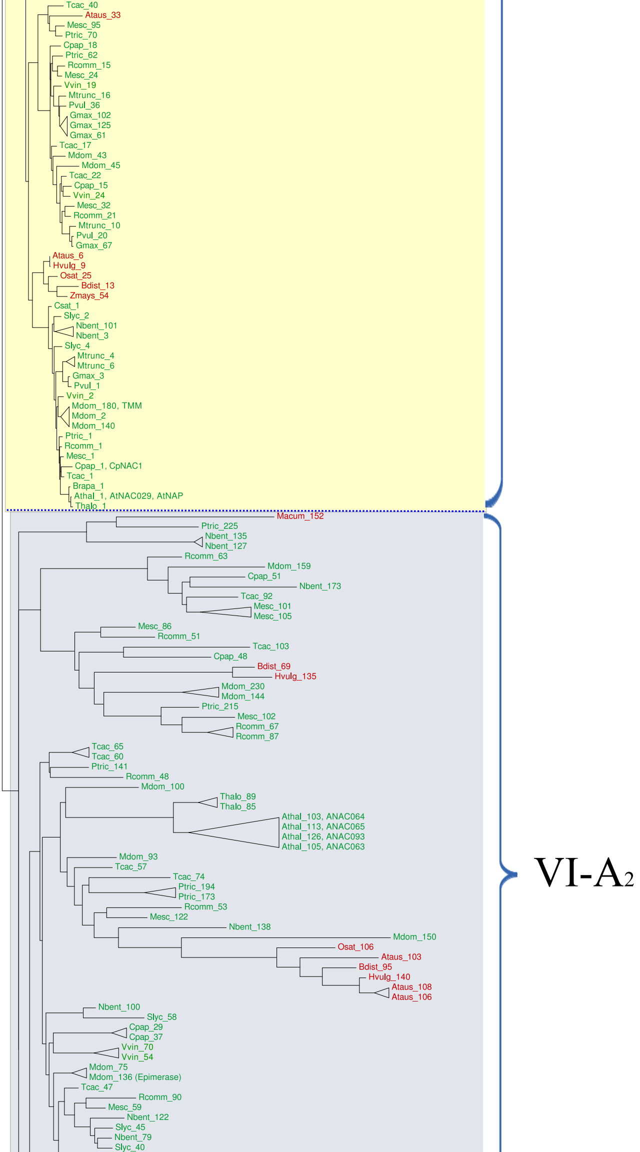

Tcac_40
Ataus_33
Mesc_95
Ptric_70
Cpap_18
Ptric_62
Rcomm_15
Mesc_24
Vvin_19
Mtrunc_16
Pvul_36
Gmax_102
Gmax_125
Gmax_61
Tcac_17
Mdom_43
Mdom_45
Tcac_22
Cpap_15
Vvin_24
Mesc_32
Rcomm_21
Mtrunc_10
Pvul_20
Gmax_67
Ataus_6
Hvulg_9
Osat_25
Bdist_13
Zmays_54
Csat_1
Slyc_2
Nbent_101
Nbent_3
Slyc_4
Mtrunc_4
Mtrunc_6
Gmax_3
Pvul_1
Vvin_2
Mdom_180, TMM
Mdom_2
Mdom_140
Ptric_1
Rcomm_1
Mesc_1
Cpap_1, CpNAC1
Tcac_1
Brapa_1
Athal_1, AtNAC029, AtNAP
Thalo_1
Macum_152
Ptric_225
Nbent_135
Nbent_127
Rcomm_63
Mdom_159
Cpap_51
Nbent_173
Tcac_92
Mesc_101
Mesc_105
Mesc_86
Rcomm_51
Tcac_103
Cpap_48
Bdist_69
Hvulg_135
Mdom_230
Mdom_144
Ptric_215
Mesc_102
Rcomm_67
Rcomm_87
Tcac_65
Tcac_60
Ptric_141
Rcomm_48
Mdom_100
Thalo_89
Thalo_85
Athal_103, ANAC064
Athal_113, ANAC065
Athal_126, ANAC093
Athal_105, ANAC063
Mdom_93
Tcac_57
Tcac_74
Ptric_194
Ptric_173
Rcomm_53
Mesc_122
Nbent_138
Mdom_150
Osat_106
Ataus_103
Bdist_95
Hvulg_140
Ataus_108
Ataus_106
Nbent_100
Slyc_58
Cpap_29
Cpap_37
Vvin_70
Vvin_54
Mdom_75
Mdom_136 (Epimerase)
Tcac_47
Rcomm_90
Mesc_59
Nbent_122
Slyc_45
Nbent_79
Slyc_40
VI-A2

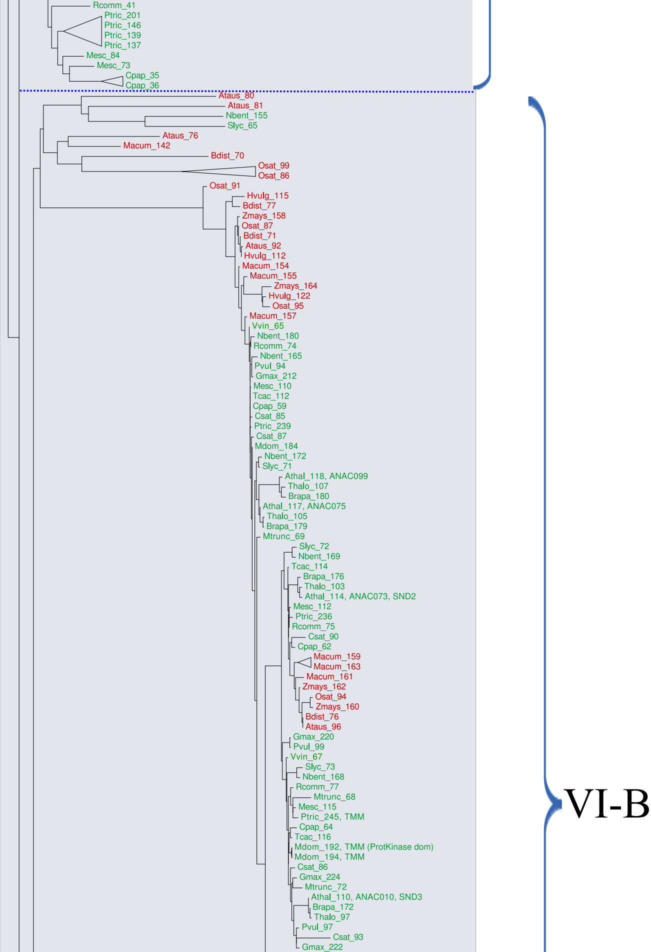

Rcomm_41
Ptric_201
Ptric_146
Ptric_139
Ptric_137
Mesc_84
Mesc_73
Cpap_35
Cpap_36
Ataus_80
Ataus_81
Nbent_155
Slyc_65
Ataus_76
Macum_142
Bdist_70
Osat_99
Osat_86
Osat_91
Hvulg_115
Bdist_77
Zmays_158
Osat_87
Bdist_71
Ataus_92
Hvulg_112
Macum_154
Macum_155
Zmays_164
Hvulg_122
Osat_95
Macum_157
Vvin_65
Nbent_180
Rcomm_74
Nbent_165
Pvul_94
Gmax_212
Mesc_110
Tcac_112
Cpap_59
Csat_85
Ptric_239
Csat_87
Mdom_184
Nbent_172
Slyc_71
Athal_118, ANAC099
Thalo_107
Brapa_180
Athal_117, ANAC075
Thalo_105
Brapa_179
Mtrunc_69
Slyc_72
Nbent_169
Tcac_114
Brapa_176
Thalo_103
Athal_114, ANAC073, SND2
Mesc_112
Ptric_236
Rcomm_75
Csat_90
Cpap_62
Macum_159
Macum_163
Macum_161
Zmays_162
Osat_94
Zmays_160
Bdist_76
Ataus_96
Gmax_220
Pvul_99
Vvin_67
Slyc_73
Nbent_168
Rcomm_77
Mtrunc_68
Mesc_115
Ptric_245, TMM
Cpap_64
Tcac_116
Mdom_192, TMM (ProtKinase dom)
Mdom_194, TMM
Csat_86
Gmax_224
Mtrunc_72
Athal_110, ANAC010, SND3
Brapa_172
Thalo_97
Pvul_97
Csat_93
Gmax_222
VI-B

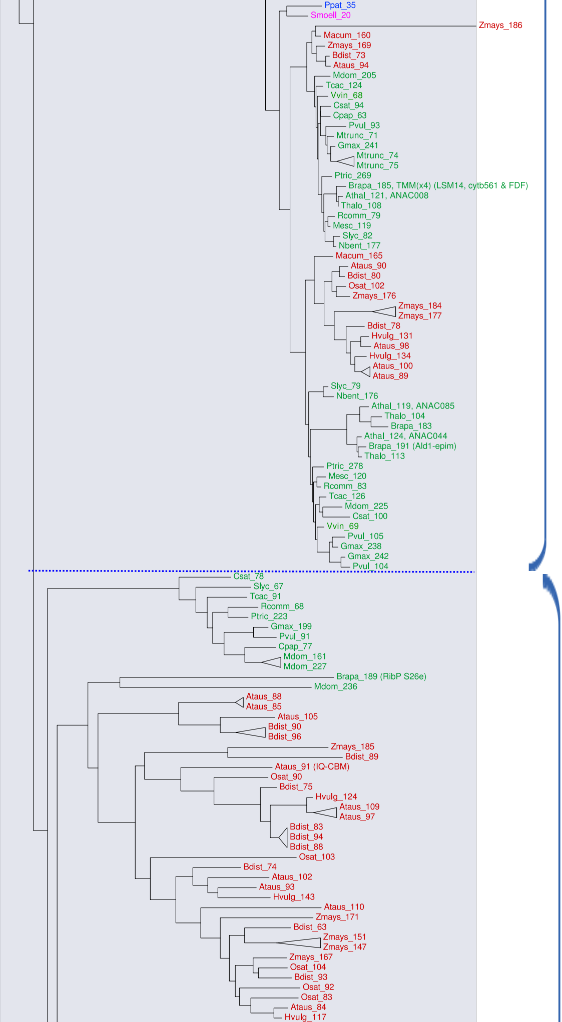

Ppat_35
Smoell_20
Zmays_186
Macum_160
Zmays_169
Bdist_73
Ataus_94
Mdom_205
Tcac_124
Vvin_68
Csat_94
Cpap_63
Pvul_93
Mtrunc_71
Gmax_241
Mtrunc_74
Mtrunc_75
Ptric_269
Brapa_185, TMM(x4) (LSM14, cytb561 & FDF)
Athal_121, ANAC008
Thalo_108
Rcomm_79
Mesc_119
Slyc_82
Nbent_177
Macum_165
Ataus_90
Bdist_80
Osat_102
Zmays_176
Zmays_184
Zmays_177
Bdist_78
Hvulg_131
Ataus_98
Hvulg_134
Ataus_100
Ataus_89
Slyc_79
Nbent_176
Athal_119, ANAC085
Thalo_104
Brapa_183
Athal_124, ANAC044
Brapa_191 (Ald1-epim)
Thalo_113
Ptric_278
Mesc_120
Rcomm_83
Tcac_126
Mdom_225
Csat_100
Vvin_69
Pvul_105
Gmax_238
Gmax_242
Pvul_104
Csat_78
Slyc_67
Tcac_91
Rcomm_68
Ptric_223
Gmax_199
Pvul_91
Cpap_77
Mdom_161
Mdom_227
Brapa_189 (RibP S26e)
Mdom_236
Ataus_88
Ataus_85
Ataus_105
Bdist_90
Bdist_96
Zmays_185
Bdist_89
Ataus_91 (IQ-CBM)
Osat_90
Bdist_75
Hvulg_124
Ataus_109
Ataus_97
Bdist_83
Bdist_94
Bdist_88
Osat_103
Bdist_74
Ataus_102
Ataus_93
Hvulg_143
Ataus_110
Zmays_171
Bdist_63
Zmays_151
Zmays_147
Zmays_167
Osat_104
Bdist_93
Osat_92
Osat_83
Ataus_84
Hvulg_117

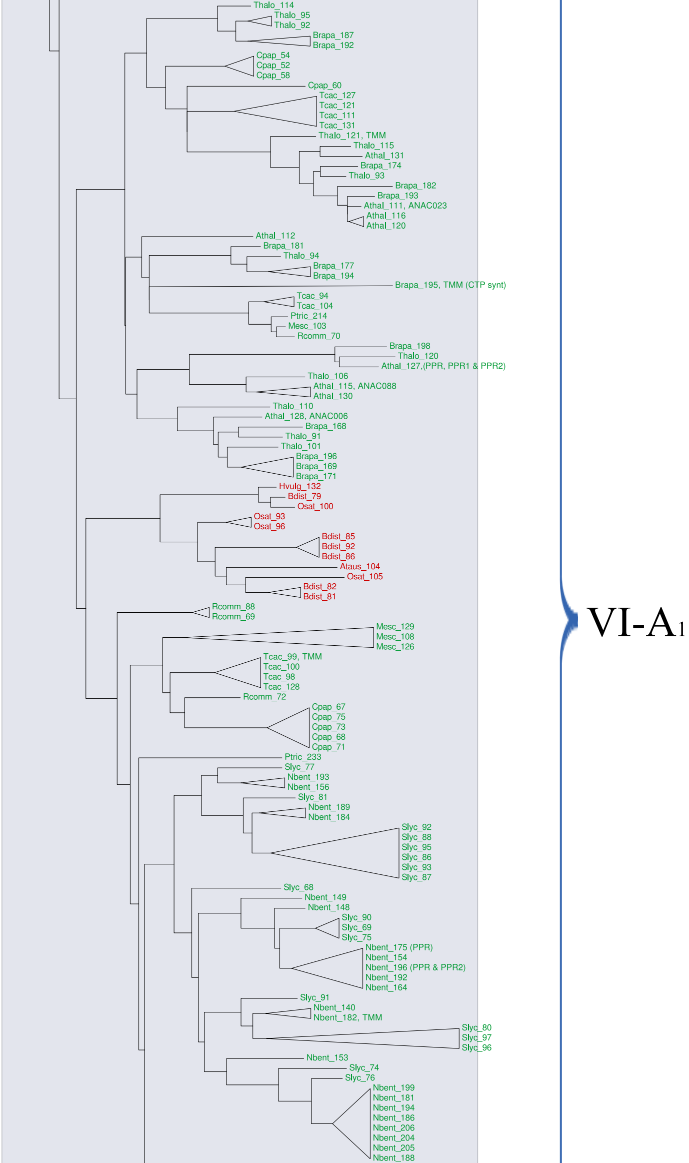
Thalo_114
Thalo_95
Thalo_92
Brapa_187
Brapa_192
Cpap_54
Cpap_52
Cpap_58
Cpap_60
Tcac_127
Tcac_121
Tcac_111
Tcac_131
Thalo_121, TMM
Thalo_115
Athal_131
Brapa_174
Thalo_93
Brapa_182
Brapa_193
Athal_111, ANAC023
Athal_116
Athal_120
Athal_112
Brapa_181
Thalo_94
Brapa_177
Brapa_194
Brapa_195, TMM (CTP synt)
Tcac_94
Tcac_104
Ptric_214
Mesc_103
Rcomm_70
Brapa_198
Thalo_120
Athal_127,(PPR, PPR1 & PPR2)
Thalo_106
Athal_115, ANAC088
Athal_130
Thalo_110
Athal_128, ANAC006
Brapa_168
Thalo_91
Thalo_101
Brapa_196
Brapa_169
Brapa_171
Hvulg_132
Bdist_79
Osat_100
Osat_93
Osat_96
Bdist_85
Bdist_92
Bdist_86
Ataus_104
Osat_105
Bdist_82
Bdist_81
Rcomm_88
Rcomm_69
Mesc_129
Mesc_108
Mesc_126
Tcac_99, TMM
Tcac_100
Tcac_98
Tcac_128
Rcomm_72
Cpap_67
Cpap_75
Cpap_73
Cpap_68
Cpap_71
Ptric_233
Slyc_77
Nbent_193
Nbent_156
Slyc_81
Nbent_189
Nbent_184
Slyc_92
Slyc_88
Slyc_95
Slyc_86
Slyc_93
Slyc_87
Slyc_68
Nbent_149
Nbent_148
Slyc_90
Slyc_69
Slyc_75
Nbent_175 (PPR)
Nbent_154
Nbent_196 (PPR & PPR2)
Nbent_192
Nbent_164
Slyc_91
Nbent_140
Nbent_182, TMM
Slyc_80
Slyc_97
Slyc_96
Nbent_153
Slyc_74
Slyc_76
Nbent_199
Nbent_181
Nbent_194
Nbent_186
Nbent_206
Nbent_204
Nbent_205
Nbent_188
VI-A1

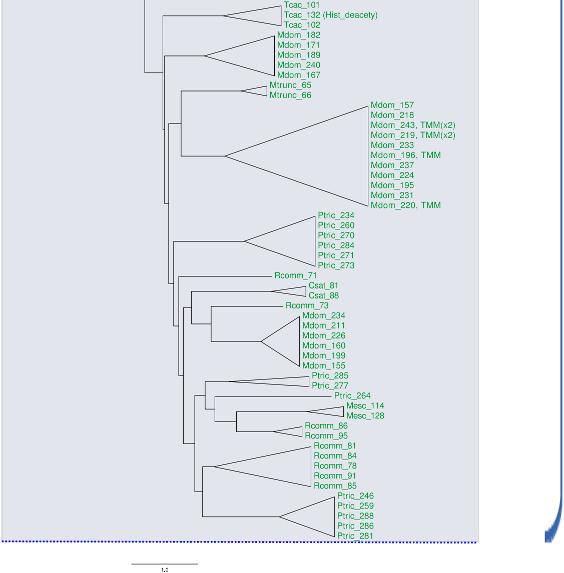

Maximum Likelihood phylogenetic tree of 2016 NAC transcription factors proteins of 24 green plants. *P. patens* sequences were used as outgroup, and all the sequences were arranged in six major groups. Species names are color coded as follows: Blue - *P. patens*, pink - *S. moellendorffii*, red - monocots, and green - eudicots.
